# The *MYCN* inhibitor BGA002 restores the retinoic acid response leading to differentiation or apoptosis by the mTOR block in *MYCN*-amplified neuroblastoma

**DOI:** 10.1186/s13046-022-02367-5

**Published:** 2022-04-30

**Authors:** Silvia Lampis, Salvatore Raieli, Luca Montemurro, Damiano Bartolucci, Camilla Amadesi, Sonia Bortolotti, Silvia Angelucci, Anna Lisa Scardovi, Giammario Nieddu, Lucia Cerisoli, Francesca Paganelli, Sabrina Valente, Matthias Fischer, Alberto Maria Martelli, Gianandrea Pasquinelli, Andrea Pession, Patrizia Hrelia, Roberto Tonelli

**Affiliations:** 1 R&D Department, BIOGENERA SpA, Bologna, Italy; 2 Pediatric Unit, S. Orsola IRCCS, University of Bologna, Bologna, Italy; 3 Department of Biomedical and Neuromotor Sciences, University of Bologna, Bologna, Italy; 4 CNR Institute of Molecular Genetics “Luigi Luca Cavalli-Sforza”, Unit of Bologna, Bologna, Italy; 5 Biotechnology and Methods in Laboratory Medicine, Department of Experimental, Diagnostic and Specialty Medicine (DIMES), University of Bologna, Bologna, Italy; 6 Department of Experimental Pediatric Oncology, University Children’s Hospital of Cologne, Medical Faculty, Cologne, Germany; and Center for Molecular Medicine Cologne (CMMC), University of Cologne, Cologne, Germany; 7 Subcellular nephro-vascular diagnostic program, Pathology Unit S. Orsola IRCCS, University of Bologna, Bologna, Italy; 8 Pediatric Unit, IRCCS, Azienda Ospedaliero-Universitaria di Bologna, Bologna, Italy; 9 Department of Pharmacy and Biotechnology, University of Bologna, Bologna, Italy

**Keywords:** Neuroblastoma, *MYCN*, Retinoic acid resistance, mTOR pathway, Differentiation

## Abstract

**Background:**

Neuroblastoma is a deadly childhood cancer, and *MYCN*-amplified neuroblastoma (MNA-NB) patients have the worst prognoses and are therapy-resistant. While retinoic acid (RA) is beneficial for some neuroblastoma patients, the cause of RA resistance is unknown. Thus, there remains a need for new therapies to treat neuroblastoma. Here we explored the possibility of combining a *MYCN*-specific antigene oligonucleotide BGA002 and RA as therapeutic approach to restore sensitivity to RA in NB.

**Methods:**

By molecular and cellular biology techniques, we assessed the combined effect of the two compounds in NB cell lines and in a xenograft mouse model MNA-NB.

**Results:**

We found that *MYCN*-specific inhibition by BGA002 in combination with RA (BGA002-RA) act synergistically and overcame resistance in NB cell lines. BGA002-RA also reactivated neuron differentiation (or led to apoptosis) and inhibited invasiveness capacity in MNA-NB. Moreover, we found that neuroblastoma had the highest level of mRNA expression of mTOR pathway genes, and that BGA002 led to mTOR pathway inhibition followed by autophagy reactivation in MNA-NB cells, which was strengthened by BGA002-RA. BGA002-RA in vivo treatment also eliminated tumor vascularization in a MNA-NB mouse model and significantly increased survival.

**Conclusion:**

Taken together, *MYCN* modulation mediates the therapeutic efficacy of RA and the development of RA resistance in MNA-NB. Furthermore, by targeting *MYCN*, a cancer-specific mTOR pathway inhibition occurs only in MNA-NB, thus avoiding the side effects of targeting mTOR in normal cells. These findings warrant clinical testing of BGA002-RA as a strategy for overcoming RA resistance in MNA-NB.

**Supplementary Information:**

The online version contains supplementary material available at 10.1186/s13046-022-02367-5.

## Background

Neuroblastoma is one of the deadliest cancers that occur in early childhood and represent 7% of pediatric malignancies [1]. Approximately 25% of patients with a neuroblastoma diagnosis present with *MYCN* amplification (MNA), which is linked to a poor prognosis, metastasis, and recurrence [2–5]. *MYCN* is a key driver of the disease and its overexpression reprograms neuroblastoma cells towards a stem-like phenotype that affects proliferation and cell growth, metabolism, and apoptosis inhibition. It also favors immune escape, invasion, and metastases [6–8].

Different therapeutic approaches have been developed to treat neuroblastoma, but high-risk cases (that are often *MYCN* amplified) remain critical. Among medical therapies, high-risk cases are treated with 13-*cis* retinoic acid (RA), which induces neuronal differentiation and leads to cell-growth inhibition [9]. *MYCN* expression needs to decrease to complete the differentiation program [10]; however, resistance to retinoic acid has been found, which is associated with concomitant relapses and poor survival outcomes [11].

Normally, *MYCN* expression is restricted during embryogenesis and is not expressed during adulthood [12]. Given its effect on neuroblastoma and its expression profile, N-Myc is a promising therapeutical target [13]. However, drug discovery approaches aimed at blocking N-Myc heterodimerization with MAX or its binding with DNA (without interfering with the highly homologous Myc) has, to-date, largely failed [13]. While indirect strategies have been proposed, due to the broad role, along with the number of pathways affected by its overexpression, N-Myc remains challenging to target. We have previously demonstrated that an alternative approach concerns specific gene expression inhibition at the level of DNA through a *MYCN*-specific antigene peptide nucleic acid (agPNA) oligonucleotide [14–16]. The antigene oligonucleotide approach (via persistent blocking at the level of transcription) has shown advantages in blocking translation by antisense strategies. PNAs have shown promising results as antigenes due to their resistance to proteases and nucleases and their ability to specifically bind target DNA [17, 18]. In particular, the *MYCN* specific agPNA, BGA002, is able to inhibit *MYCN* expression and block different *MYCN* tumorigenic alterations [16].

In this work we demonstrated that blocking *MYCN* in addition with RA, overcome RA resistance, allowing the MNA NB cell line differentiation. Moreover, we showed the joint treatment led to mTOR pathway blocking with concomitant autophagy restoration. Therefore, this induced an augmented survival in vivo xenograft mice.

## Material and methods

### Cell lines

Cell lines were obtained from different sources including: DSMZ (KELLY, LAN-5, CHP-134, SiMa, MHH-NB-11, NGP, LS, NMB, LAN-1, LAN-6, and NBL-S) and ECACC (SK-N-F1). In addition, the following cell lines were kindly gifted by the Gaslini Institute, Genova (GI-LI-N and SMS-KAN), Professor Della Valle G (IMR-32, SK-N-BE(2)-C, and TET-21N), and by Professor Spampinato SM (SH-SY5Y). For maintenance, the cell lines were stored in liquid nitrogen and kept in culture for a maximum of 30 days after thawing and no more than 7 passages (average 3) from the time they were obtained. We verified the presence of *Mycoplasma* every 3 months using the Look Out Mycoplasma PCR Detection Kit (Sigma Aldrich; Merck KGaA, Darmstadt, Germany) according to the manufacturer’s instructions. Additional information is summarized in Supplementary Table 1.

### Cell line treatments

BGA002 PNA was produced by Biogenera SpA (Bologna, Italy). The PNA-peptide was prepared by the chemistry department and, after purification and dilution, delivered to the biology department. The PNA was freshly produced and used or stored at 4 °C. PNA preparation was conducted according to methods described in previous studies [14–16]. The 13-cis retinoic acid (13cis-RA) was purchased from Sigma Aldrich and diluted in ethanol. Cell line expansion was conducted in RPMI-1640, with 10%FBS. Neuroblastoma adherent cells were detached using PBS-EDTA, which was followed by washing and counting with nigrosin using a Burker’s chamber. Treatment with BGA002, 13cis-RA and the combination (BGA002 + 13cis-RA) were conducted in OPTI-MEM medium. For the RNA extraction experiment, 5 × 10^4^ cells were plated in a 24-well, flat-bottom plate. For the cell viability assay, 5 × 10^3^ cells were plated in a 96-well, flat-bottom plate. Neuroblastoma cell lines were treated with increasing concentrations ranging from 0.6 μM to 10 μM. After 6 hours of treatment, up to 4% of FBS was added to the cells.

### Quantitative real-time PCR

RNA extraction, retro-transcription, and real-time PCR were performed as previously described [16]. Primers used in this study are listed in Supplementary Table 2.

### Cell viability assay and Western blot analysis

Cell viability assays were performed as previously described [16]. Cell viability assays were performed as previously described [16] using Cell Titer Glo Viability Assay (®) kit Promega. A Western blot analysis was conducted using standard methods [19]. Briefly, cells were lysed in radioimmunoprecipitation assay lysis buffer (containing 20 mM Tris-HCl (pH 7.5), 150 mM NaCl, 1 mM Na_2_EDTA, 1 mM EGTA, 1% NP-40, 1% sodium deoxycholate, 2.5 mM sodium pyrophosphate, 1 mM b-glycerophosphate, 1 mM Na_3_VO_4_, and 0.1% SDS) supplemented with Protease and Phosphatase Inhibitor Cocktail (Thermo Fisher Scientific Inc., Rockford, IL, USA). After sonication, cells were centrifuged at 15,000×g at 4 °C for 20 minutes and protein fractions were collected. A total of 30 μg of proteins were separated via SDS-PAGE using Criterion TGX polyacrylamide gels (Bio-Rad, Hercules, CA, USA) and blotted onto a nitrocellulose membrane (Bio-Rad, Hercules, CA, USA). Proteins were detected using Amersham ECL Prime Western Blotting Detection Reagent (GEHealthcare, Little Chalfont, Buckinghamshire, England). The ChemiDoc-It2 Imaging System and Vision Works LS Software (UVP, LLC, Upland, CA, USA) were used for the analysis. Bands were uncovered by the Amersham ECL detection system. The expression of specific proteins was assessed using the following antibodies: anti-N-Myc 1:800; anti-Phospho-Akt (Ser473) (#4060) 1:1000; anti-Akt (#9272) 1:1000; anti-Phospho-p70 S6 Kinase (Thr389) (#9206) 1:1000; anti-p70 S6 Kinase (#9202) 1:1000; anti-Phospho-S6 Ribosomal Protein (Ser235/236) (#4858) 1:1000; anti-S6 Ribosomal Protein (#2217) 1:1000; anti-Phospho-4E-BP1 (Thr37/46) (#2855) 1:1000; anti-4E-BP1 (#9452) 1:1000; and anti-glyceraldehyde 3-phosphate dehydrogenase (GAPDH) (#5174) 1:1000. All antibodies, except N-Myc, were obtained from Cell Signaling Technology (Danvers, MA, USA). N-Myc (sc-53,993) was from Santa Cruz Biotechnology (Dallas, TX, USA).

### Apoptosis analysis

The Kelly, LAN-5, SK-N-BE(2)-C, and TET-21N cell lines were treated as described above. Cells were stained with an Annexin V/FLUOS Staining Kit (F. Hoffmann-La Roche AG, Basel, Switzerland) according to the manufacturer’s instructions. The cell samples were analyzed via CytoFLEX flow cytometer (Beckman Coulter Inc., Brea, CA, USA). The results were analyzed using FlowJo software (Tree Star Inc. Ashland, OR, USA).

### Transmission electron microscopy

Kelly and LAN-5 cells were seeded at a density of 20,000 cell/cm^2^ in 6-well culture plates. Twenty-four hours after seeding, cells were treated with NaCl 0.9%, BGA002 2.5 μM, 13cis-RA 2.5 μM, and BGA002 2.5 μM + 13cis-RA 2.5 μM in FBS-free culture medium. After 6 hours, up to 4% of FBS was added and treatment proceeded for up to 48 hours at 37 °C with 5% CO_2_. Before fixing, cells were treated overnight with 60 μM chloroquine.

At the end of the experiments, the cells were fixed in 2.5% buffered glutaraldehyde directly in 6-well culture plates for 20 minutes at room temperature. They were then detached with a scraper, collected in tubes, pelleted and kept at 4 °C overnight. After washing in phosphate buffer, the cells were post-fixed in 1% buffered osmium tetroxide for 1 hour at 4 °C, washed and dehydrated through graded ethanol followed by embedding in Araldite resin. Samples were sectioned with a ultramicrotome and the ultra-thin sections were collected on grids and counterstained with uranyl acetate and lead citrate. Samples were examined using a Philips CM100 Transmission Electron Microscope (FEI Company, ThermoFisher, Waltham, MA, USA). Digital images were obtained using an Olympus camera (Tokyo, Japan).

### Morphological analysis of differentiation

The Kelly, LAN-5, SK-N-BE(2)-C, and SH-SY5Y cells were seeded in OPTI-MEM 4% FBS for 24 hours in 6-well plates (Thermo-scientific). Cell number to plate ratio was calculated to avoid confluence. After 24 hours, the cells were treated with 1.25 μM of BGA002, 1.25 μM of 13cis-RA, and 1.25 μM each and BGA002 + 13cis-RA. The treatment was repeated every 48 hours with fresh medium. Images were acquired every 48 hours using an Eclipse TE2000-S microscope (Nikon, Tokyo, Japan). Cells were kept for an additional 9 days (while continuing to change the medium every 48 hours) until day 18. After 12 hours, 9- and 18-day cells were lysed and RNA was extracted as described above. We measured the extension of neurites using Simple Neurite Tracer plug-in in Image J software (National Institutes of Health, Bethesda, MD, USA). Neural network analysis is described in detail in the supplementary data.

### Wound healing assay

The Kelly, LAN-5, SK-N-BE(2)-C, LAN-1, SH-SY5Y, and TET-21N cell lines were seeded in OPTI-MEM 4% FBS to reach confluence after 24 hours in 12-well plates (Thermo-scientific). The day after seeding, a scratch on the cell monolayer was made using a 200 μL tip. Cells were then treated with BGA002 (at 1.25 and 2.5 μM), 13cis-RA (at 1.25 and 2.5 μM) and BGA002 + 13cis-RA (at 1.25 each and 2.5 μM each). From the time of the treatment (day 0) the cells were maintained in culture for up to 72 hours (photos were acquired every 24 hours using an Eclipse TE2000-S microscope (Nikon, Tokyo, Japan)). Cells were then lysed and their RNA extracted as described above. Images were analyzed using the Wound Healing Tool plugin in ImageJ1.46r (NIH). The percentage of the area occupied by the cells was calculated with respect to day 0.

### Lysosome area measurement

The LAN-5 and Kelly cell lines were seeded in a Nunc Lab-Tek Flask on Slide for live staining. Treatment was administered 48 hours before acquisition. A Lyso-Tracker was added and the cells were incubated for 45 minutes at 37 °C at 5% CO_2_. For each condition, z-stacks (at a 200 nm interplane distance) were acquired using a Nikon Ti2-E microscope (Nikon, Tokyo, Japan). Images were elaborated using the Fiji plugin in ImageJ software. Z-stacks containing lysosomes were selected using Image>Stack>Tool>Slice Keeper. Once selected, all images were binarized using Process>Binary>Make Binary with the Yen method. Lysosomes were then analyzed using Analyze>Analyze Particles, with the lower value size set to 0.1 um^2^.

### Neuroblastoma luminescent cells and the xenograft ectopic neuroblastoma mouse model

CHP-134*-luc* was prepared as described previously [16]. CHP-134-Luc cell line was chosen because shows a better engraftment ratio in comparison with previous cell line tested for such as Kelly-Luc previously used. All experiments with mice were approved by the Scientific Ethical Committee of Bologna University (protocol no. 07/73/2013 and 564/2018-PR). Six-week-old mice (NOD/SCID CB17; both sexes) were inoculated with CHP-134*-luc* (10 × 10^6^ cells for each animal) in the dorso–posterio–lateral position. Prior to injection, mice were sedated with isoflurane. Luminescence was used to monitor the growth of tumors (D-Luciferin was administered via intraperitoneal injection, and luminescence was monitored using the UviTec Imaging System (Cleaver Scientific, Ltd., Rugby, UK). Treatment administration began after a predefined starting point during bioluminescent acquisition and was conducted daily for 28 days with an injection of 100 μL of vehicle, 10 mg/kg/day of BGA002, 10 mg/kg/day of 13cis-RA, and 10 mg/kg/day each of BGA002 and 13cis-RA. Vehicle and BGA002 were administered via subcutaneous injection while 13cis-RA was given via intraperitoneal injection. Animals were monitored until they reached the endpoint (10 mm linear tumor or 60 days post treatment). Tumor size and volume was calculated using a caliper. After reaching the endpoint, the mice were sacrificed. The tumors were removed, measured, weighed, and fixed in 4% formalin. Immunohistochemistry was conducted as previously described [16].

### Statistical analysis

Statistical analysis was performed with the Prism software version 6 (GraphPad) or with R software version 3.5 or Python software version 3.7. The different analyses and tests were specifically designed for each experiment.

### Data availability

The data generated in this study are available within the article and its supplementary data files. Expression profile data analyzed in this study were obtained from Gene Expression Omnibus (GEO) at GSE9169, GSE80151 and GSE80153, from Array Express at E-MTAB-1781, from the TARGET repository at the official website, from Cancer Cell Line Encyclopedia.

## Results

### BGA002 in combination with RA cooperates to inhibit *MYCN* activity

**Fig. 1:**
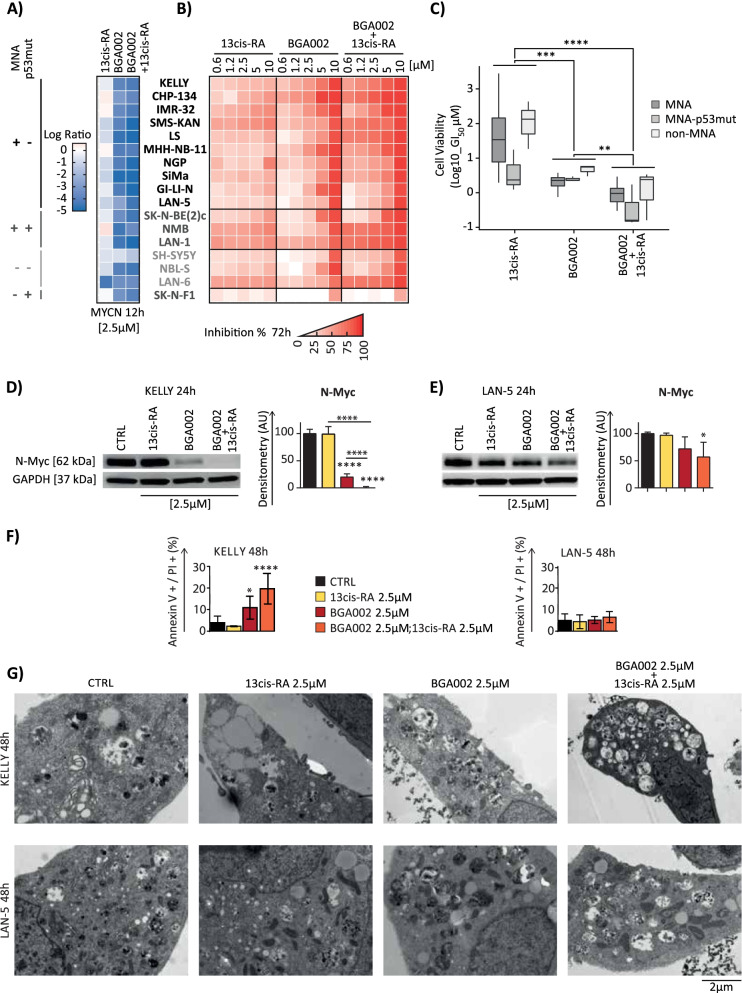
BGA002 in combination with RA cooperates to inhibit *MYCN* activity in neuroblastoma. **A-B** cell lines are listed in the middle and grouped according to *MYCN* amplification status and/or p53 mutation status (MNA cell-lines: KELLY, CHP-134, IMR-32, SMS-KAN, LS, MHH-NB-11, NGP, SIMa, GI-LI-N; MNA-p53mut: SK-N-B [2] c, NMB, LAN-1; single copy: NBL-S, LAN-6, SH-SY5Y; single copy and p53mut: SK-N-F1). **A** a heatmap representing in vitro efficacy for mRNA inhibition after 12-hour treatment at 2.5 μM. The color scale represents the Log_2_ fold-change of *MYCN* mRNA with respect to untreated cells (*n* = 3 experiments for each cell line). **B** a heatmap representing in vitro efficacy for cell viability inhibition after 72 hours of treatment at different doses. The color scale represents the percentage of inhibition normalized over the control (*n* = 3 experiments). **C** decrement in viability by log10 GI_50_ and grouped by *MYCN* amplification and/or p53 mutation status. In the box plot, the median is indicated as the middle line, the outer edges of the box represent the first and third quartiles, and whiskers represent samples within the 1.5 interquartile ranges. Wilcoxon matched-pair test. **D-E** representative Western blot analysis after 48 hours of treatment (*n* = 3 experiments for each cell line). Left, representative staining for N-Myc (top) and associated GAPDH staining (bottom). N-Myc quantification normalized over the GAPDH is presented on the right. The bars represent the mean of 3 experiments, and the whiskers represent the standard deviation. **D** Western blot analysis for the Kelly cell line (*MYCN* amplified, p53 wild type). **E** Western blot analysis for the LAN-5 cell line (*MYCN* amplified, p53 wild type). **F** apoptosis measurement after 48 hours of treatment for the Kelly (left) and LAN-5 (right) cell lines (*n* = 3 experiments for each cell line). Bar plots represent the percentage of cells stained by AnnexinV^+^/PI^+^. The bars represent the mean, and the whiskers are the standard deviation. **G** transmission electromicrographs of cell lines treated for 48 hours. Kelly (top) and LAN-5 (down) representative images for each condition are presented (*n* = 2 experiments for each cell line). CTRL, control. Where indicated in the figure: *, *p* < 0.05; **, *p* < 0.01; ***, *p* < 0.001; ****, *p* < 0.0001

In a previous article, we demonstrated that BGA002 was able to block *MYCN* expression in neuroblastoma cell lines [16]. Therefore, here we investigated how the combined treatment of BGA002 and RA would affect *MYCN* expression in a broad panel of neuroblastoma cell lines (17 cell lines, which recapitulated the neuroblastoma landscape: MNA cell lines (*n* = 10), MNA p53-mutated (*n* = 3), non-MNA (*n* = 3), and non-MNA p53-mutated (*n* = 1)). Treatment with RA alone achieved poor inhibition of *MYCN* mRNA expression. Treatment with BGA002 showed a marked reduction in all neuroblastoma cell lines, and combined treatment with RA further strengthened *MYCN* inhibition (Fig. 1A) in a dose-dependent manner (Supplementary Fig. S1A). We also tested cell-viability inhibition after treatment in the same neuroblastoma cell line panel. While RA alone showed a modest effect, BGA002 strongly inhibited cell viability in all cell lines in a dose-dependent manner (Fig. 1B and Supplementary Fig. S1B), and the combined treatment led to a significantly stronger effect as demonstrated by a lower GI_50_ (Fig. 1B-C). Moreover, the combined treatment of BGA002 and RA was found to be synergic (Supplementary Fig. S2A-D). We also verified that the *MYCN* mRNA inhibition translated to a decrease in proteins and that BGA002-RA treatment led to a stronger N-Myc decrease in three *MYCN*-amplified neuroblastoma (MNA-NB) cell lines (Fig. 1D-E and Supplementary Fig. S3A). Furthermore, RA failed to induce apoptosis in the neuroblastoma cell lines, while BGA002 alone induced apoptosis and the combined treatment with RA reinforced this effect, especially in MNA-NB Kelly cells (Fig. 1F and Supplementary Fig. S3B-C).

Treatment with BGA002-RA strengthened BGA002-induced mitochondria alteration in Kelly cells, as demonstrated by electronic microscope ultrastructural analysis, size, and perimeter reduction. RA alone had no activity (Fig. 1G). The ultrastructural analysis also showed that BGA002-RA led to the consistent appearance of macrovacuoles in MNA-NB cells (Kelly) (Fig. 1G). Interestingly, we found a lower apoptotic effect in other MNA-NB cells (LAN-5 and SK-N-BE(2)-C) (Fig. 1F and Supplementary Fig. S3B), while Tet-21N cells did not undergo apoptosis (Supplementary Fig. S3C). We also noticed that the mitochondria in MNA-NB cells (LAN-5) with a lower apoptosis effect after BGA002-RA treatment were similar to untreated cells (Fig. 1G). Confocal image analysis also showed a dramatic reduction in mitochondrial volume in Kelly cells, however, the same extent of damage in LAN-5 cells was not observed (Supplementary Figs. S4–5 and Supplementary Fig. S6A-B).

### BGA002-RA treatment induces differentiation in MNA-NB cells

Undifferentiated neuroblastomas are considered high risk and are associated with poor survival outcomes. Therefore, we separated neuroblastoma patient expression profiles into 2 clusters for differentiation status using a differentiation signature (1557 genes, based on gene ontology pathways) (Supplementary Fig. S7A-D). We also investigated the transcription regulation of differentiation and used a dataset of neuroblastoma cell lines treated with RA to identify putative transcription factors involved in differentiation. We used this list of transcription factors to identify regulons that were differentially active in two different neuroblastoma cohorts (Supplementary Fig. S8A-B). We identified 3 clusters of neuroblastoma patients, according to the selected regulon activity implied in differentiation, which showed differential survival (Supplementary Fig. S8C-D).

**Fig. 2:**
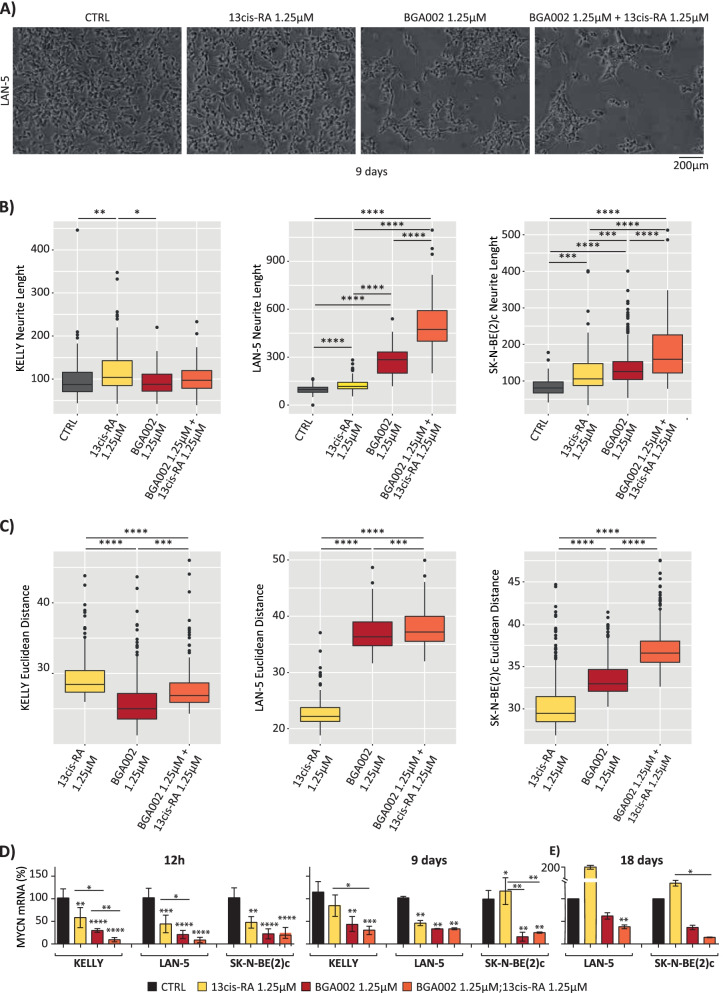
BGA002-RA treatment induces differentiation in MNA-NB cell lines. **A** optical microscopy image for the LAN-5 (*MYCN* amplified, p53 wild type) cell line treated for 9 days (from left to right, untreated, 1.25 μM RA, 1.25 μM BGA002, 1.25 μM each BGA002 + RA). Two biological replicates were used for the experiment. **B** boxplots represent the length of the neurite in MNA-NB cell lines (Kelly (*MYCN* amplified, p53 wild type), LAN-5 (*MYCN* amplified, p53 wild type), SK-N-BE(2)-C (*MYCN* amplified, p53 mutated)) after 9 days of treatment (CTRL: medium alone, RA: retinoic acid 1.25 μM, BGA002: BGA002 1.25 μM, BGA002 + RA: BGA002 1.25 μM and retinoic acid 1.25 μM). Each dot represents the measurement for a single neurite, the middle line represents the median, the outer edges of the box indicate the first and the third quartiles, and the whiskers specify samples within 1.5 times the interquartile range. The graph represents the results of two pooled experiments. Statistic: Wilcoxon matched-pair test. **C** box plots represent the Euclidean distance in MNA-NB cell lines (Kelly, LAN-5, SK-N-BE(2)-C) after 9 days of treatment. The Euclidean distance was calculated between the CTRL (medium control) and each treatment condition (RA: retinoic acid 1.25 μM, BGA002: BGA002 1.25 μM, BGA002 + RA: BGA002 1.25 μM and retinoic acid 1.25 μM). Each dot represents the measurement for a single image, the middle line represents the median, while the outer edges of the box indicate the first and third quartiles. The whiskers specify samples within 1.5 times the interquartile range. The graph represents the results of two pooled experiments. Statistic: Wilcoxon matched-pair test. Where not indicated, the *p*-value was not significant. **D-E** *MYCN* mRNA expression normalized over the control (*n* = 3 experiments for each cell line). The bar represents the mean, and the whiskers are the standard deviation. **D** left panel: *MYCN* mRNA expression in Kelly, LAN-5 and SK-N-BE(2)-C cell lines after 12 hours (left) and 9 days (right) post treatment. Right panel: *MYCN* mRNA expression in LAN-5 cells after 18-days post treatment. CTRL, control. Where indicated in the figure: *, *p* < 0.05; **, *p* < 0.01; ***, *p* < 0.001; ****, *p* < 0.0001

As RA treatment is known to induce neuron differentiation, and *MYCN* inhibition is necessary to fully achieve differentiation, we tested whether BGA002-RA could lead to MNA-NB cell differentiation. Specifically, we used SH-SY5Y cells as a control for differentiation (Supplementary Fig. S9A-B). We treated the MNA-NB cell line LAN-5 with BGA002 and/or RA (2.5 μM) for 9 days and obtained optical microscope images at different time points (Supplementary Fig. S9C-E). We also tested the differentiation activity of a lower concentration of BGA002 and/or RA (1.25 μM) in MNA-NB cell lines (LAN-5, SK-N-BE(2)-c, and Kelly) for 9 days. Microscopic images showed that RA alone was sufficient to induce differentiation in SH-SY5Y but not in MNA-NB cells (Fig. 2A, Supplementary Fig. S9A and Supplementary Fig. S10A-B). Conversely, we observed an increase in neurite length with BGA002 treatment only in MNA-NB cells (Fig. 2B and Supplementary Fig. S10C). The combined treatment of BGA002-RA showed a significant increase in neurite length (Fig. 2B and Supplementary Fig. S10C).

To measure the differences between the different conditions, we trained a convolutional neuron network as a feature extractor. On the new feature vectors, we calculated the Euclidean distance between the control condition and the other conditions (Supplementary Fig. S11A-D). This complementary approach confirmed that MNA-NB cells treated with BGA002 and BGA002-RA were different from untreated cells, while RA-treated MNA-NB cells were similar to untreated cells (Fig. 2C and Supplementary Fig. S11E). The Kelly MNA-NB cell line, which exhibited high levels of apoptosis following BGA002 or BGA002-RA treatment, failed to undergo differentiation. Remarkably, an evaluation of the differentiated phenotype of BGA002-RA treated LAN-5 cells performed after an additional 9 days without treatment showed a persistence of the differentiation status (Supplementary Fig. S12A). In LAN-5 cells, *MYCN* mRNA expression also resulted in inhibition after 9 days of treatment (Fig. 2D and Supplementary Fig. S12B), and this inhibition persisted after an additional 9 days without treatment (Fig. 2D). These data were further confirmed by confocal microscope analysis, performed using synapsin-1 as marker for differentiation in LAN-5 MNA-NB cells. After 9 days, neuron-like structures resulted well established only in cells treated with BGA002-RA, in which the cytoplasm showed long, ramified protrusions and the cells clustered together (Fig. S13A). Synapsin-1 quantification in these cells treated with BGA002-RA showed a higher value compared to untreated cells or to single treatments (Fig. S13B), reinforcing the data on the differentiation involvement.

### BGA002-RA treatment rebalances cellular retinoic acid-binding protein 1/2 in RA-resistant neuroblastoma

**Fig. 3:**
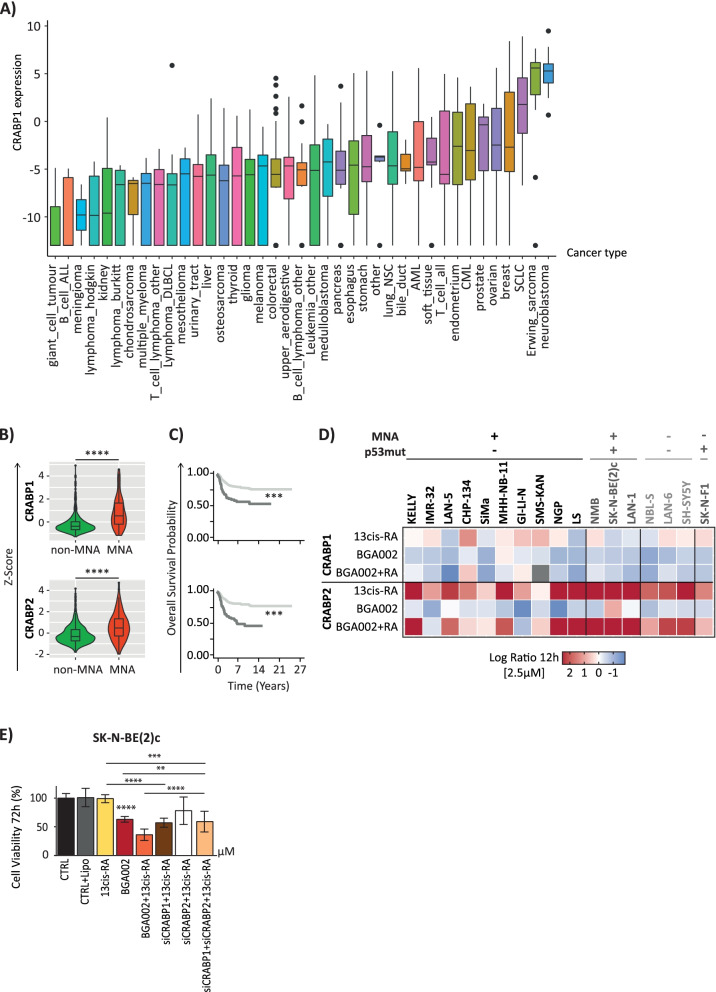
CRABP1/CRABP2 rebalances in RA-resistant neuroblastoma after BGA002-RA treatment. **A** boxplot represents the mRNA expression of CRABP1. Each boxplot represents the mRNA expression for a cancer type as listed in the Cancer Cell Line Encyclopedia. Each dot represents the mRNA expression for a single cell line, the middle line represents the median, the outer edges of the box indicate the first and the third quartiles, and the whiskers specify samples within 1.5 times the interquartile range. **B** mRNA expression of CRABP1 and CRABP2 in an neuroblastoma dataset (E-MTAB-1781). The violin plot represents normalized (z-score) mRNA expression for non-MNA and MNA patients. Each dot represents an individual sample; the middle line indicates the median. Statistic: Wilcoxon matched-pair test. **C** Kaplan–Meier plots for the probability of overall survival over time for neuroblastoma patients (E-MTAB-1781). The dark grey line indicates patients with normalized gene expressions higher than 1 (z-score > 1). The *p*-value is indicated in the middle (Log-rank test). **D** a heatmap of the gene expression variation after 12 hours of treatment in neuroblastoma cell lines. Columns represent cell lines (grouped according *MYCN* amplification and p53 mutation status), rows represent CRABP1 and CRABP2, color scale represents the log_2_ fold change over the control (untreated). The grey color indicates unexpressed genes. (MNA cell-lines: KELLY, CHP-134, IMR-32, SMS-KAN, LS, MHH-NB-11, NGP, SIMa, GI-LI-N; MNA-p53mut: SK-N-B [2] c, NMB, LAN-1; single copy: NBL-S, LAN-6, SH-SY5Y; single copy and p53mut: SK-N-F1) **E** Cell viability after 72 hours of treatment in SK-N-BE [2] c cells (*n* = 3 different biological replicates). SK-N-BE [2] c cells is *MYCN* amplified, p53 mutated. Where indicated in the figure: *, *p* < 0.05; **, *p* < 0.01; ***, *p* < 0.001; ****, *p* < 0.0001

**Fig. 4:**
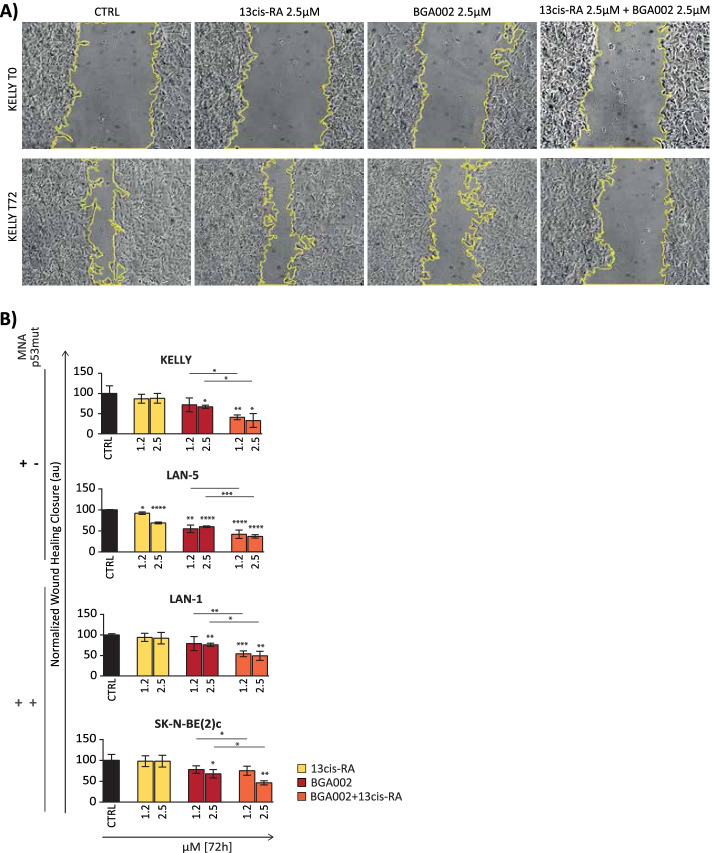
BGA002-RA treatment affects the migration capacity of MNA-NB. **A** optical microscopy image of the Kelly cell line treated at time zero (above the line) or after 72 hours (below the line). Exemplary images from 1 out of 3 experiments. The scratched zone is highlighted with yellow contouring. **B** bar plot represents wound-healing closure normalized over the control after 72 hours for 4 different MNA-NB cell lines (*n* = 3 different biological replicates). The middle line is the mean and the whiskers are the standard deviation. CTRL, control. Where indicated in the figure: *, *p* < 0.05; **, *p* < 0.01; ***, *p* < 0.001; ****, *p* < 0.0001

The high expression level of cellular retinoic acid-binding protein (CRABP)1 and the low expression level of CRABP2 play a role in resistance to RA treatment in breast and pancreatic tumors; however, currently, no specific studies have investigated their roles in neuroblastoma [20–22]. In this study, we found that neuroblastoma had the highest CRABP1 expression (Fig. 3A). Moreover, we found that CRABP1 had a significantly higher expression in MNA-NB patients (Figs. 3B and S3B), and a higher expression was linked to a worse prognosis (Fig. 3C). It is known that CRABP1 sequesters RA in the cytoplasm. Its elevated expression in neuroblastoma could cause RA resistance by limiting RA access to the nucleus, which is mediated by binding to CRABP2 [23, 24]. Therefore, we tested how specific *MYCN* inhibition by BGA002 and RA treatment affected CRABP1/2 expression in neuroblastoma cell lines. Interestingly, only the combined BGA002-RA treatment induced a concomitant downregulation of CRABP1 and upregulation of CRABP2 expression (Fig. 3D). When used as single agents, RA upregulated CRABP1 and CRABP2 while BGA002 downregulated CRABP1 and CRABP2 (Fig. 3D). We tested the effect of CRABP1 and CRABP2 inhibition in MNA and p53mut neuroblastoma cell lines (SK-N-BE(2)-C), which are known to be resistant to RA. Treatment with RA alone showed no prominent effect on viability, while the addition of siRNA against CRABP1 overcame RA resistance (Fig. 3E). Interestingly, the addition of siRNA against CRABP2 blocked CRABP1-restored RA susceptibility (Figs. 3E and 4).

### BGA002-RA treatment inhibits the migration capacity of MNA-NB

*MYCN* expression levels correlate with metastatic behavior, which leads to decreasing adhesion and increasing motility, invasion, and matrix degradation [25]. On the one hand, N-Myc downregulates integrins (α1, β1) and E-cadherin. On the other hand, it leads to increases in focal adhesion kinase and the expression of metalloproteases [26–30]. Here we found that pathways related to cell adhesion were negatively enriched in the differentially expressed genes between MNA and non-MNA-NBs in two different datasets (Supplementary Fig. S14). Furthermore, these pathways were enriched in the genes that were inversely correlated with *MYCN* in the two datasets (Supplementary Fig. S14). Therefore, we investigated how BGA002-RA affected invasion and migration through a wound-healing assay in MNA-NB (Supplementary Fig. S15A). We observed a modest effect following RA treatment with 3 different concentrations and only at late time points (48 or 72 hours) (Supplementary Fig. S15A and B). In contrast, we found the inhibition of migration capacity after treatment with BGA002 (2.5 μM) at the earliest time point (24 hours), which then increased at later time points (Supplementary Fig. S15A and B). The combined treatment with BGA002-RA further increased the effect of migration inhibition. Specifically, we observed an inhibitory effect at the earliest time-point, which increased over time, with the inhibition also observable at lower doses (0.6 and 1.25 μM) (Supplementary Fig. S15B). At the molecular level, we investigated whether genes involved in migration were downregulated following *MYCN* inhibition [30–33] and found that BGA002 or BGA002-RA treatments were able to downregulate genes involved in MNA-NB migration. A substantial effect after RA treatment alone was not observed (Supplementary Fig. S16A).

### BGA002-RA treatment leads to mTOR complex inhibition in MNA-NB

In *MYCN*-related mice models, phosphatidylinositol 3-kinase (PI3K)/mTOR pathway inhibition is reported to destabilize N-Myc and be effective against tumors [34, 35], while other studies have reported that N-Myc could regulate the mTOR pathway in neuroblastoma [36, 37]. However, it has been shown that, in other tumors, RA is capable of inhibiting mTOR [38]. In the present study we found that neuroblastoma presented the highest level of mRNA expression of genes involved in the mTOR pathway (small cell lung cancer ranked second) and presented low promoter methylation (Supplementary Fig. S17A). Moreover, neuroblastoma and small cell lung cancer clustered together for mTOR gene expression (Supplementary Fig. S17B-D), which could be related to the fact that these two highly aggressive tumors derive from peripheral nervous system cells.

**Fig. 5:**
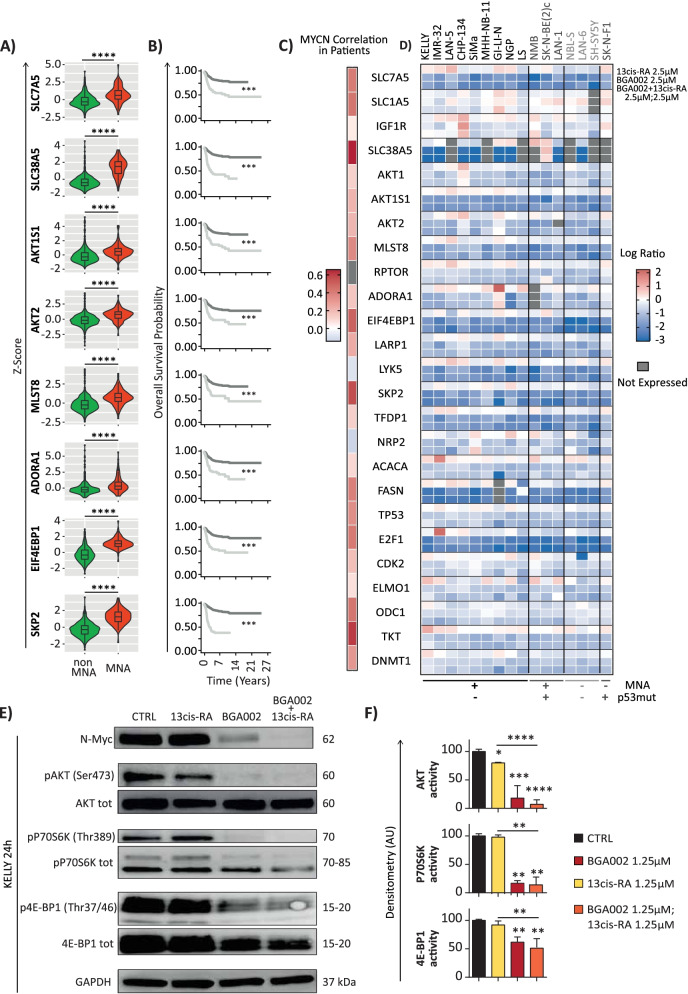
BGA002-RA treatment leads to mTOR complex inhibition in MNA-NB. **A** mRNA expression of genes involved in the mTOR pathway in an neuroblastoma dataset (E-MTAB-1781). The violin plot represents normalized (z-score) mRNA expression for non-MNA and MNA patients. Each dot represents an individual sample; the middle line indicates the median. Statistic: Wilcoxon matched-paired test. ****p ≤ 0.0001. **B** Kaplan–Meier plots for the probability of overall survival over time for neuroblastoma patients (E-MTAB-1781). The dark gray line indicates patients with normalized gene expressions higher than 1 (z-score > 1). The *p*-value is indicated in the middle (Log-rank test). **C-D** The gene names listed in the middle are referring to both panels. (MNA cell-lines: KELLY, CHP-134, IMR-32, SMS-KAN, LS, MHH-NB-11, NGP, SIMa, GI-LI-N; MNA-p53mut: SK-N-B [2] c, NMB, LAN-1; single copy: NBL-S, LAN-6, SH-SY5Y; single copy and p53mut: SK-N-F1). **C** a heatmap representing Pearson correlation coefficients for mTOR pathway genes. **D **Heatmap of the gene expression variation after 12 hours of treatment in neuroblastoma cell lines. Columns represent cell lines (grouped according *MYCN* amplification and p53 mutation status), rows represent genes belonging to the mTOR pathway, and the color scale represents the log_2_ fold change over the control (untreated). The gray color indicates unexpressed genes. **E** mTOR pathway activity measured through Western blot in the Kelly cell line (*MYCN* amplified, p53 wild type) after 24 hours of treatment (representative image is 1 out 2 biological replicates). **F** mTOR pathway activity quantification normalized over the control (*n* = 2 experiments, ** p-value ≤0.0001, ANOVA). CTRL, control. Where indicated in the figure: *, *p* < 0.05; **, *p* < 0.01; ***, *p* < 0.001; ****, *p* < 0.0001

Neuroblastoma cell lines presented high expressions for different genes of the mTOR pathway (Supplementary Fig. S18A) and a higher expression was found in MNA versus non-MNA patients (Fig. 5A). Remarkably, these genes were also significantly predictive for overall survival (Fig. 5B), and strongly correlated with *MYCN* expression (Fig. 5C). Interestingly, MNA-NB cell lines showed a higher GI_50_ when treated with mTOR inhibitors (Supplementary Fig. S19A-B), and a ChIP-seq public data analysis showed that N-Myc directly regulated different mTOR pathway genes (Supplementary Fig. S19C-D). Therefore, we tested whether BGA002-RA could inhibit the mTOR pathway in MNA-NB. We found that BGA002 or BGA002-RA strongly inhibited the expression of genes involved in the mTOR pathway, while RA alone failed to downregulate their expression (Fig. 5D). We also evaluated mTOR pathway activity through protein phosphorylation. The results showed a reduction in protein kinase B (AKT), p70S6K, and 4E-BP1 phosphorylation after treatment with BGA002, which was strengthened by BGA002-RA; thus, demonstrating mTOR pathway inhibition in MNA-NB (Fig. 5E-F).

We noticed that neuroblastoma patients with high mTOR pathway activity had significantly worse survival outcomes (Supplementary Fig. S20A). Therefore, we combined mTOR activity, differentiation score, and *MYCN* status into a single score to predict the survival of neuroblastoma patients. Using receiver operating characteristic curve analysis, we tested the predictive ability and accuracy of our model. The combined score showed a high predictive ability at 1 year (area under the curve = 0.914) and satisfactory predictive ability at 3 and 5 years (Supplementary Fig. S20B). The combined score was superior to the single components (Supplementary Fig. S20C) and had superior accuracy in comparison to commonly used clinical characteristics (Supplementary Fig. S20D).

### BGA002-RA treatment leads to autophagy reactivation

**Fig. 6:**
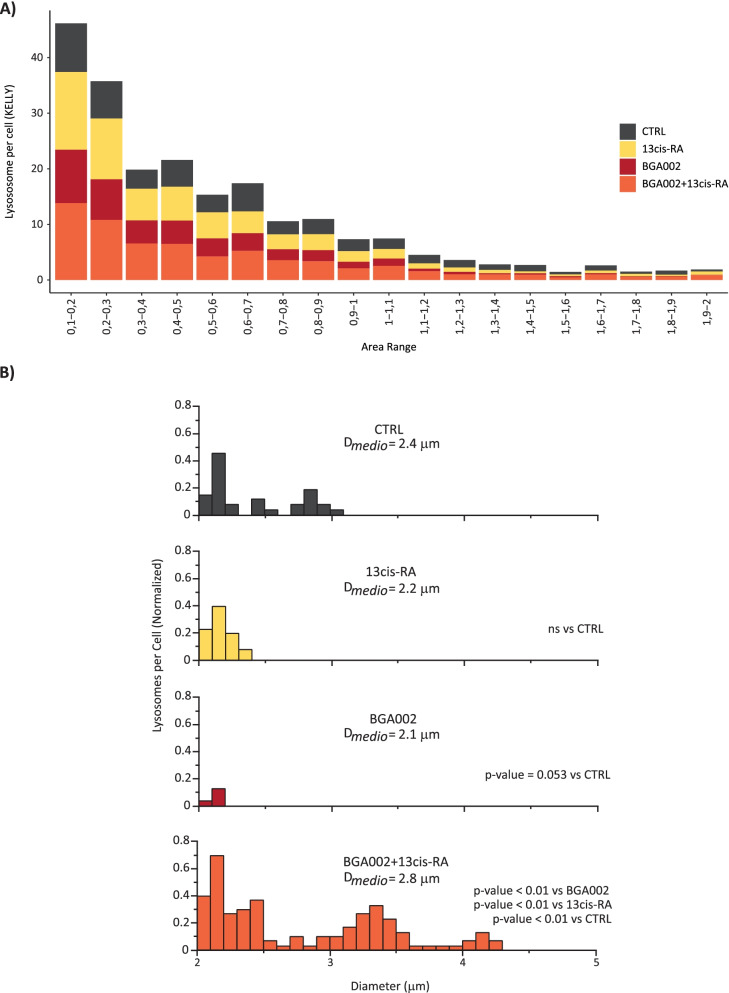
BGA002-RA treatment leads to autophagy reactivation in MNA-NB. **A** stacked bar-plot with the vertical axis representing the number of lysosomes per cell after 24 hours of treatment in the Kelly MNA-NB cell line (*MYCN* amplified, p53 wild type). The horizontal axis is the area range of the lysosomes. Colors represents treatment (control, 13-cis RA, BGA002, BGA002 + RA). **B** single bar plot for each treatment group is represented. As described previously, vertical axis represents the number of lysosomes and horizontal axis represents the area range of lysosomes. Only lysosomes considered with high dimension (> 2 μm) are represented in each graphs. Kelly treated with combination of BGA002 and 13-cis RA shows lysosomes with the highest diameters compared with other treatment group (two-sided unpaired test-T *p* < 0.01). BGA002 alone show only small differences compared with control (*p* = 0.053) and RA alone shows no differences

Metabolic reprogramming is a cancer hallmark [39] and the mTORC1 complex plays an important role in metabolic control while suppressing autophagy [40]. In our investigation, we found that pathways related to autophagy were negatively enriched in differentially expressed genes in MNA with respect to non-MNA in two different neuroblastoma datasets (Supplementary Fig. S21A-C). In addition, *MYCN* silencing in an inducible *MYCN* model exhibited autophagy signature re-expression in concomitant mTOR downregulation datasets (Supplementary Fig. S22A-B). As we found mTOR pathway downregulation following BGA002-RA treatment, we evaluated whether this event resulted in autophagy reactivation. Treatment with BGA002-RA showed an increase in lysosomes after the treatment in MNA-NB cell lines (Fig. 6A and Supplementary Fig. S23A-B), and in particular higher diameter lysosomes (> 2 μm) resulted more numerous in this condition respect the single treatments (Fig. 6B). Electron microscopy analysis also uncovered the appearance of a large number of macrovacuoles after BGA002-RA treatment in MNA-NB cells (Fig. 1G).

### BGA002-RA shows an in vivo anti-tumor effect against MNA-NB

**Fig. 7:**
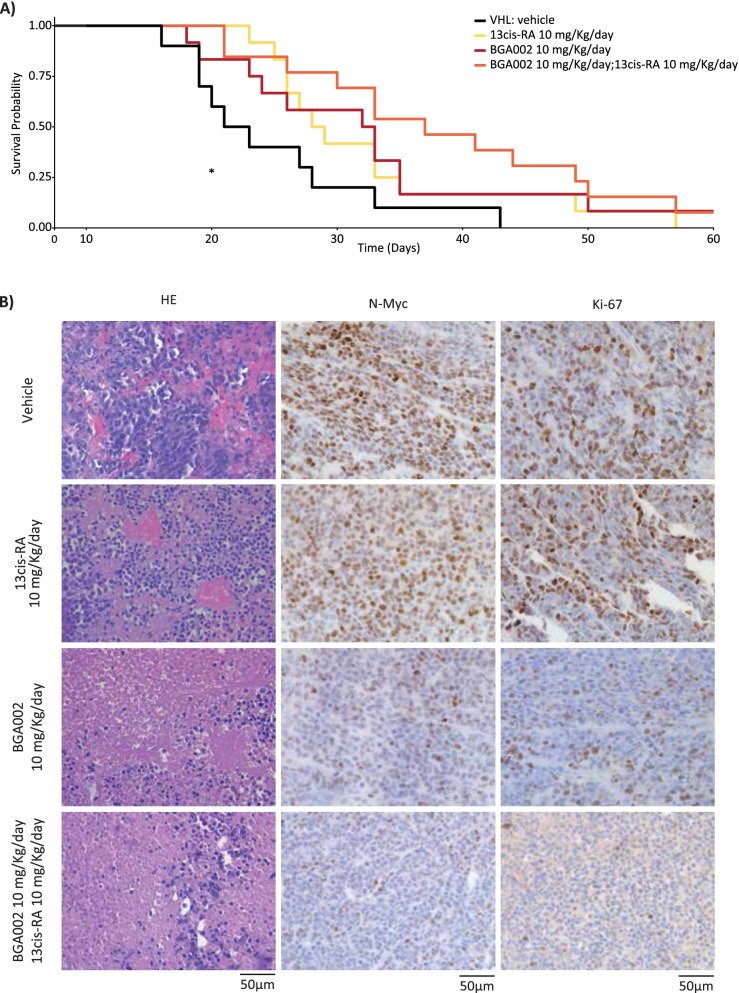
BGA002-RA inhibits vascularization of MNA-NB and improves survival in mice. **A** Kaplan-Meyer plot for the probability of event-free survival over time for CHP-134-*luc* (*MYCN* amplified, p53 wild type) xenograft mice treated with: vehicle (black line, *n* = 10), 13-cis RA 10 mg/kg/day (yellow line, *n* = 12), BGA002 10 mg/kg/day (red line, *n* = 12), 13-cis RA and BGA002 10 mg/kg/day (orange line, *n* = 13). In the middle of the plot is the associated p-value (log-rank test) *, *p* < 0.05. **B** immunohistochemistry analysis of neuroblastoma mice untreated (first column), treated with 13-cis RA 10 mg/kg/day (second column), treated with BGA002 10 mg/kg/day (third column), 13-cis RA and BGA002 10 mg/kg/day (fourth column). The first row shows staining with hematoxylin and eosin (H&E), the second row is the N-Myc antibody, and the third row Ki67 staining

We evaluated the anti-tumor capacity of systemic treatment with BGA002-RA in comparison to the vehicle, or BGA002 or RA alone in an MNA-NB xenograft mouse model (CHP-134 cells). BGA002 treatment alone or RA alone had already demonstrated survival augmentation (Fig. 7A), and we found that combined BGA002-RA treatment also showed a significant increase in survival (Fig. 7A and Supplementary Fig. S24A), and a significant hazard ratio reduction in comparison to the vehicle (0.28, *p*-value = 0.004) (Supplementary Fig. S24B). BGA002-RA treatment also reduced tumor growth during treatment in comparison to the vehicle (Supplementary Fig. S24C).

We conducted a histological analysis of tumors 60 days after the end of treatment. Tumor vascularization was strongly present in vehicle and remained in RA treated mice; however, it was not present in BGA002 and BGA002-RA treated mice (Fig. 7B). Moreover, immunohistochemistry analysis showed that N-Myc protein expression in RA-treated tumors was similar to vehicle. While BGA002 treatment was already capable of reducing N-Myc protein, BGA002-RA treatment consistently strengthened this effect (Supplementary Fig. S25A-B). In addition, we found that the trend in results observed for N-Myc protein staining in tumors was similar to the results found with Ki67 staining (Fig. 7B).

## Discussion

While retinoic acid (RA) treatment has shown high efficacy in the treatment of acute promyelocytic leukemia, numerous clinical trials are exploring the efficacy for a wide range of human malignancies [20]. Moreover, RA treatment has also been shown to be beneficial in high-risk neuroblastoma for controlling minimal residual disease therapy [41]. However, approximately 50% of neuroblastoma patients have intrinsic or acquired resistance to RA treatment, particularly patients with MNA-NB [42, 43]. As RA treatment is less toxic than chemotherapy and is able to induce differentiation in malignant cells, there is intensive research to overcome these efficacy limitations [20]. In this respect, different studies have proposed chemical RA modification, different formulations, or the use of RA in combination with other treatments [44]. Nevertheless, the exact mechanism of acquired resistance to RA treatment is still debated.

Beyond neuroblastoma, *MYCN* amplification has also been found in different neoplasias, and the list of malignancies where it plays a role is expanding [45]. Targeting N-Myc has high potential due to its role in cancer development, its association with a poor prognosis, its wide control of expression, and its restricted expression at the embryonic stage. Here we have demonstrated that combined BGA002 and RA treatment was able to inhibit *MYCN* expression and cell viability in both MNA- and non-MNA-NB cell lines showing a synergistic effect. Furthermore, in MNA/p53mut neuroblastoma cell lines (which are, on average more resistant to treatment) this effect was even more relevant—as indicated by a lower EC_50_. BGA002-RA showed improved efficacy in inhibiting N-Myc protein expression and in inducing apoptosis in comparison with the single treatment. Previously, we showed that *MYCN* inhibition by BGA002 led to the reactivation of mitophagy and cell death via mitochondria damage due to reactive oxygen species increases [16]. In line with this, we have now found mitochondria alteration following BGA002-RA administration, which is associated with apoptosis in MNA-NB cells.

Neuroblastoma pathogenesis has also been associated with differentiation failure and, especially in MNA-NB, the persistence of cancer cells in an undifferentiated, embryonic-like state [46]. However, we found that, at low doses, BGA002-RA was able to induce differentiation in MNA-NB cells. Results showed that untreated or RA-alone treated MNA-NB cell lines failed to undergo differentiation. Conversely, *MYCN* inhibition by BGA002 was already capable of inducing neurite length increases, and we found a stronger effect after combined BGA002-RA treatment in MNA-NB cell lines. Hence, the block of *MYCN* by BGA002 reverted the differentiation resistance to RA in MNA-NB cells. In this context, BGA002-RA did not alter mitochondria in MNA-NB cells that underwent differentiation. Our analysis showed that *MYCN* inhibition remained 9 days after the end of treatment. Thus, there was a persistence of the *MYCN* inhibition and of the differentiated phenotype after suspension of BGA002-RA treatment in MNA-NB cells. Furthermore, here we have presented a new complementary approach to quantify neuroblastoma differentiation that is based on a convolutional neural network. This method is scalable and can be used to monitor differentiation in neuroblastoma cell lines with different drug combinations. A similar approach can also be used to monitor changes in cellular morphology in high-throughput screening.

With the aim to elucidate the mechanisms of RA resistance in neuroblastoma, we examined the balance between cellular retinoic acid-binding protein (CRABP)1 and CRABP2. Remarkably, our analysis uncovered that CRABP1 had a significantly higher expression in MNA-NB patients that was linked to a worse prognosis. As it is known that CRABP1 sequesters RA in the cytoplasm, its elevated expression in neuroblastoma could cause RA resistance by limiting RA access to the nucleus mediated by its binding to CRABP2 [23, 24]. In this context, it is of relevance our finding that only the combined BGA002-RA treatment induced a concomitant downregulation of CRABP1 and upregulation of CRABP2 expression, reverting the CRABP1/2 balance in neuroblastoma cells.

The ability to migrate and invade is a cancer hallmark, and cancer cells with an undifferentiated and mesenchymal phenotype are more prone to metastasize [47]. Indeed, MNA-NBs have a high metastatic capacity in different target sites in the body [48, 49]. Our results showed that blocking *MYCN* led to impairment of MNA NB cell line migration with concomitant downregulation of genes involved in the migration.

Previous studies have demonstrated mTOR pathway activation in two thirds of neuroblastoma patients, with AKT and mTOR phosphorylation in primary neuroblastoma, and this pathway activation correlated with reduced event-free and overall survival [50–52]. Furthermore, the mTOR pathway is often involved in resistance to cancer therapies [53]. *MYCN* amplification leads to the activation of many downstream pathways, including mTOR, and the mTOR pathway leads to N-Myc protein stabilization [54, 55]. Therefore, the use of mTOR pathway inhibitors in combination with other agents for neuroblastoma therapy has been proposed [56]. However, the mTOR pathway is not a cancer specific pathway and is widely used by non-cancerous cells as well. Thus, mTOR pathway inhibitors are not specific for neuroblastoma cells and present different side effects in normal cells, limiting their potential clinical use. Here we showed that *MYCN* silencing by BGA002 or BGA002-RA led to the inhibition of mTOR pathway gene expression and an overall reduction in pathway activity in MNA-NB cells. *MYCN* expression is manly restricted to cancer cells (and expecially in MNA-NB cells where it is highly expressed), while it has a very limited pattern of expression in normal cells [6]. Thus, our approach of specific *MYCN* targeting by BGA002 may result in the inhibition of the mTOR pathway only in cancer cells, leaving healthy cells unaffected [49].

Furthermore, compared with classical parameters, here we showed that the survival of neuroblastoma patients can be more efficiently predicted by combining mTOR activity, *MYCN*-status, and differentiation.

We also found that autophagy pathways were downregulated in MNA-NB patients, while *MYCN* silencing resulted in autophagy reactivation. BGA002-RA treatment resulted in large increases in lysosomes and macro-vacuoles in MNA-NB cells, particularly in cells that proceeded with apoptosis instead of undergoing differentiation.

## Conclusions

As different studies have highlighted, *MYCN* amplification reshapes the neuroblastoma landscape by creating undifferentiated, aggressive, highly vascularized, disseminating, and nearly untreatable tumors. In this study we showed that specific *MYCN* inhibition by BGA002 led to the reversion of different MNA-NB hallmarks. In combination with RA, BGA002 was able to inhibit migration capacity and induce differentiation or apoptosis, thus overcoming RA-resistance in MNA-NB cells. We also showed that blocking a single cancer-specific gene (*MYCN*) is a more sustainable method for inhibiting the mTOR pathway in neuroblastoma cells only, avoiding side effects of mTOR inhibition in healthy cells. Moreover*,* while N-Myc has been reported to induce angiogenesis, we found that, in a mouse model of highly vascularized MNA-NB, in vivo treatment with BGA002-RA had a dramatic effect on vascularization with absence of tumor blood vessels, which remained after treatment suspension. There was also a significant increase in survival.

Our study shows that it is possible to realize precision medicine, even for the worst type of neuroblastoma (MNA-NB), by the identification of optimal combinated drugs that can achieve potent and selective block of cancer pathways only in tumor cells, preserving the impact of side effects to normal cells. *MYCN* amplification is not restricted to neuroblastoma, and restoration of RA treatment could be beneficial in different MNA-tumors. Therefore, BGA002-RA could potentially be administered to a wide range of aggressive MNA-related malignancies.

## Supplementary Information

**Additional file 1.**

**Additional file 2.**

## Data Availability

The datasets supporting the conclusions of this article are included within the article (and its additional files). Datasets used are described in the material and method section and public available (accession number is provided).

## Abbreviations

MNA: MYCN amplified
NB: Neuroblastoma
RA: Retinoic Acid
PNA: Peptide Nucleic Acid
agPNA: Antigene PNA
BGA002-RA: Combination of BGA002 and RA

## References

[1] Pinto NR, Applebaum MA, Volchenboum SL, Matthay KK, London WB, Ambros PF (2015). Advances in risk classification and treatment strategies for neuroblastoma. J Clin Oncol.

[2] Campbell K, Gastier-Foster JM, Mann M, Naranjo AH, Van Ryn C, Bagatell R (2017). Association of MYCN copy number with clinical features, tumor biology, and outcomes in neuroblastoma: a report from the Children’s oncology group. Cancer.

[3] Weiss WA, Aldape K, Mohapatra G, Feuerstein BG, Bishop JM (1997). Targeted expression of MYCN causes neuroblastoma in transgenic mice. EMBO J.

[4] Brodeur GM, Seeger RC, Schwab M, Varmus HE, Bishop JM (1984). Amplification of N-myc in untreated human neuroblastomas correlates with advanced disease stage. Science.

[5] Seeger RC, Brodeur GM, Sather H, Dalton A, Siegel SE, Wong KY (1985). Association of multiple copies of the N-myc oncogene with rapid progression of neuroblastomas. N Engl J Med.

[6] Huang M, Weiss WA (2013). Neuroblastoma and MYCN. Cold Spring Harb Perspect Med.

[7] Ruiz-Pérez MV, Henley AB, Arsenian-Henriksson M (2017). The MYCN protein in health and disease. Genes (Basel).

[8] Raieli S, Di Renzo D, Lampis S, Amadesi C, Montemurro L, Pession A (2021). MYCN drives a tumor immunosuppressive environment which impacts survival in neuroblastoma. Front Oncol.

[9] Cheung N-KV, Dyer MA (2013). Neuroblastoma: Developmental biology, cancer genomics and immunotherapy. Nat Rev Cancer.

[10] Guglielmi L, Cinnella C, Nardella M, Maresca G, Valentini A, Mercanti D (2014). MYCN gene expression is required for the onset of the differentiation programme in neuroblastoma cells. Cell Death Dis.

[11] Clark O, Daga S, Stoker AW (2013). Tyrosine phosphatase inhibitors combined with retinoic acid can enhance differentiation of neuroblastoma cells and trigger ERK- and AKT-dependent, p53-independent senescence. Cancer Lett.

[12] Zimmerman KA, Yancopoulos GD, Collum RG, Smith RK, Kohl NE, Denis KA (1986). Differential expression of myc family genes during murine development. Nature.

[13] Fletcher JI, Ziegler DS, Trahair TN, Marshall GM, Haber M, Norris MD (2018). Too many targets, not enough patients: rethinking neuroblastoma clinical trials. Nat Rev Cancer.

[14] Tonelli R, Purgato S, Camerin C, Fronza R, Bologna F, Alboresi S (2005). Anti-gene peptide nucleic acid specifically inhibits MYCN expression in human neuroblastoma cells leading to cell growth inhibition and apoptosis. Mol Cancer Ther.

[15] Tonelli R, McIntyre A, Camerin C, Walters ZS, Leo KD, Selfe J (2012). Antitumor activity of sustained N-Myc reduction in rhabdomyosarcomas and transcriptional block by Antigene therapy. Clin Cancer Res.

[16] Montemurro L, Raieli S, Angelucci S, Bartolucci D, Amadesi C, Lampis S (2019). A novel MYCN-specific Antigene oligonucleotide deregulates mitochondria and inhibits tumor growth in MYCN-amplified neuroblastoma. Cancer Res.

[17] Janowski BA, Huffman KE, Schwartz JC, Ram R, Hardy D, Shames DS (2005). Inhibiting gene expression at transcription start sites in chromosomal DNA with antigene RNAs. Nat Chem Biol.

[18] Nielsen PE, Egholm M, Berg RH, Buchardt O (1991). Sequence-selective recognition of DNA by strand displacement with a thymine-substituted polyamide. Science.

[19] Evangelisti C, Paganelli F, Giuntini G, Mattioli E, Cappellini A, Ramazzotti G (2020). Lamin a and Prelamin a counteract migration of osteosarcoma cells. Cells Multidisciplinary Digital Publishing Institute.

[20] Dobrotkova V, Chlapek P, Mazanek P, Sterba J, Veselska R (2018). Traffic lights for retinoids in oncology: molecular markers of retinoid resistance and sensitivity and their use in the management of cancer differentiation therapy. BMC Cancer.

[21] Liu R-Z, Garcia E, Glubrecht DD, Poon HY, Mackey JR, Godbout R (2015). CRABP1 is associated with a poor prognosis in breast cancer: adding to the complexity of breast cancer cell response to retinoic acid. Mol Cancer.

[22] Gupta S, Pramanik D, Mukherjee R, Campbell NR, Elumalai S, de Wilde RF (2012). Molecular determinants of retinoic acid sensitivity in pancreatic Cancer. Clin Cancer Res.

[23] Guidez F, Parks S, Wong H, Jovanovic JV, Mays A, Gilkes AF (2007). RARalpha-PLZF overcomes PLZF-mediated repression of CRABPI, contributing to retinoid resistance in t(11;17) acute promyelocytic leukemia. Proc Natl Acad Sci U S A.

[24] Schug TT, Berry DC, Shaw NS, Travis SN, Noy N (2007). Opposing effects of retinoic acid on cell growth result from alternate activation of two different nuclear receptors. Cell.

[25] Goodman LA, Liu BCS, Thiele CJ, Schmidt ML, Cohn SL, Yamashiro JM (1997). Modulation of N-myc expression alters the invasiveness of neuroblastoma. Clin Exp Metastasis.

[26] Tanaka N, Fukuzawa M (2008). MYCN downregulates integrin alpha1 to promote invasion of human neuroblastoma cells. Int J Oncol.

[27] van Golen CM, Soules ME, Grauman AR, Feldman EL (2003). N-Myc overexpression leads to decreased β 1 integrin expression and increased apoptosis in human neuroblastoma cells. Oncogene Nature Publishing Group.

[28] Ma L, Young J, Prabhala H, Pan E, Mestdagh P, Muth D (2010). miR-9, a MYC/MYCN-activated microRNA, regulates E-cadherin and cancer metastasis. Nat Cell Biol.

[29] Beierle EA, Trujillo A, Nagaram A, Kurenova EV, Finch R, Ma X (2007). N-MYC regulates focal adhesion kinase expression in human neuroblastoma. J Biol Chem Am Soc Biochem Mol Biol.

[30] Noujaim D, van Golen CM, van Golen KL, Grauman A, Feldman EL (2002). N-Myc and Bcl-2 coexpression induces MMP-2 secretion and activation in human neuroblastoma cells. Oncogene Nature Publishing Group.

[31] Cui X, Zhang H, Chen T, Yu W, Shen K (2020). Long noncoding RNA SNHG22 induces cell migration, invasion, and angiogenesis of gastric Cancer cells via microRNA-361-3p/HMGA1/Wnt/β-catenin Axis. Cancer Manag Res.

[32] Sehgal A, Boynton AL, Young RF, Vermeulen SS, Yonemura KS, Kohler EP (1998). Cell adhesion molecule Nr-CAM is over-expressed in human brain tumors. Int J Cancer.

[33] Fakhari M, Pullirsch D, Abraham D, Paya K, Hofbauer R, Holzfeind P (2002). Selective upregulation of vascular endothelial growth factor receptors neuropilin-1 and -2 in human neuroblastoma. Cancer.

[34] Vaughan L, Clarke PA, Barker K, Chanthery Y, Gustafson CW, Tucker E (2016). Inhibition of mTOR-kinase destabilizes MYCN and is a potential therapy for MYCN-dependent tumors. Oncotarget.

[35] Cage TA, Chanthery Y, Chesler L, Grimmer M, Knight Z, Shokat K (2015). Downregulation of MYCN through PI3K inhibition in mouse models of pediatric neural Cancer. Front Oncol.

[36] Schramm A, Köster J, Marschall T, Martin M, Schwermer M, Fielitz K (2013). Next-generation RNA sequencing reveals differential expression of MYCN target genes and suggests the mTOR pathway as a promising therapy target in MYCN-amplified neuroblastoma. Int J Cancer.

[37] Yue M, Jiang J, Gao P, Liu H, Qing G (2017). Oncogenic MYC activates a feedforward regulatory loop promoting essential amino acid metabolism and tumorigenesis. Cell Rep Elsevier.

[38] Tekedereli I, Akar U, Alpay SN, Lopez-Berestein G, Ozpolat B (2019). Autophagy is required to regulate mitochondria renewal, cell attachment, and all-trans-retinoic acid-induced differentiation in NB4 acute Promyelocytic leukemia cells. J Environ Pathol Toxicol Oncol.

[39] Martinez-Outschoorn UE, Peiris-Pagés M, Pestell RG, Sotgia F, Lisanti MP (2017). Cancer metabolism: a therapeutic perspective. Nat Rev Clin Oncol.

[40] Liu GY, Sabatini DM (2020). mTOR at the nexus of nutrition, growth, ageing and disease. Nat Rev Mol Cell Biol.

[41] Villablanca JG, Khan AA, Avramis VI, Seeger RC, Matthay KK, Ramsay NK (1995). Phase I trial of 13-cis-retinoic acid in children with neuroblastoma following bone marrow transplantation. J Clin Oncol.

[42] Masetti R, Biagi C, Zama D, Vendemini F, Martoni A, Morello W (2012). Retinoids in pediatric onco-hematology: the model of acute promyelocytic leukemia and neuroblastoma. Adv Ther.

[43] Giuli MV, Hanieh PN, Giuliani E, Rinaldi F, Marianecci C, Screpanti I (2020). Current trends in ATRA delivery for Cancer therapy. Pharmaceutics.

[44] Freemantle SJ, Spinella MJ, Dmitrovsky E (2003). Retinoids in cancer therapy and chemoprevention: promise meets resistance. Oncogene.

[45] Rickman DS, Schulte JH, Eilers M (2018). The expanding world of N-MYC–driven tumors. Cancer Discov.

[46] Veschi V, Verona F, Thiele CJ (2019). Cancer stem cells and neuroblastoma: characteristics and therapeutic targeting options. Front Endocrinol.

[47] Roche J (2018). The epithelial-to-mesenchymal transition in Cancer. Cancers (Basel).

[48] Rifatbegovic F, Frech C, Abbasi MR, Taschner-Mandl S, Weiss T, Schmidt WM (2018). Neuroblastoma cells undergo transcriptomic alterations upon dissemination into the bone marrow and subsequent tumor progression. Int J Cancer.

[49] Matthay KK, Maris JM, Schleiermacher G, Nakagawara A, Mackall CL, Diller L (2016). Neuroblastoma Nat Rev Dis Primers.

[50] Opel D, Poremba C, Simon T, Debatin K-M, Fulda S (2007). Activation of Akt predicts poor outcome in neuroblastoma. Cancer Res.

[51] Johnsen JI, Segerström L, Orrego A, Elfman L, Henriksson M, Kågedal B (2008). Inhibitors of mammalian target of rapamycin downregulate MYCN protein expression and inhibit neuroblastoma growth in vitro and in vivo. Oncogene.

[52] Iżycka-Świeszewska E, Drożyńska E, Rzepko R, Kobierska-Gulida G, Grajkowska W, Perek D (2010). Analysis of PI3K/AKT/mTOR signalling pathway in high risk neuroblastic tumours. Polish J Pathol.

[53] Burris HA (2013). Overcoming acquired resistance to anticancer therapy: focus on the PI3K/AKT/mTOR pathway. Cancer Chemother Pharmacol.

[54] Chesler L, Schlieve C, Goldenberg DD, Kenney A, Kim G, McMillan A (2006). Inhibition of phosphatidylinositol 3-kinase destabilizes Mycn protein and blocks malignant progression in neuroblastoma. Cancer Res.

[55] Chanthery YH, Gustafson WC, Itsara M, Persson A, Hackett CS, Grimmer M (2012). Paracrine signaling through MYCN enhances tumor-vascular interactions in neuroblastoma. Sci Transl Med.

[56] King D, Yeomanson D, Bryant HE (2015). PI3King the lock: targeting the PI3K/Akt/mTOR pathway as a novel therapeutic strategy in neuroblastoma. J Pediatr Hematol Oncol.

