# Supplementary material for: The MYCN inhibitor BGA002 restores the retinoic acid response leading to differentiation or apoptosis by the mTOR block in MYCN-amplified neuroblastoma
Source: J Exp Clin Cancer Res. 2022 Apr 30;41:160. doi: 10.1186/s13046-022-02367-5 (PMC9055702; doi:10.1186/s13046-022-02367-5)
Supplement: Supplementary file 1 — Additional file 1. [file 13046_2022_2367_MOESM1_ESM.docx]

**Supplementary material and methods**

**Synergy calculation**

The combination index plot was calculated for each cell line using all the biological replicates (the procedure was the same for MYCN mRNA inhibition or for cell growth inhibition). Data were inserted in Compusyn according to the guidelines (1,2). The combination index (CI) and fractional inhibition (FA) are obtained from the compiled report. As described in the original article, the combination index equation defines the synergism (CI < 1), additive effect (CI = 1) and antagonism (CI > 1)(3).

**Confocal microscope analysis for lysosome**

Kelly and LAN-5 neuroblastoma cell lines are seeded 30.000 cell/cm^2^ 24 hours before treatment in Lab-Tek Chambered Coverglass (Thermofisher: 155411 16/PK) using OPTIMEM at 4% FBS. Culture medium is changed with fresh OPTIMEM and cells are treated with BGA002, 13-cis RA, or both at concentration of 2.5 μM. After 6 hours, FBS is added up to 4%. Cells are treated for 48 hours.

For staining, culture medium is removed and cells are incubated with Lysosome staining kit (Green Fluorescence) (Abnova: KA4111) and 25 nM of MitoTracker™ Deep Red FM (Thermofisher:M22426), at 37°C for 45 minutes. After the incubation, staining solution is removed and cells are washed twice with filtered NaCl 0.9% for about 2 minutes. Fresh culture medium is added at last.

Images are acquired using confocal microscope Nikon Eclipse Ti2-E, in oil immersion with 100x objective. Z-stacks are acquired using 0.2 μm Z-step. Image are analyzed using NIS-Element viewer.

Image analysis is performed with Fiji. For mitochondrial footprint evaluation, Z-stack ROIs are defined for each single cell, and images are processed using MiNA approach (4). Mitochondrial areas are measured using Fiji measure tool. Mitochondrial volumes are measured as sum of mitochondrial area multiplied for Z-step (0.2 μm).

**Confocal microscope analysis for cell line differentiation**

LAN-5 cell line is seeded in 6-well plate prepared with 24x24 mm sterilized cover glass. Differentiation protocol is applied as described before. After 9 days, cells are washed with pre-warmed PBS 1x, then fixed using 10% formaldehyde solution for 15 minutes at room temperature. After fixing, cells are incubated at -20°C with methanol for 20 minutes to allow permeabilization. Cells are washed again for three times with PBS 1x, incubated at +4°C for 1 hour with blocking solution (BSA 2% in PBS 1x) then blocking solution is removed and Synapsin-1 Alexa Fluor 488 Conjugated antibodies (#13197 Cell Signaling) is resuspended in blocking solution and added at +4°C overnight. After antibody incubation, each sample is briefly washed and counterstained with DAPI solution 1x for 1 minute. Samples are mounted using anti-fade before observation.

For each sample, 20 z-stacks (0.35 μm each) are acquired using 60x magnification. For each z-stack, mean fluorescence intensity per cell is measured using the CTCF (Corrected Total Cell fluorescence) method. In particular, it is not possible to measure CTCF for single cells due to heavy cluster organization, so we measure fluorescence intensity of the whole section using the CTCF method then we divided the value for the number of cells. To compare different treatment, we use 2-sided unpaired t-test (5,6).

**Patient gene expression profiles**

The neuroblastoma dataset (accession: E-MTAB-1781) (7) was downloaded from ArrayExpress, (http://www.ebi.ac.uk/arrayexpress) and processed using the quantile algorithm in *limma* (8). Another neuroblastoma dataset was download from TARGET data portal (https://ocg.cancer.gov/programs/target; data freely accessible) (9). The replicate probes within the array were replaced by their average before being scaled. Pearson correlation between MYCN and other genes was calculated with R software. The differential expressed genes between MYCN-amplified (MNA) patients and not-MNA patients in the neuroblastoma datasets (E-MTAB-1781, TARGET dataset) were obtained using the *limma* package algorithm (clinical information were retrieved from the dataset meta-data, patient with an unknown MYCN amplification status were removed). Clinical information of the selected gene expression profiles is reported in Supplementary Table 3 and Supplementary Table 4.

**Pathway analysis**

Correlated genes with MYCN in each dataset were used as ranked gene list to identify enriched pathway through Gene Set Enrichment Analysis (GSEA)(10,11). Same procedure was used for differential expressed genes between MYCN amplified versus MYCN not amplified gene expression profiles. We used Gene Ontology (biological process, cellular component, molecular function, C5 from Molecular Signatures Database v7.0) and GSEA software (V. 4.02). Graphic representation was performed with R software.

We used *rrvgo* (12,13) package to reduce redundancy of the Gene Ontology tree. Briefly, we used the pathway anti-correlating with MYCN in NB1 and NB2 cohorts obtained from GSEA analysis and we mapped the pathway names to the respective GO ID using *GO.db* R package (14). We calculate the semantic similarity matrix using *calculateSimMatrix* (first 100 pathways, using Biological Process (BP) as parameter and standard parameters). We used *reduceSimMatrix* (using the FDR as score, threshold = 0.7 and maintaining the other standard parameters) to reduce the GO terms. We then plotted the treemap (15,16) for the pathways.

**Cluster selection**

Genes involved in neuronal differentiation were selected by Gene Ontology (GO terms concerning neuron differentiation or neuron component, all GO terms version C5 from Molecular Signatures Database v7.0) or known from literature (the list of which is available in Supplementary Table 5). Neuroblastoma gene expression profile (E-MTAB-1781) were filtered for the differentiation gene list. We use Uniform Manifold Approximation and Projection (UMAP) (17,18) as dimension reduction algorithm. UMAP was computed with a minimum distance of 0.2, considering 100 local neighbours and selecting the Euclidean distance as metric. *HDBSCAN* (19,20) was conducted in order to identify clusters on the UMAP projection (minimum samples size of 5, minimum cluster size of 100). The following libraries from Python (version 3.7) was used for the analysis: *UMAP-learn*, *scikit-learn*, *matplotlib.pyplot*, *Pandas*, *seaborn*, *hdbscan*, *numpy*. The following libraries from R (version 3.5) were used: *ggplot2*, *dplyr*, *data.table*, *tydr.*

**Differentiation Convolutional Neural Network**

Optical microscope images of SH-SY5Y cell line differentiated in presence or not of retinoic acid for 9 days were used to build an image dataset. Images were randomly cropped to the 512 x 512 pixel size. Images without any cells inside were removed from the image dataset. From the dataset, images were randomly picked (for each experimental condition: 3000 for training set, 500 for validation set and 500 for test set, no image was shared between the tree sets). We used ResNet50 (21,22) pre-trained model (trained on Imagenet) for transfer learning (22). Images were pre-processed with the pre-process input. Sample augmentation was conducted for the training set (random zoom (0.3), random rotation (50 degree), width and height shift (range 0.2), horizontal flip and we used as fill mode “nearest”). ResNet50 model was incorporated in a new model (scheme of the model is presented in Supplementary Table 6 and supplementary figure 11) and were trained on the training set using binary cross-entropy as loss function and *RMSprop* (23–27) as optimizer (learning rate of 0.0001, decay of 1x10^-6^) for 5 epochs. Model was fine-tuned unfreezing the last ResNet50 block using binary cross-entropy as loss function and *SGD* (28,29) as optimizer (learning rate of 0.00001, momentum of 0.9, decay of 1x10^-6^) for 3 epochs. AUC, ROC, confusion matrix and other statistics (Supplementary Table 7) were calculated on the test set. The trained Model indicated as Convolutional Neural Network (CNN) differentiation was saved and used for the analysis below. Optical microscope images of different cell line (LAN-5, Kelly, SK-N-BE(2)-c) differentiated in presence of retinoic acid, BGA002, retinoic acid-BGA002 for 9 days were used in the analysis. Images were randomly cropped to the 512 x 512 pixel size. Images without any cells inside were removed from the image dataset. We used the CNN differentiation as feature extractor (30–33) (we extracted the feature vectors using the outputs of the fully connected dense layer Dense 9). UMAP was computed with a minimum distance of 0.2, considering 10 local neighbours and selecting the Euclidean distance as metric. For each feature vectors of the Control condition, we calculated the Euclidean distance between the Control (cell in medium alone) feature vector and each feature vector of the other experimental condition (retinoic acid, BGA002, retinoic acid-BGA002). Considering as x a Control feature vector and y as feature vector of another experimental condition, we consider the mean of the Euclidean distance (d) of each x for each y:

$$\frac{\sum dx_{i}y_{i}}{n_{y}}$$

Summary scheme of the analysis is presented in Supplementary figure. The following libraries from Python (version 3.7) was used for the analysis: *os*, *glob*, *scipy*, *keras*, *UMAP-learn*, *scikit-learn*, *Eli5*, *matplotlib.pyplot*, *Pandas*, *seaborn*, *hdbscan*, *numpy*. The following libraries from R (version 3.5) were used: *ggplot2*, *dplyr*, *data.table*, *tydr*.

**Gene Regulatory Network**

Microarray data containing neuroblastoma cell lines treated for different days with retinoic acid were downloaded from NCBI GEO dataset (accession number: GSE9169) (34). We included in this study only the SH-SY5Y treated with retinoic acid. We log-transformed the data and we substitute the mean of the probes for each gene. We then selected only the most variant genes (variance higher than 1). Transcription factors gene list were downloaded from AnimalTFDB (only human transcription factors were considered). To infer GRN, correlation between transcription factors (tfs) and the other genes we used the GENIE3 algorithm (35). We performed 25 iterations of the algorithm to reduce background. We average the obtained regulatory weight and select only the third upper quartile to avoid false positive. We used these regulatory interactions to build the network with *iGraph*. We deleted not connected vertices, and we extracted the biggest component with CINNA (36). We used CINNA’s *proper_centralities* function to identify the most relevant centrality measures. Once calculated the centrality measures, we selected the transcription factors and we used t-Distributed Stochastic Neighbour Embedding analysis (t-SNE) (37) to distinguish which measure has more information (t-SNE cost) and then more relevant. We then selected the transcription factors and ranked them according to eigen vector centrality (we selected only the tfs with an eigen vector centrality value higher than the median). List of the identified transcription factor is present in Supplementary table 8. We filtered the most variant genes in the two neuroblastoma cohorts used in this study (E-MTAB-1781 and TARGET). We used RTN package (38–40) to reconstruct the GRN network in both the cohorts, we used the list of the tfs identified above. This computed a list of regulons and their putative targets. We used the obtained regulons for the enrichment analysis in both cohorts, we selected the common regulons between the two cohorts (supplementary tables 9 and 10). We then calculated the activity of this regulons in the two cohorts and we used on the regulon activity the hierarchical cluster (Euclidean distance). Using these three clusters, we divided the patient gene expression profiles in three group (patient regulated group). With hierarchical clustering on their activity, we divided the regulons in three different clusters. In the supplementary table 11, it is presented the list of gene regulated by the corresponding regulons and their corresponding regulon cluster. We then conduced pathway enrichment on the regulon regulated genes using *anRichment* package and the results are presented in the supplementary table 12. In this way, we identify three cluster of patients based on the regulon activity. We drew the survival curve using *survminer* and *survival* packages for the three-patient regulation group (supplementary table 13 and 14) (41).

The following libraries from R (version 3.5) were used: *dplyr* (v. 0.8.3), *tydr* (v. 1.0.0), *limma* (v. 3.38.3) *GENIE3* (v. 1.4.3), *CINNA* (v. 1.1.53), *igraph* (v. 1.2.4.2), *pheatmap* (0.12), *RTN* (2.12.0).

**CCLE gene expression**

mTOR pathway genes were obtained from literature(42–50) (list of the genes in the pathway are present in the supplementary table 15). We downloaded the RNAseq expression from Cancer Cell Line Encyclopedia (51,52) (<https://portals.broadinstitute.org/ccle>), we excluded the cell line where the tumor origin were not reported and we generated boxplot from the downloaded data. From the Cancer Cell Line Encyclopedia, we downloaded also the methylation data for the same mTOR pathway genes. Data was normalized for each mTOR pathway gene (z-score) and we calculated the average for each tumor type. We use the normalized mTOR pathway gene for t-SNE (t-distributed Stochastic Neighbour Embedding) analysis using R library *Rtsne* (v. 0.15) setting perplexity of 300, high accuracy, learning rate of 20 and 1000 maximum iterations.

A list of N-Myc ChIP dataset results was downloaded from AnimalTFDB (53). The MYCN ChIP Score was calculated as the average of the score of each ChIP set, we considered only the genes whose score was higher than the median. Protein Protein Interactions (PPI) were downloaded from the STRING (54) database (binding annotation and other metadata were downloaded from the same source) and all the interaction with a combined score lower than 400 were filtered out, we downloaded the latest version (version 11, downloaded May 2020). Data from MYCN ChIP Score and PPI interaction in mTOR pathway were combined and then data were graphed with *iGraph* R.

**ChIP-seq analysis**

ChIP-Seq data for SK-N-BE(2)c for chromatin IP against H3K27ac, N-Myc and H3K4me3 were downloaded from GEO dataset (GSE80151) (55). ChIP-Seq data for SHEP21 Tet-off (or also called Tet21N) for chromatin IP against H3K27ac, N-Myc and POLII were downloaded from GEO dataset (GSE80151). The ChIP-Seq data were aligned to the human genome (GRCh37/HG19), the signal peaks were visualized in Integrative Genomics Viewer (IGV, version: 2.10.0)(56,57).

**mTOR pathway susceptibility**

Cell line drug response was downloaded from Sanger Institute (58). MYCN amplification status for each cell line was retrieved from literature or from expression data on Sanger Institute. Data was filtered for the pathways on which were acting (IGF1R signaling, PI3K/MTOR signaling, p53 pathway) and for neuroblastoma. NB1 cohort (E-MTAB-1781) was stratified according to the mTOR genes expression. The expression of the genes present in the mTOR genes signature was averaged and the normalized (z-score calculation), gene expression profile (GEP) with z-score higher than 1 were considered high expression, z-score comprised between 0 and 1 as medium expression, while less than 0 as low expression.

**Combined score and survival ROC**

NB1 cohort (E-MTAB-1781) was used to generate the score. To build the mTOR score, the expression of the genes present in the mTOR genes signature was averaged and the normalized (z-score calculation). Other clinical characteristics used for the ROC curves (MYCN status, age, sex and stage) were retrieved in the clinical associated metadata of the dataset.

To build the differentiation score, we followed a similar procedure as in (59). Briefly, we started from the list of genes obtained by the Gene Regulatory Network. We selected the genes that were regulon target both in the NB1 and NB2 (TARGET) cohort. We filtered NB1 cohort for the list of the genes that are present in the common gene list (543 genes). Therefore, we used univariate Cox regression analysis to select only the genes there was significantly associate to the prognosis (we corrected the p-value with the Bonferroni correction). These gene lists are present in supplementary tables 16 and 17. On the obtained gene list, we build a multivariate Cox regression model, the model was regularized with Lasso penalization (we used the *Penalized* R package, λ1 (lambda1) parameter set to 1) (60). After converging, we obtained a list of 230 genes and respective coefficients. We selected only the genes with coefficients higher than 1 or lower than -1. The selected genes are present in supplementary table 18.

This resulted in selecting two different vectors associated with the prognosis, one with comprising positive weights (associated with an increase in the hazard) and one with negative weights (associated with a reduction in the hazard).

We calculated a positive and negative score, multiplying each gene x_i_ for his associated weight:

$${score}^{P}=\frac{1}{n}\sum_{i=1}^{n} x_{i}\cdot\left| w_{i}^{P} \right|$$

$${score}^{N}=\frac{1}{n}\sum_{i=1}^{n} x_{i}\cdot\left| w_{i}^{N} \right|$$

As defined above with:

$$w_{i}^{P}>1$$

$$w_{i}^{N}<-1$$

The differentiation score was defined as the ratio between the two score and

$$score= \frac{{score}^{P}}{{score}^{N}}$$

We then applied standardization, the normalized score is obtained subtracting the mean and dividing for the standard deviation:

$$\hat{s}=\frac{s-\bar{s}}{sd(s)}$$

The combined score was built with Cox regression model using the mTOR, differentiation score and MYCN status for NB1 cohort (supplementary table 19).

The time-dependent receiver operator curves (ROC) for overall survival was built as described (61,62). Briefly, we used NB1 cohort and cox regression model described above. We choose as t threshold: 1, 3 and 5 years (360, 1080, 1800 days) and 1 is considered death, while 0 alive at the time of censoring. We used the following package in R (*survival*, *survminer*, *survivalROC*).

**Autophagy signature**

Tet21N gene expression profiles were downloaded from NCBI (GSE80153). Data was log2 transformed, not-expressing and low variant genes were filtered. We selected the genes from the pathway in the GO database (specifically the following pathways: GOPOSITIVEREGULATIONOFAUTOPHAGY, GOAUTOPHAGOSOMEORGANIZATION, GOAUTOPHAGOSOME, GOPREAUTOPHAGOSOMALSTRUCTURE, GOAUTOPHAGOSOMEMEMBRANE, GOREGULATIONOFAUTOPHAGY). Data was normalized for each pathway (z-score) and we calculated the average for each pathway type and for each time point.

**Histology image processing and immuno-histochemistry quantification**

For each condition around 200 images were acquired. The following pre-processing steps were conducted on the whole image and in batch. Image were processed using Fiji (63–65) to enhance separation between diaminobenzidine (DAB) and hematoxylin (HE). Briefly, we removed the background, color correct the image to improve red and blue separation and to increase overall image contrast (the same modifications are applied to each image under analysis).

Processed images were analyzed on python, each image was randomly divided in 20 images of 512 x 512 pixels. The separation of the immunohistochemical (IHC) diaminobenzidine (DAB) staining from the hematoxylin (HE) counterstaining was conducted with the method described in literature (66). The analysis was conducted on the separate channel images. Briefly, we used Otsu threshold, removed small object, eroded and filled the gap to select the stained cells and then we segmented the cell using watershed segmentation. The analysis were done using the *scikit-image* library in python (67). After segmentation, the number of individual cells were counted. We calculated the ratio for each image between the number of DAB-stained cells (DAB_n_) and the number of HE-stained cells (HE_n_) for each image:

$$ratio=\frac{{DAB}_{n}}{{HE}_{n}}$$

The following libraries from Python (version 3.7) was used for the analysis: *Scikit-image*, *Pandas*, *Numpy*, *imageio*, *scipy*.

**In vivo supplementary analysis**

Animal weight are recorded before the study and at the moment of euthanasia, corresponding to the end point set for the EFS analysis. Animal general condition are further monitored during the entire study and recorded using specific HET (Humane Endpoint Table). According to veterinary, animal welfare is maintained during all experimental procedure and none of the animal reach a score for safety assessment, equal or higher than threshold value established, in according to animal protocol.

All treatments are administered to each animal daily for 28 days. BGA002 is administered through subcutaneous injection (s.c.) at 10 mg/kg/day, 13-cis RA is administered through intraperitoneal injection (i.p.) at 10 mg/kg/day and 0.9% NaCl is administered through subcutaneous injection. BGA002 and 13-cis RA are administered in combination both at 10 mg/kg/day using their own way of administration.

**Supplementary Figure legends**

**
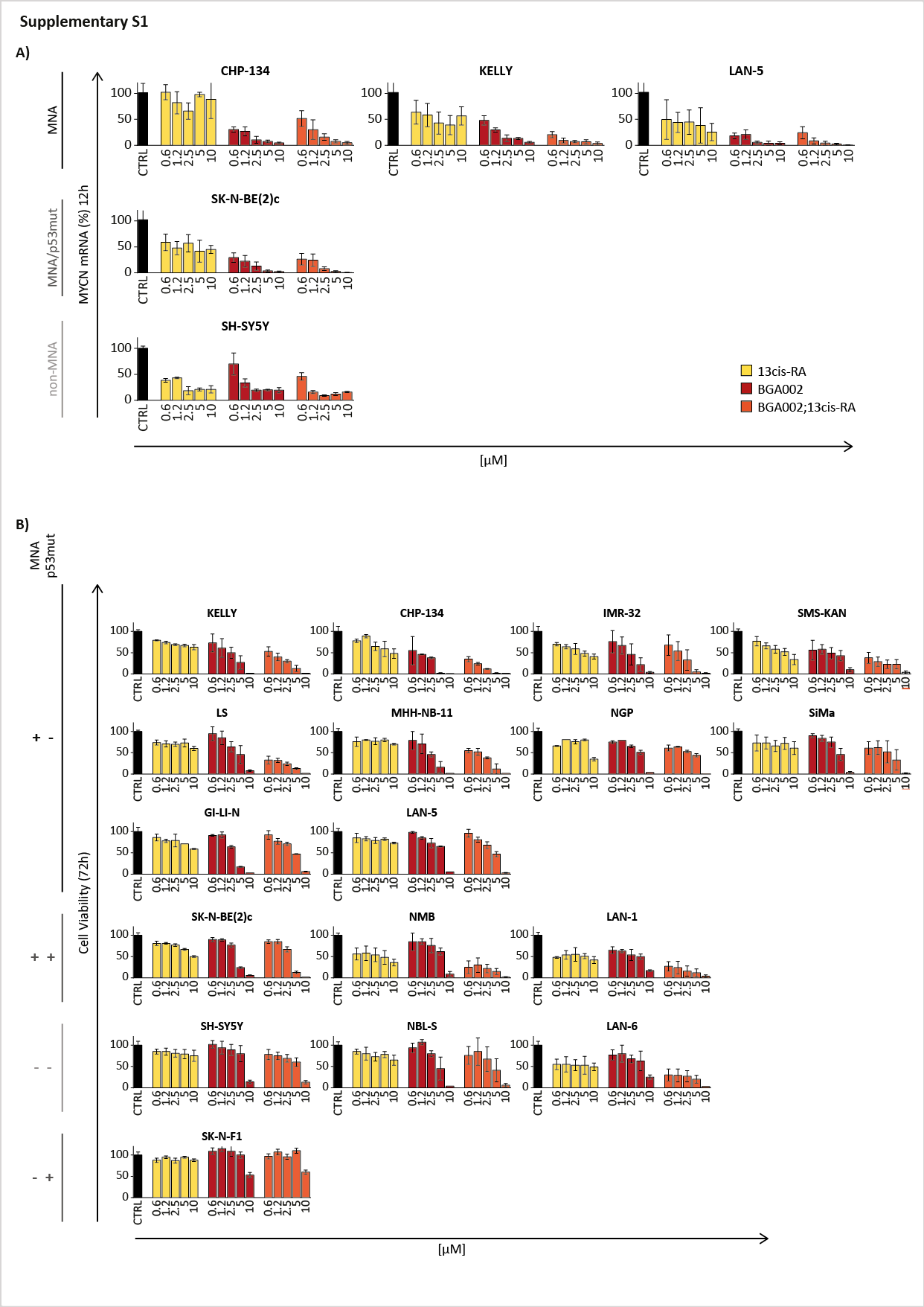
**

**Supplementary figure 1.**

**BGA002 in combination with RA downregulate MYCN NB expression and affects cell viability**

**A,** Neuroblastoma cell lines treated with increasing doses (13-cis Retinoic Acid, BGA002, BGA002 + 13-cis Retinoic Acid) for 12 hours. The MYCN mRNA expression was evaluated through RT-PCR (n = 3 biological replicates). Columns represent the mean percentage of MYCN mRNA normalized over the control, the whiskers represent the standard deviation. Neuroblastoma cell lines are ordered according to their MYCN amplification and p53 mutation status (first row: MNA p53^wt^ NB cell lines, second row: MNA p53^mut^ NB cell lines, third row: non-MNA p53^wt^ NB cell lines). **B,** Neuroblastoma cell lines treated with increasing doses (13-cis Retinoic Acid, BGA002, BGA002 + 13-cis Retinoic Acid) for 72 hours. The cell viability was evaluated through luminescence assay (n = 3 biological replicates). Columns represent the mean percentage of cell viability normalized over the control, the whiskers represent the standard deviation. Neuroblastoma cell lines are ordered according to their MYCN amplification and p53 mutation status (first three rows: MNA p53^wt^ NB cell lines, fourth row: MNA p53^mut^ NB cell lines, fifth row: not-MNA p53^wt^ NB cell lines, sixth row: MNA p53^mut^ NB cell lines).

**
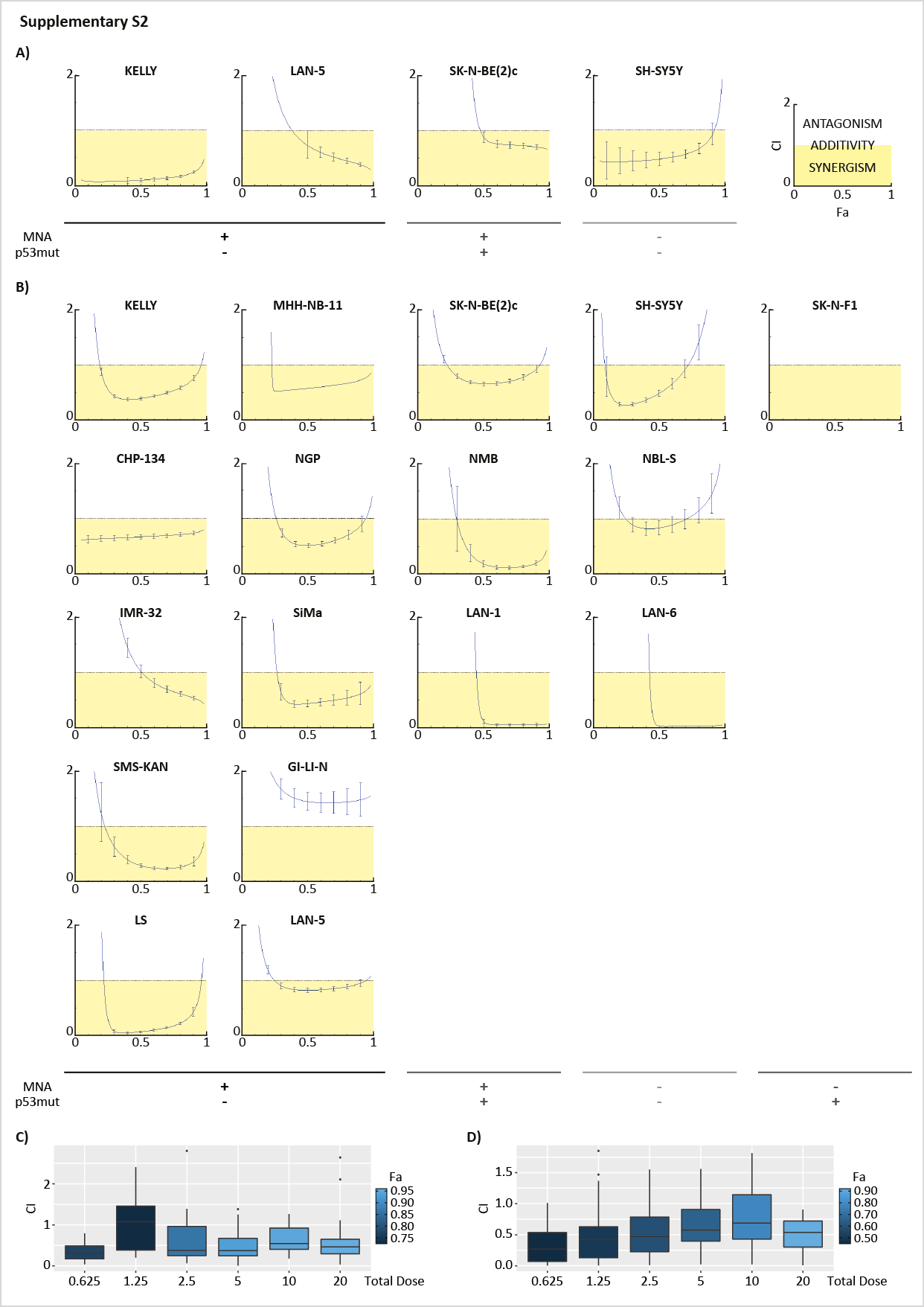
**

**Supplementary figure 2.**

**BGA002 in combination with RA shows synergic effect**

**A,** Neuroblastoma cell lines treated with increasing doses (13-cis Retinoic Acid, BGA002, BGA002 + 13-cis Retinoic Acid) for 12 hours. The MYCN mRNA expression was evaluated through RT-PCR (n = 3 biological replicates). Fa-CI plot (combination index (CI) and fractional inhibition (FA)) for MYCN mRNA in NB cell lines. **B,** Neuroblastoma cell lines treated with increasing doses (13-cis Retinoic Acid, BGA002, BGA002 + 13-cis Retinoic Acid) for 72 hours. The cell viability was evaluated through luminescence assay (n = 3 biological replicates). Fa-CI plot (combination index (CI) and fractional inhibition (FA)) for cell viability decrease. Neuroblastoma cell lines are ordered according to their MYCN amplification and p53 mutation status (first two columns: MNA p53^wt^ NB cell lines, third column: MNA p53^mut^ NB cell lines, fourth column: not-MNA p53^wt^ NB cell lines, fifth column: MNA p53^mut^ NB cell lines).**C-D,** Box plot summarizing the synergistic effect for each dose. The vertical axis represents the CI value, while the color scale represents the FA value. Each dot represents the CI value for a single cell line experiment, the middle line is representing the median while the box limits indicate the first and the third quartiles and whiskers specify samples comprised 1.5 times the interquartile range.**C,** Box plot representing MYCN summarizing mRNA MYCN inhibition. **D,** Box plot representing MYCN summarizing cell viability inhibition.

**
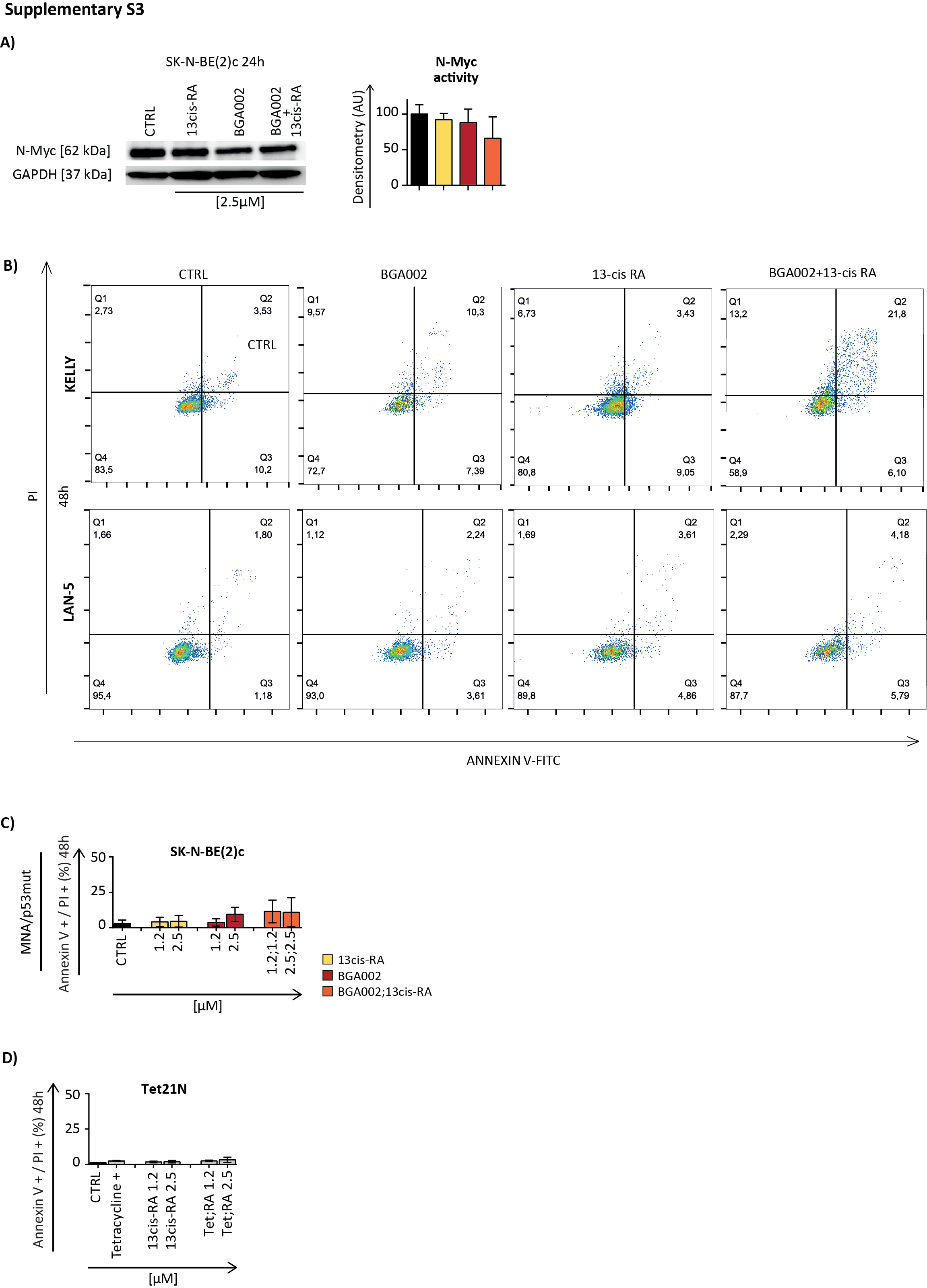
**

**Supplementary figure 3.**

**BGA002 in combination with RA blocks N-Myc protein expression**

**A,** Representative western blot analysis after 48 hours of treatment (n = 3 experiment for cell line). Left, representative staining for N-Myc (top) and associated GAPDH staining (down). N-Myc quantification normalized over the GAPDH is presented on the right, the bars represent the mean of 3 experiments, the whiskers represent the standard deviation. western blot analysis for SK-N-BE(2)-c cell line. **B-D,** Apoptosis measurement after 48 hours of treatment (n = 3 experiment for each cell line). Barplots represent the percentage of cells stained by AnnexinV^+^/PI^+^, bars represent mean, whiskers the standard deviation. **B,** Cross-graph for Kelly and LAN-5 at 48 hours, stained with cells stained by AnnexinV^+^/PI^+^ .**C,** Apoptosis measurement for SK-N-BE(2)-c cell line. **D,** Apoptosis measurement for Tet21N cell line (cell line with inducible MYCN promoter where tetracycline is inhibiting the MYCN transcription).

**
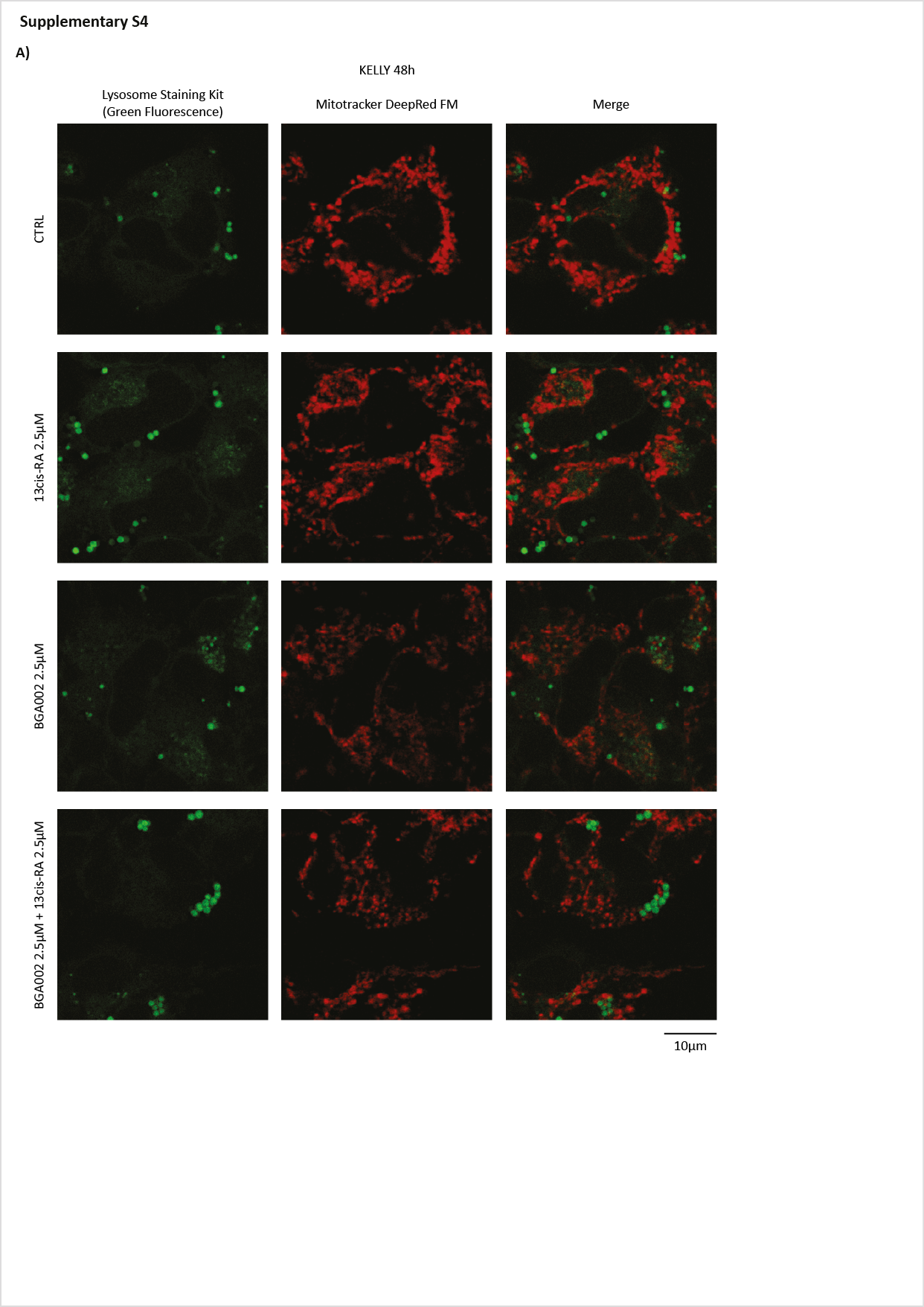
**

**Supplementary figure 4.**

**BGA002 in combination with RA reduces dramatically mitochondria in Kelly cell line**

Neuroblastoma MNA cell line Kelly treated for 48 hours (CTRL: medium alone, RA: retinoic acid 2.5 µM, BGA002: BGA002 2.5 µM, BGA002 + RA: BGA002 2.5 µM and retinoic acid 2.5 µM). Cell line was fixed and stained with lysosome staining kit (left column, green) and Mitotracker (middle column, red). The merge is presented in the right column. Representative experiment (n = 2 biological replicates).


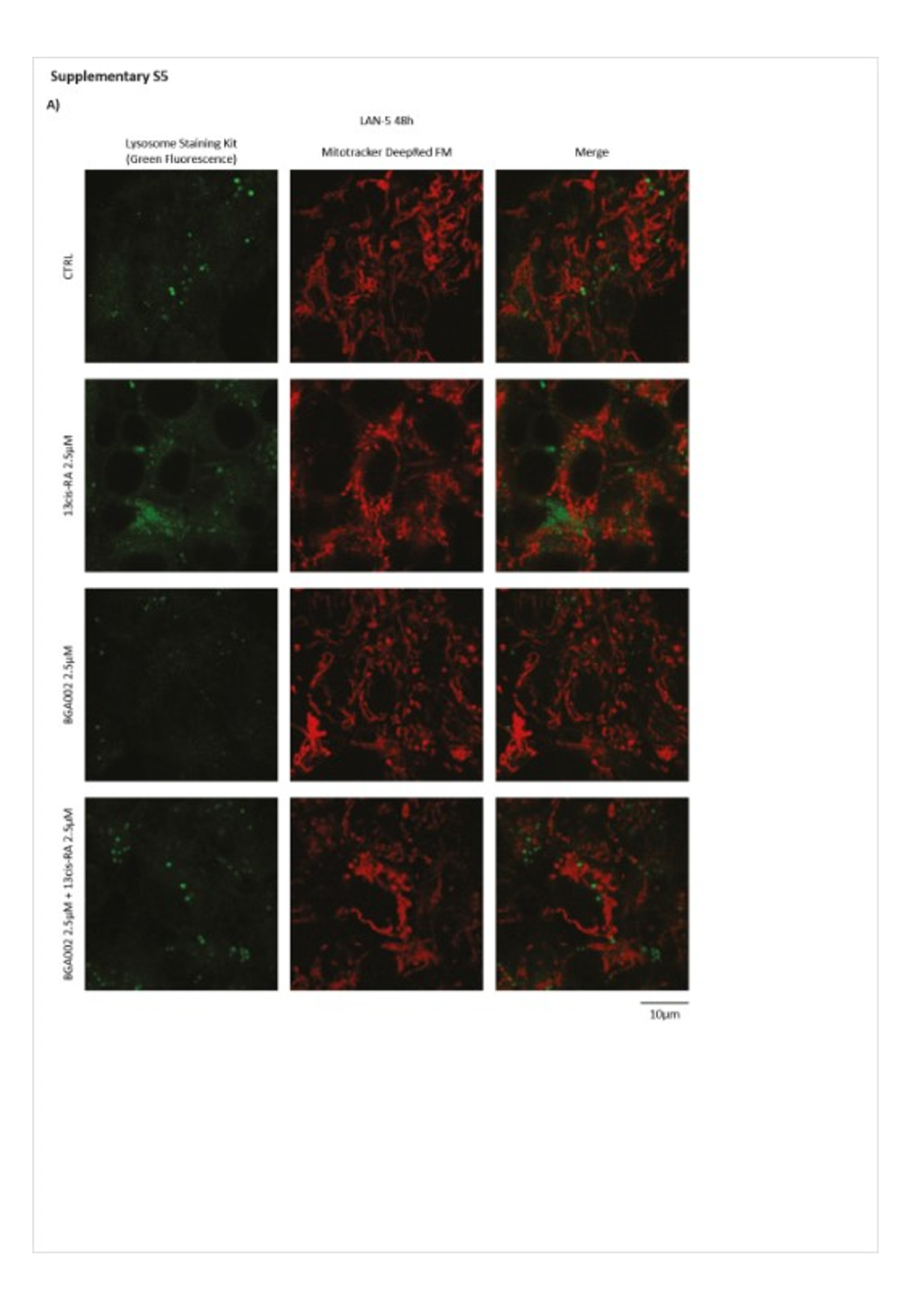


**Supplementary figure 5.**

**LAN-5 mitochondria are less affected by the treatment with BGA002 in combination with RA**

Neuroblastoma cell lines MNA LAN-5 treated for 48 hours (CTRL: medium alone, RA: retinoic acid 2.5 µM, BGA002: BGA002 2.5 µM, BGA002 + RA: BGA002 2.5 µM and retinoic acid 2.5 µM). Cell line were fixed and stained with lysosome staining kit (left column, green) and Mitotracker (middle column, red). The merge is presented in the right column. Representative experiment (n = 2 biological replicates).

**
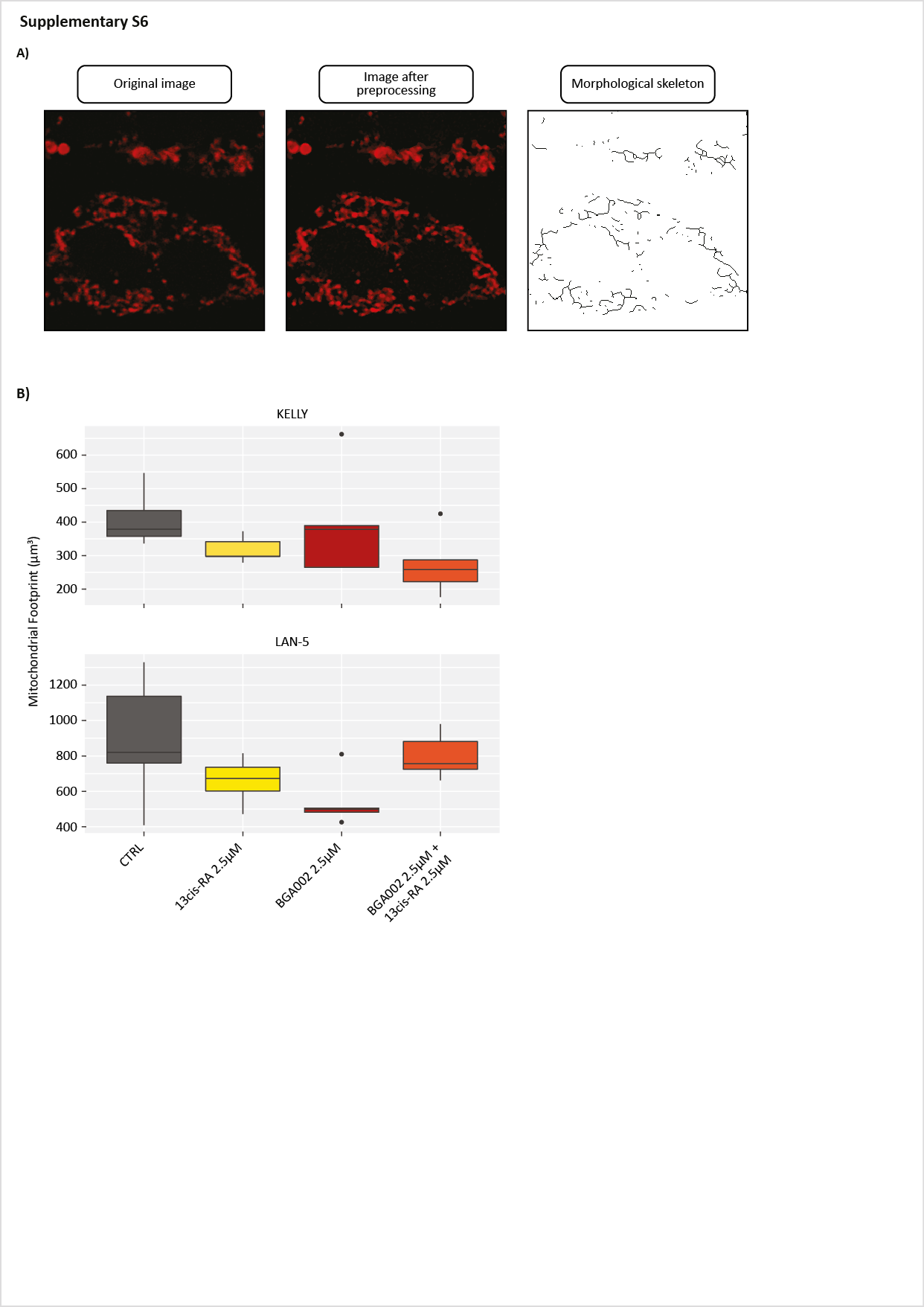
**

**Supplementary figure 6.**

**LAN-5 mitochondria volume is less affected by the treatment with BGA002 in combination with RA**

**A,** Schematic representation of the analysis conducted on the confocal images. Detailed description of the method is presented in the Supplementary Methods section above. **B,** Mitochondria volume quantification for MYCN amplified cell lines after 48 hours treatment (CTRL: medium alone, RA: retinoic acid 2.5 µM, BGA002: BGA002 2.5 µM, BGA002 + RA: BGA002 2.5 µM and retinoic acid 2.5 µM). Top panel represents relative quantification for LAN-5, bottom panel refers to Kelly cell line. Each dot represents the volume measurement for a single mitochondrion, the middle line is representing the median while the box limits indicate the first and the third quartiles and whiskers specify samples comprised 1.5 times the interquartile range. The graph represents the results of the pooled quantification of 5 cells (randomly selected).

**
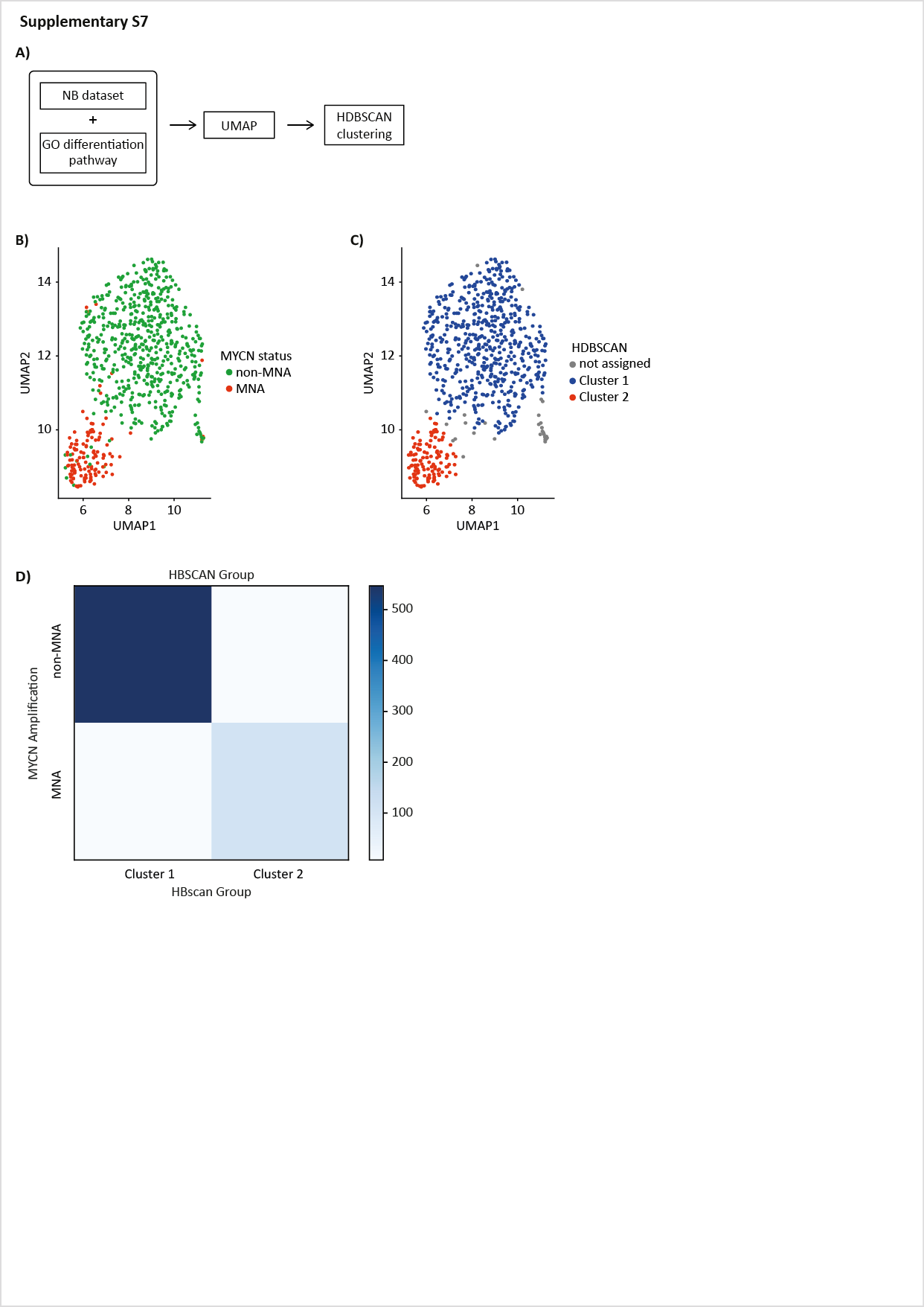
**

**Supplementary figure 7.**

**MNA patients are differently clustering from not-MNA patients for differentiation**

**A,** Schematic representation of the analysis conducted on the NB1 cohort (E-MTAB-1781) gene expression profiles (GEP). UMAP was conducted on the GEP filtered for genes involved from differentiation (selected from GO database). UMAP projection of the filtered NB1 dataset was then used for clustering (HDBSCAN). **B,** UMPA projection of the NB1 cohort and the MYCN status representation. Each dot represents a GEP. First two embedding components are plotted (UMAP1, UMAP2). **C,** UMAP projection of the NB1 cohort with the HDBSCAN results. Each dot represents a GEP, grey dots represent GEP not assigned to any cluster (cluster 1: 555, cluster 2: 117, not assigned: 30). First two embedding components are plotted (UMAP1, UMAP2). **D,** Heatmap showing the distribution of MNA patient between HDBSCAN groups (cluster 1 and MNA: 8, cluster 1 and not-MNA: 547,cluster 2 and MNA: 107, cluster 2 and not-MNA: 10). Color scale indicates the number of observations.

**
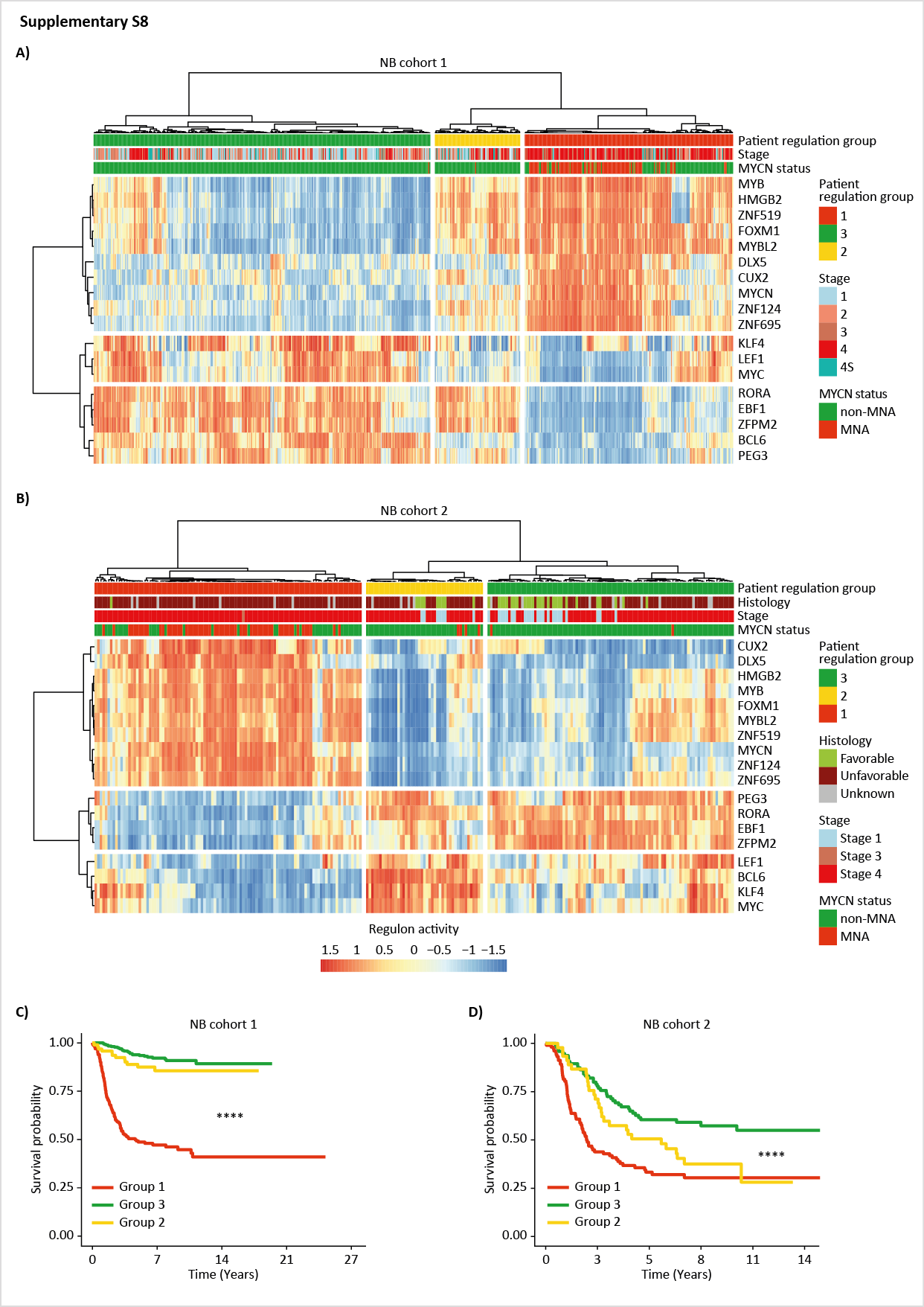
**

**Supplementary figure 8.**

**Differentiation transcriptional network identifies patients is linked to prognosis outcome in NB patients**

**A,** Heatmap indicating the normalized relative abundance of regulons in NB1 cohort (E-MTAB-1781). Hierarchical cluster on the columns and on the rows were conducted on the regulation activity using Euclidean distance as metric. Clinical information (retrieved from the dataset metadata) is listed on top (stage, MYCN status). **B,** Heatmap indicating the normalized relative abundance of regulons in NB2 cohort (TARGET). Hierarchical cluster on the columns and on the rows were conducted on the regulation activity using Euclidean distance as metric. Clinical information (retrieved from the dataset metadata) is listed on top (Histology, stage, MYCN status). **C,** Kaplan-Meyer plot for the probability of overall survival over time for patients associated with regulon patient group for NB1 cohort (group 1, n = 232; group 2, n = 375; group 3, n = 95). Associated p value is shown in the middle of the plot (log-rank test). **D,** Kaplan-Meyer plot for the probability of overall survival over time for patients associated with regulon patient group for NB2 cohort (group 1, n = 103; group 2, n = 95; group 3, n = 42). Associated p value is shown in the middle of the plot (log-rank test).

**
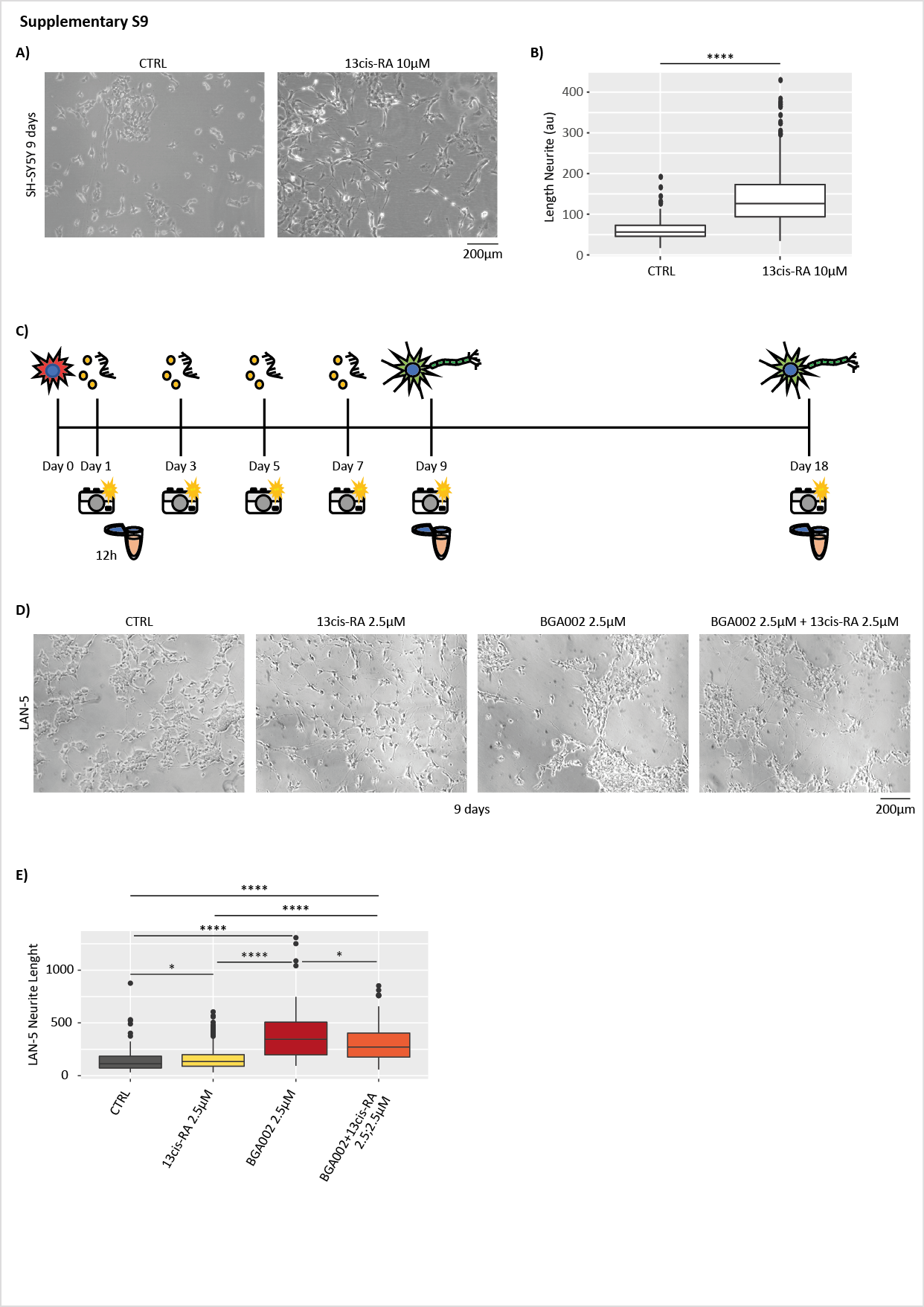
**

**Supplementary figure 9.**

**BGA002 in combination with RA induces differentiation in MYCN amplified cell lines**

**A,** Optical microscope image of SH-SY5Y treated for 9 days (with CTRL (medium alone) or with retinoic acid). Cell images were acquired at the 9^th^ day. Representative image of 1 of 2 biological replicates. **B,** Box-plots represent the length of neurite in SH-SY5Y after 9 days of treatment (with CTRL (medium alone) or with retinoic acid). Each dot represents the measurement for a single neurite, the middle line is representing the median while the box limits indicate the first and the third quartiles and whiskers specify samples comprised 1.5 times the interquartile range. The graph represents the results of two pooled experiments. Statistic: Wilcoxon matched pair test. **C,** Schematic representation of the *in vitro* MNA-NB cell lines treatment. Kelly, LAN-5, SK-N-BE(2)-c are seeded the day before to start the treatment and then the day after treated with different treatments (CTRL: medium alone, RA: retinoic acid 1.25 µM, BGA002: BGA002 1.25 µM, BGA002 + RA: BGA002 1.25 µM and retinoic acid 1.25 µM). The treatment is repeated every 2 days and each time optical images are acquired and dendrite length is measured (days: 1, 3, 5, 7, 9). At the 9^th^ day the cells are collected and RNA is extracted. SH-SY5Y were used as control cell line for the differentiation experiment (were treated with medium alone or with retinoic acid 1.25 µM and images were acquired at the 9^th^ day). **D,** Optical microscopy image for LAN-5 cell line treated for 9 days (from left to right, untreated, 2.5 μM RA, 2.5 μM BGA002, 2.5 μM BGA002+RA). Two biological replicates for experiment. **E,** Box-plots represent the length of neurite in MNA-NB cell line LAN-5 after 9 days of treatment (CTRL: medium alone, RA: retinoic acid 2.5 µM, BGA002: BGA002 2.5 µM, BGA002 + RA: BGA002 2.5 µM and retinoic acid 2.5 µM). Each dot represents the measurement for a single neurite, the middle line is representing the median while the box limits indicate the first and the third quartiles and whiskers specify samples comprised 1.5 times the interquartile range. The graph represents the results of two pooled experiments. Statistic: Wilcoxon matched pair test. *p≤0.05, **p≤0.01, ***p≤0.001, ****p≤0.0001.


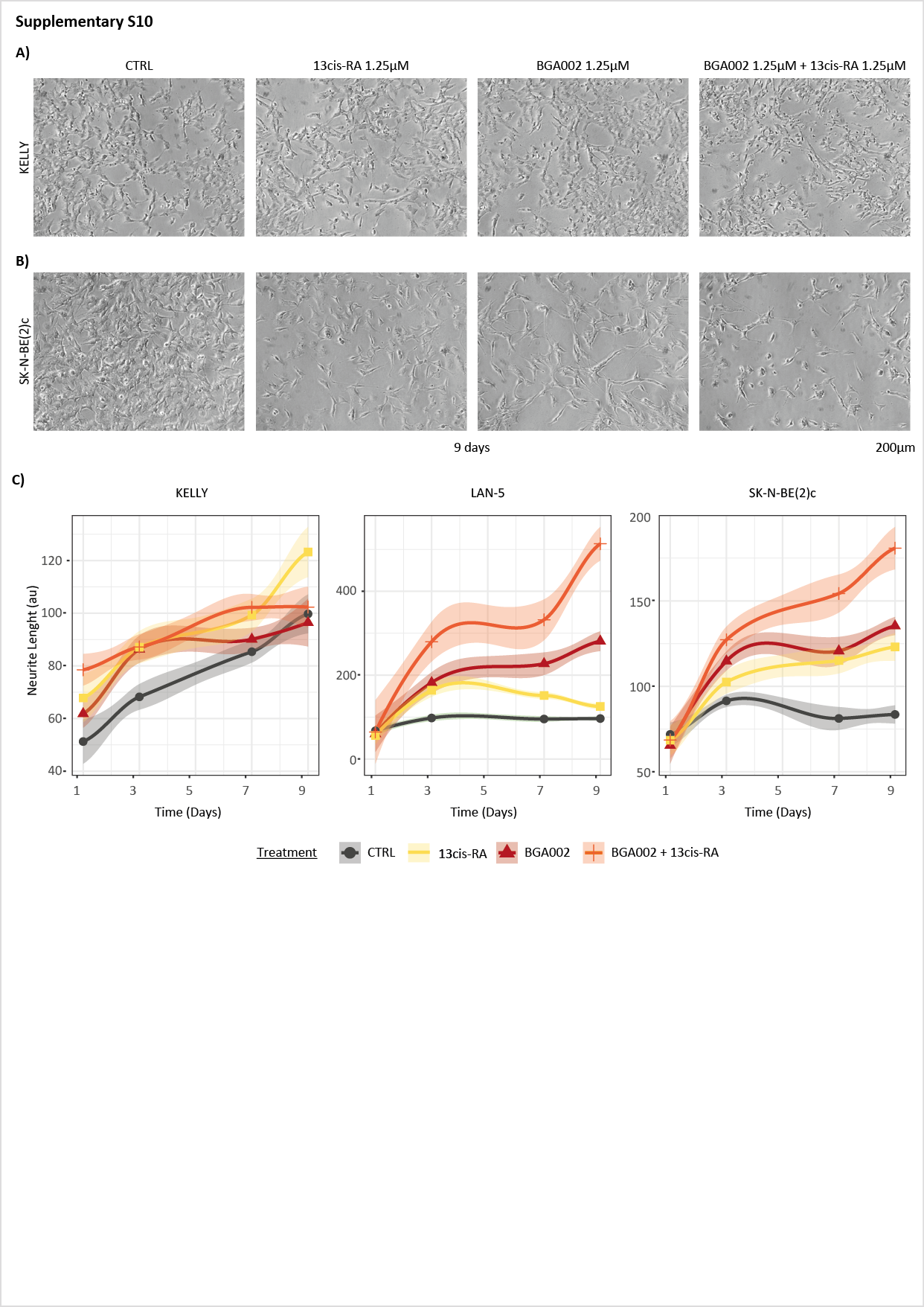


**Supplementary figure 10.**

**BGA002 in combination with RA induces differentiation in MYCN amplified cell lines**

**A,** Optical microscopy image for Kelly cell line treated for 9 days (from left to right, untreated, 1.25 μM RA, 1.25 μM BGA002, 1.25 μM BGA002+RA). Two biological replicates for experiment. **B,** Optical microscopy image for SK-N-BE(2)-c cell line treated for 9 days (from left to right, untreated, 1.25 μM RA, 1.25 μM BGA002, 1.25 μM BGA002+RA). Two biological replicates for experiment. **C,** Line plots present the neurite length measurement at different timepoints (1, 3, 5, 7, 9 days) for three different MNA neuroblastoma cell lines (Kelly, LAN-5, SK-N-BE(2)-c) treated with different treatments (CTRL: medium alone, RA: retinoic acid 1.25 µM, BGA002: BGA002 1.25 µM, BGA002 + RA: BGA002 1.25 µM and retinoic acid 1.25 µM). The line-plot represents 2 pooled different biological replicates. The curve is the results of the polynomial fitting on the data. The ribbon represents the standard error and the point the mean for each condition at each day.


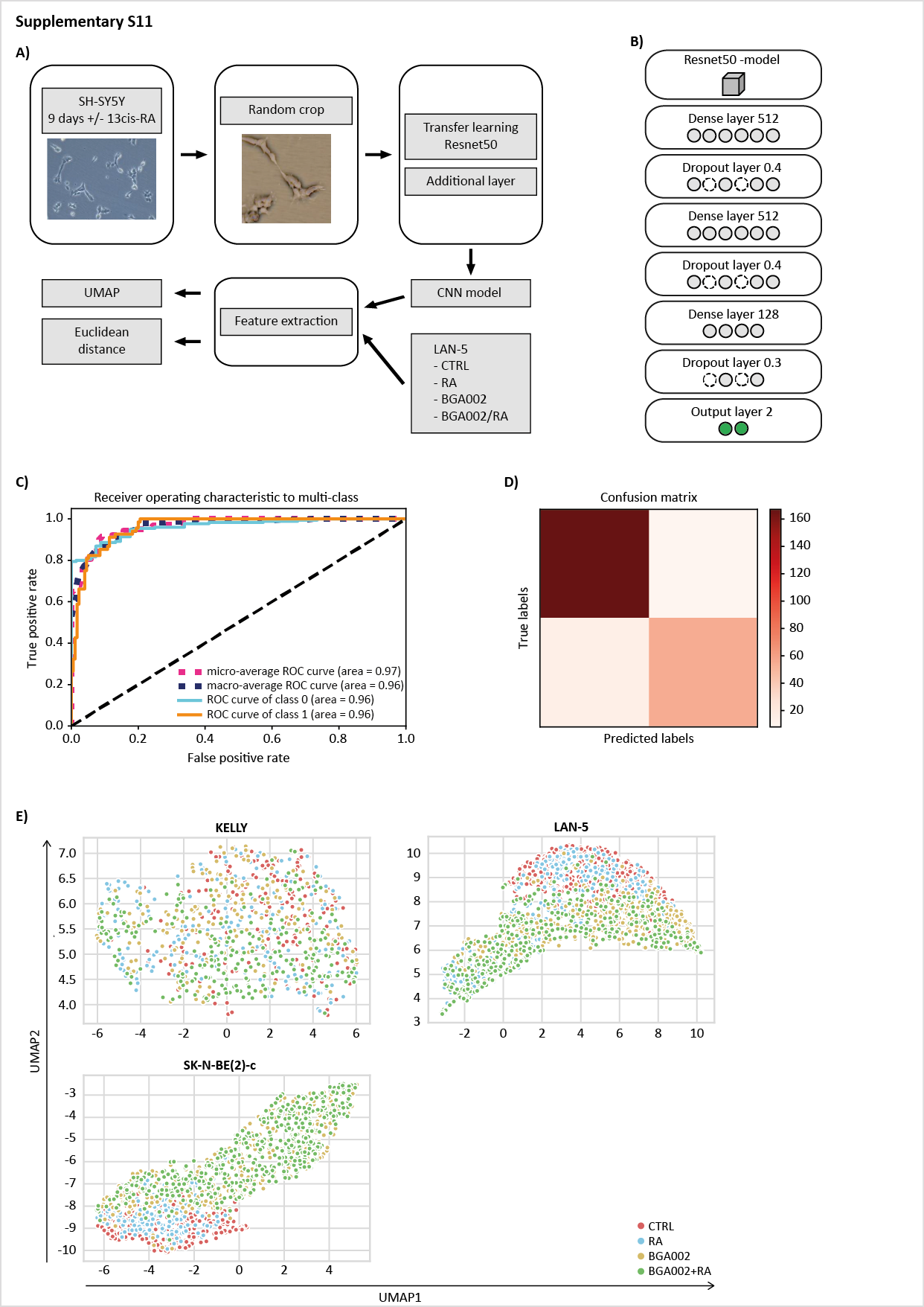


**Supplementary figure 11.**

**Neural network analysis shows that BGA002 in combination with RA is capable of inducing differentiation in MYCN amplified cell lines**

**A,** Schematic representation of the CNN differentiation pipeline. Optical microscopy acquisitions of SH-SY5Y cell line treated with retinoic acid or medium alone were taken at the 9^th^ day. Random cropping followed by sample augmentation (random zoom, random rotation, width and height shift, horizontal flip) was conducted on the image dataset. Pre-trained Resnet50 on Imagenet was downloaded and incorporated in a new CNN model. The CNN model was trained on the SH-SY5Y cell line treated images. Kelly, LAN-5, SK-N-BE(2)-c MNA cell lines treated (CTRL: medium alone, RA: retinoic acid 1.25 µM, BGA002: BGA002 1.25 µM, BGA002 + RA: BGA002 1.25 µM and retinoic acid 1.25 µM) were pre-processed as SH-SY5Y dataset. The trained CNN model was used as feature extractor for the MNA cell lines images and on the obtained vectors UMAP and Euclidean distance calculation were conducted. **B,** Schematic representation of the CNN differentiation model. **C,** Receiver Operating Characteristic (ROC) curve for the CNN differentiation model (class 0 represents SH-SY5Y medium control, class 1 represents SH-SY5Y cell line treated with retinoic acid). **D,** Confusion matrix for the prediction on the test set. **E,** UMAP projection for Kelly, LAN-5, SK-N-BE(2)-c MNA cell lines after 9 days of treatment (CTRL: medium alone, RA: retinoic acid 1.25 µM, BGA002: BGA002 1.25 µM, BGA002 + RA: BGA002 1.25 µM and retinoic acid 1.25 µM).


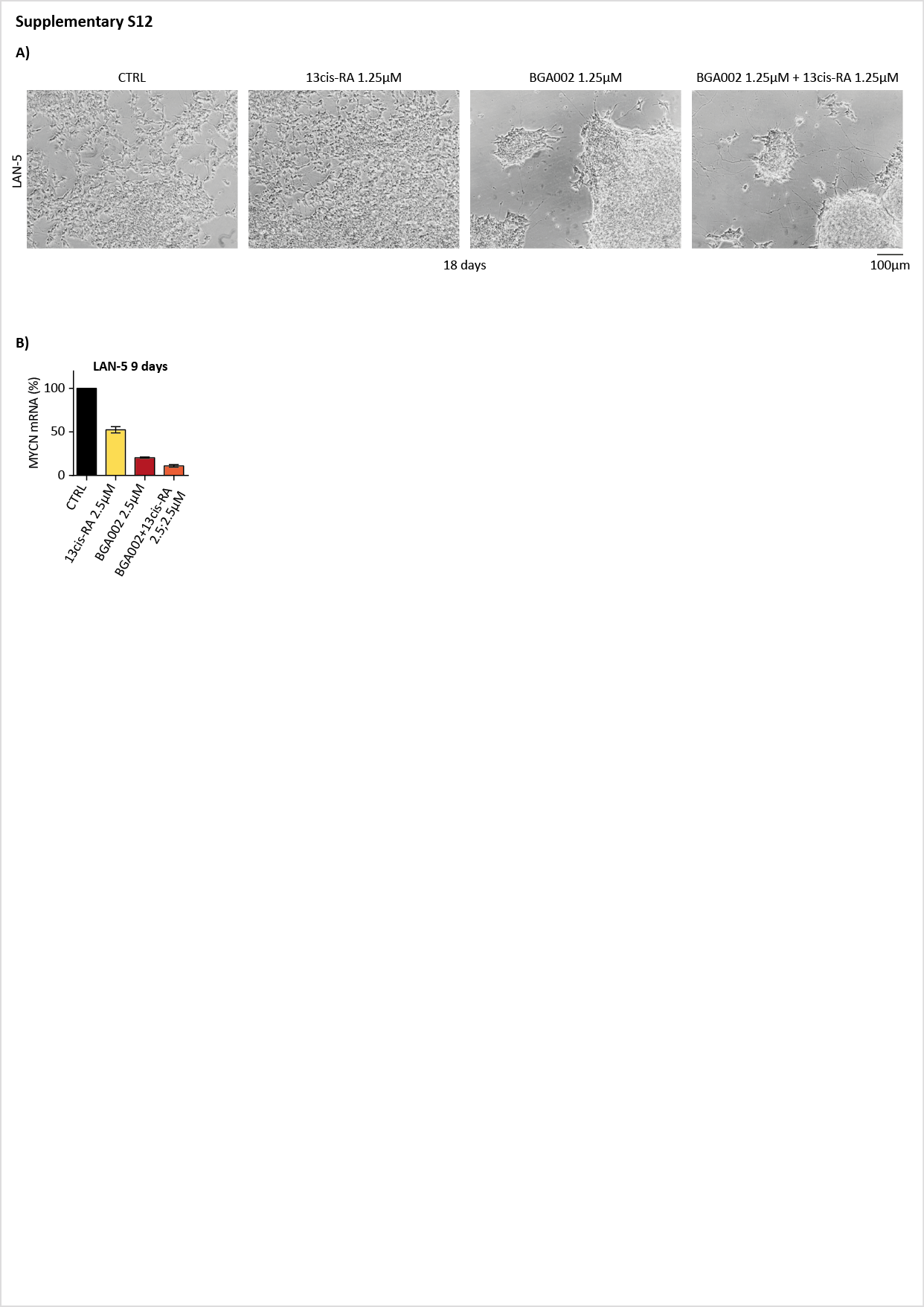


**Supplementary figure 12.**

**BGA002-RA MNA NB treated cell lines maintain differentiated phenotype after end of treatment**

**A,** Optical microscopy image for LAN-5 cell line treated for 9 days (from left to right, untreated, 1.25 μM RA, 1.25 μM BGA002, 1.25 μM BGA002+RA). Image acquisition were conducted at 18^th^ day (9 days after the end of the drug treatment). Two biological replicates for experiment. **B,** MYCN mRNA expression normalized over the control (n = 3 experiment for cell line). The bar represents the mean, the whisker the standard deviation. MYCN mRNA expression in LAN-5 (untreated, 2.5 μM RA, 2.5 μM BGA002, 2.5 μM BGA002+RA) 9 days post treatment.


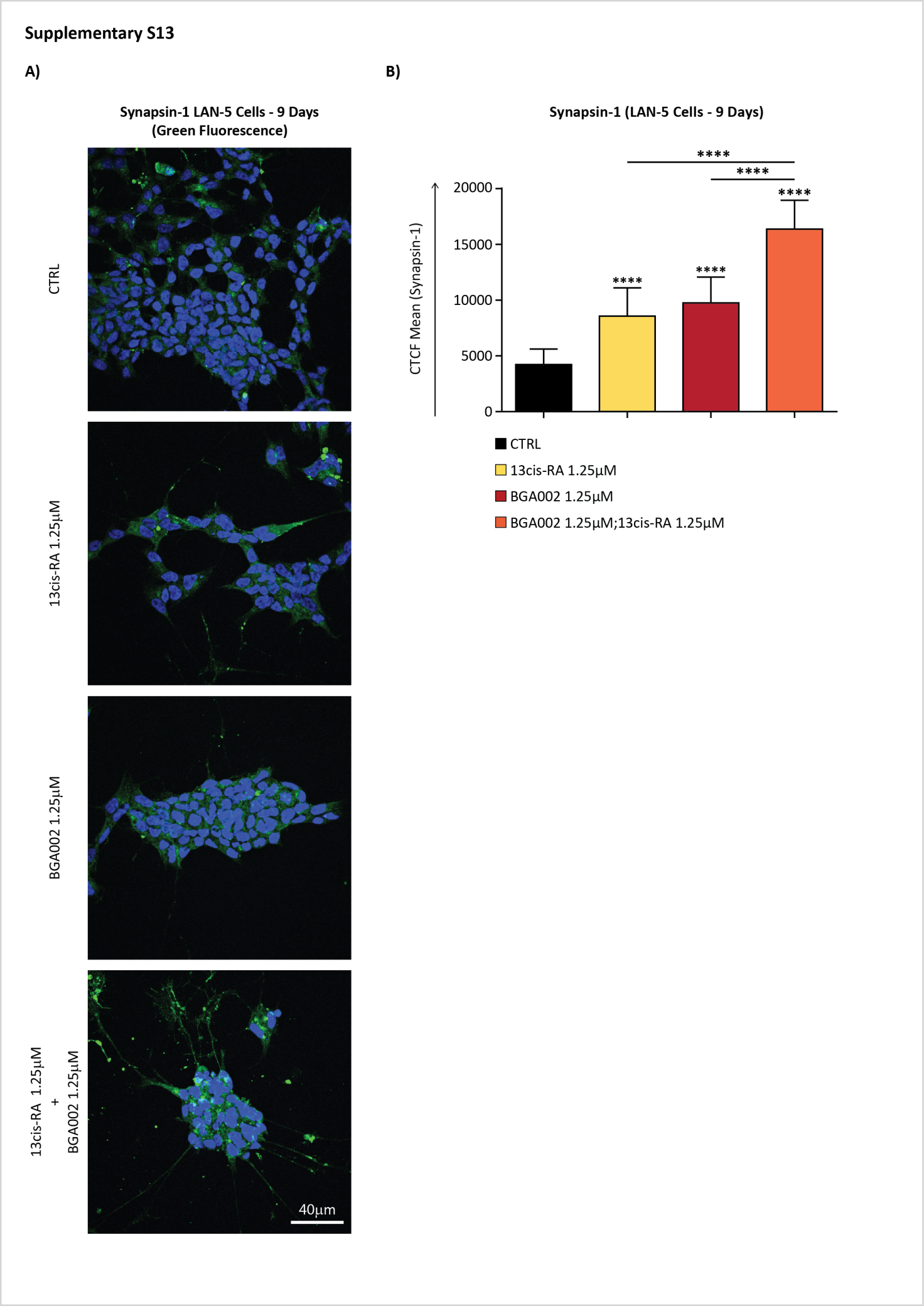


**Supplementary Figure 13**

**Synapsine-1 neuronal differentiation marker is more expressed in LAN-5 cell line after combination treatment.**

**A,** Fluorescence confocal images (60x magnification) acquired after treatment of LAN-5 with BGA002, 13-cis RA or combination at 1.25 µM for 9 days. Combination treatment shows higher synapsin-1 signal (in green) and the most prominent neuron like morphology, with clustered cells and neuritis like cell cytoplasm projection. **B,** Synapsin-1 fluorescence signal is quantified using CTCF methods, normalized on cells number. Fluorescence signals are lower in the control group then treatment groups (two-side unpaired t-test ****p<0.001). Combination treatment shows the highest fluorescence intensity compared with both BGA002 and 13-cis RA single treatment and control as well (two-side unpaired t-test ****p<0.001). Fluorescence intensity is expressed as CTCF units.


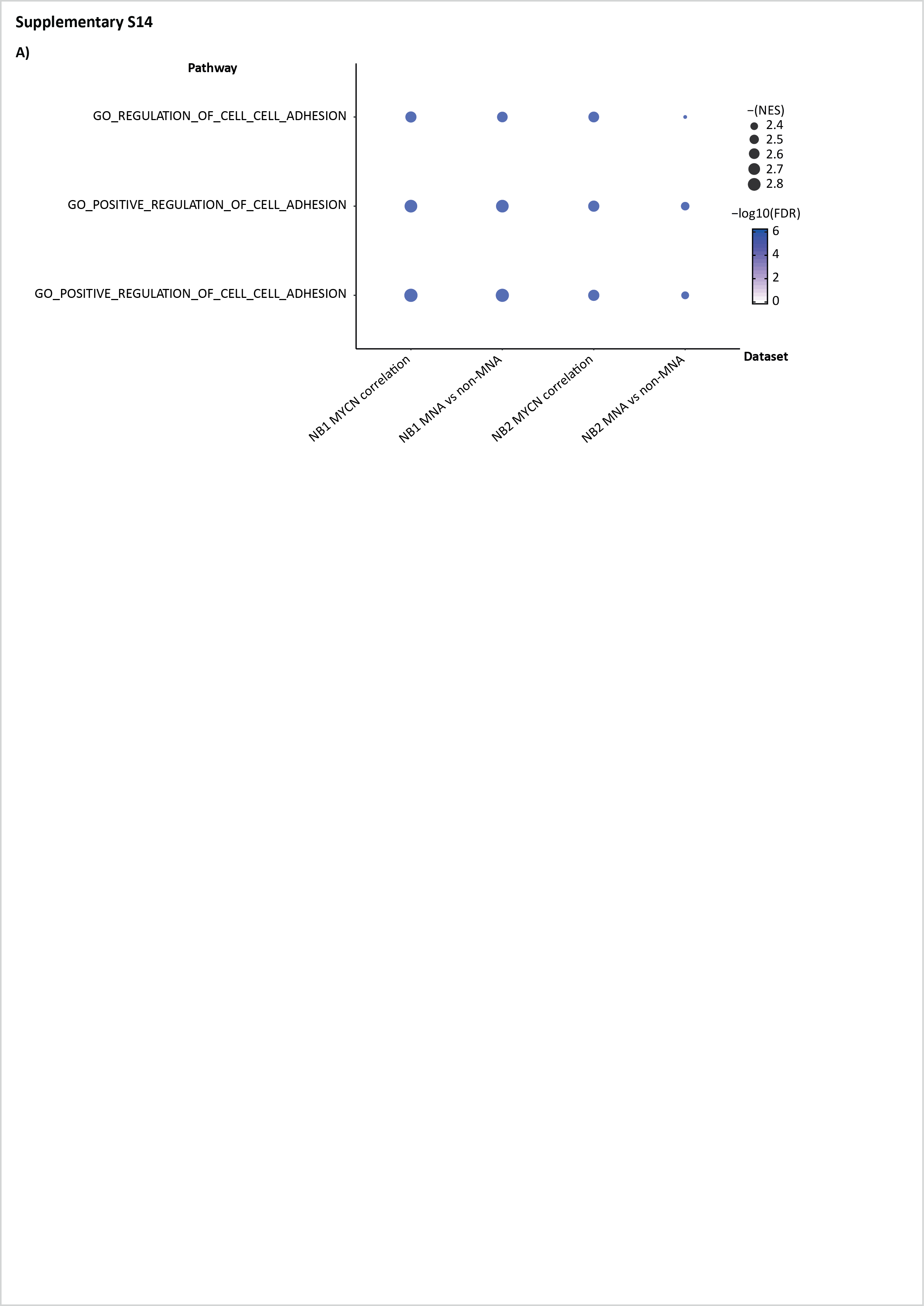


**Supplementary figure 14.**

**Cell-cell adhesion pathways are downregulated in MYCN amplified patients**

**A,** Pathway enrichment (Gene Ontology (GO) terms) for 3 selected pathways involved in cell adhesion in two different neuroblastoma datasets: NB1 cohort (E-MTAB-1781) and NB2 cohort (TARGET). Symbol size indicates the negative logarithm (base 10) of the false discovery rate (FDR) while color scale indicates the NES. From left to right, GO enrichment in NB1 correlated genes, NB1 MNA versus not-MNA differential expressed genes (DEG), NB2 correlated genes, NB2 MNA versus not-MNA differential expressed genes (DEG). **B,** Example of GSEA plot for GO POSITIVE REGULATION OF CELL-CELL ADHESION for E-MTAB-1781 MYCN negative correlated genes, TARGET MYCN negative correlated genes, E-MTAB-1781 non-MNA gene expression profiles and TARGET non-MNA gene expression profiles.


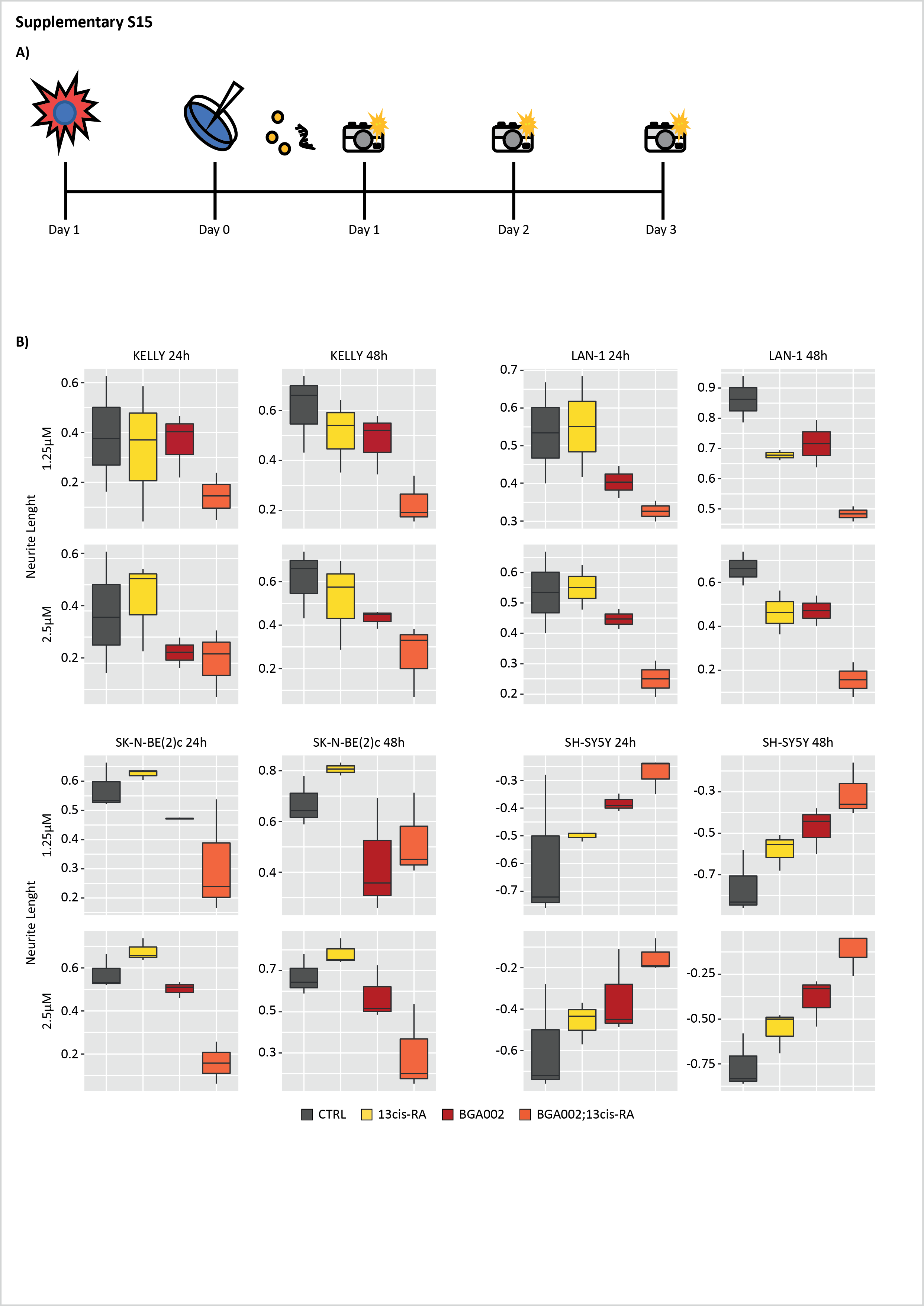


**Supplementary figure 15.**

**BGA002-RA inhibition migration ability of MNA NB cell lines**

**A,** Schematic representation of the *in vitro* NB cell lines treatment. Kelly, LAN-5, SK-N-BE(2)-c and LAN-1 are seeded the day before to start the treatment and then the day after (day 0) treated with different treatments. CTRL: medium alone, RA: retinoic acid, BGA002: BGA002, BGA002 + RA: BGA002 and retinoic acid. The treatments were performed at 1.25 µM or 2.5 µM. The scratch is performed at day 0, then every day optical images are acquired and the scratch are measured at each time point. **B,** Bar plot representing the wound healing closure normalized over the control after 24 and 48 hours for 4 different NB cell lines (n = 3 different biological replicates). Middle line, the mean; whisker, the standard deviation. First line, from left to right: Kelly cell line treated with 1.25 μM at 24 hours, Kelly cell line treated with 1.25 μM at 48 hours, LAN-1 cell line treated with 1.25 μM at 24 hours, LAN-1 cell line treated with 1.25 μM at 48 hours. Second line, from left to right: Kelly cell line treated with 2.5 μM at 24 hours, Kelly cell line treated with 2.5 μM at 48 hours, LAN-1 cell line treated with 2.5 μM at 24 hours, LAN-1 cell line treated with 2.5 μM at 48 hours. Third line, from left to right: SK-N-BE(2)c cell line treated with 1.25 μM at 24 hours, SK-N-BE(2)c cell line treated with 1.25 μM at 48 hours, SH-SY5Y cell line treated with 1.25 μM at 24 hours, SH-SY5Y cell line treated with 1.25 μM at 48 hours. Fourth line, from left to right: SK-N-BE(2)c cell line treated with 2.5 μM at 24 hours, SK-N-BE(2)c cell line treated with 2.5 μM at 48 hours, SH-SY5Y cell line treated with 2.5 μM at 24 hours, SH-SY5Y cell line treated with 2.5 μM at 48 hours.


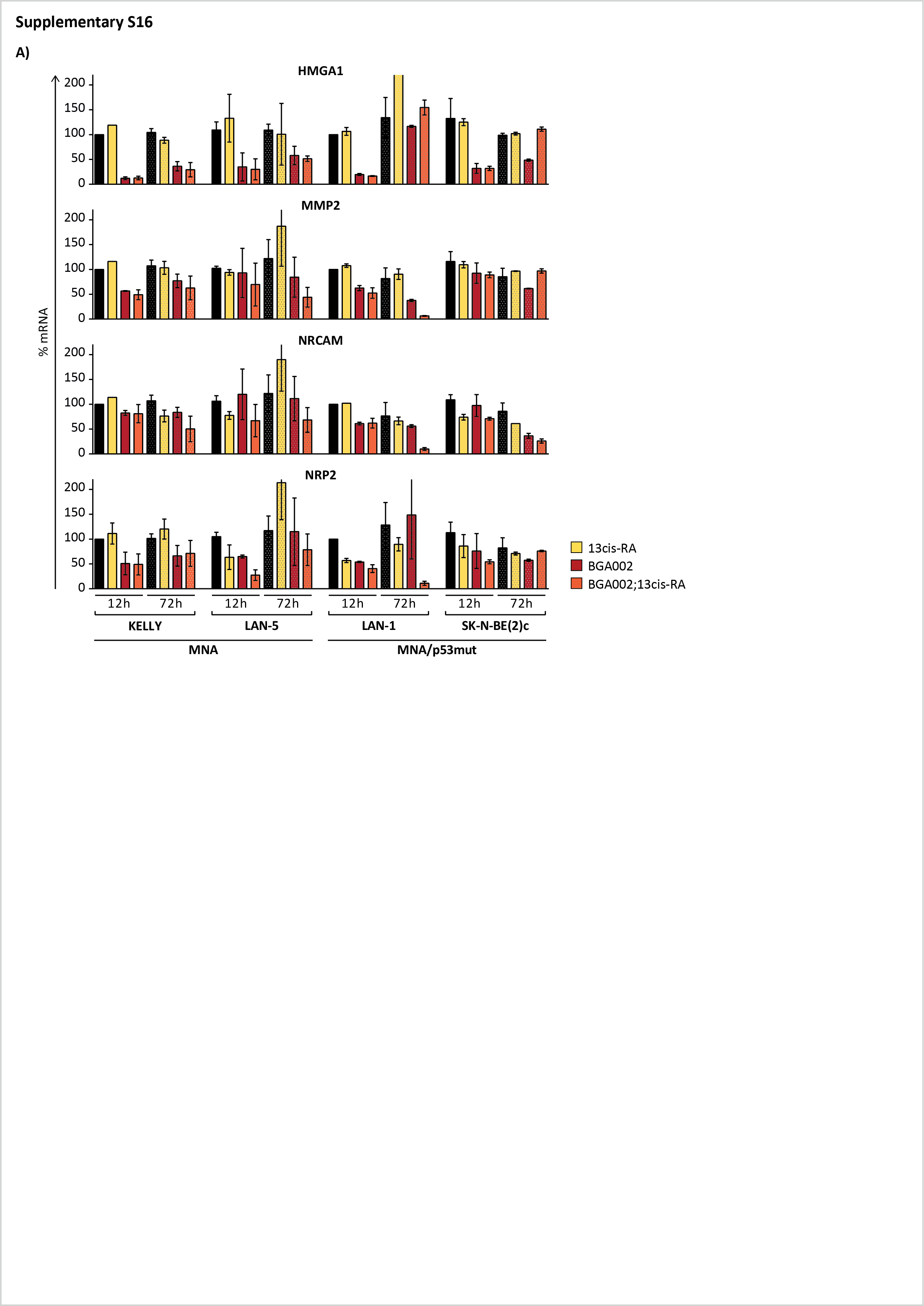


**Supplementary figure 16.**

**BGA002-RA inhibition migration ability of MNA NB cell lines**

**A,** mRNA expression normalized over the control (n = 3 experiment for cell line). The bar represents the mean, the whisker the standard deviation. From top to down, mRNA expression for HMGA1, MMP2, NRCAM and NRP2. From left to right, Kelly, LAN-5, LAN-1 and SK-N-BE(2)-c after 12 and 72 hours of treatment (CTRL: medium alone, RA: retinoic acid 1.25 µM, BGA002: BGA002 1.25 µM, BGA002 + RA: BGA002 1.25 µM and retinoic acid 1.25 µM). Cell lines are ordered according their MYCN amplification and p53 mutation status.


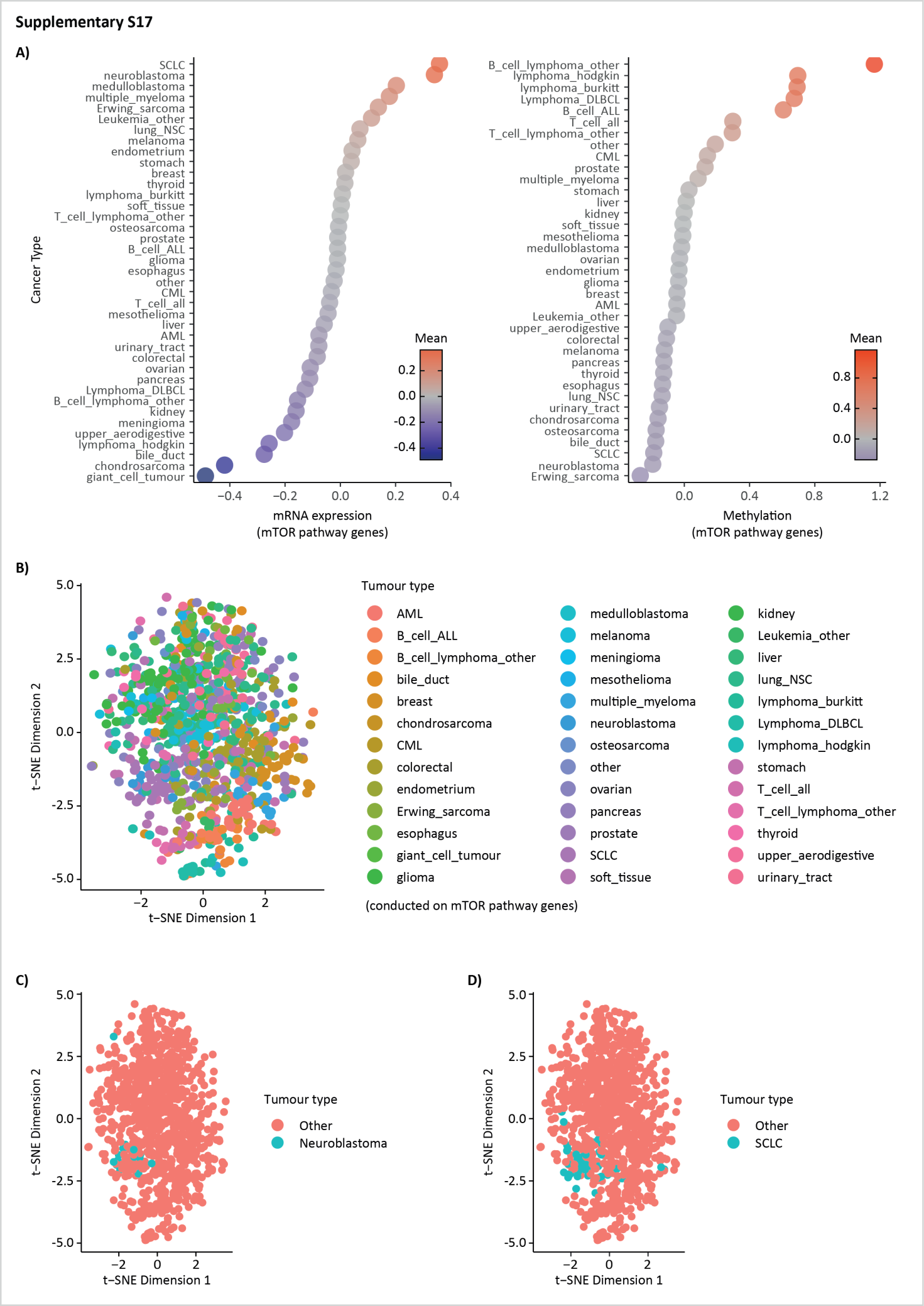


**Supplementary figure 17.**

**Neuroblastoma cell lines are enriched for a mTOR pathway signature**

**A,** Dot-plots representing the normalized mean of the mRNA expression (left panel) and the normalized mean of the methylation level (right plot) for the genes in the mTOR pathway signature in cancer cell lines. Every dot represents the normalized mean value for a cancer type as listed in Cancer Cell Line Encyclopaedia (CCLE). **B,** t-SNE on the mRNA expression of the mTOR pathway signature in cancer cell lines. Every dot represents a gene expression profile for a cell line, coloured differently for each cancer type as listed in CCLE. **C,** t-SNE on the mRNA expression of the mTOR pathway signature in cancer cell lines. Neuroblastoma cell lines are highlighted in blue. **D,** t-SNE on the mRNA expression of the mTOR pathway signature in cancer cell lines. Small lung cancer cell (SCLC) cell lines are highlighted in blue.


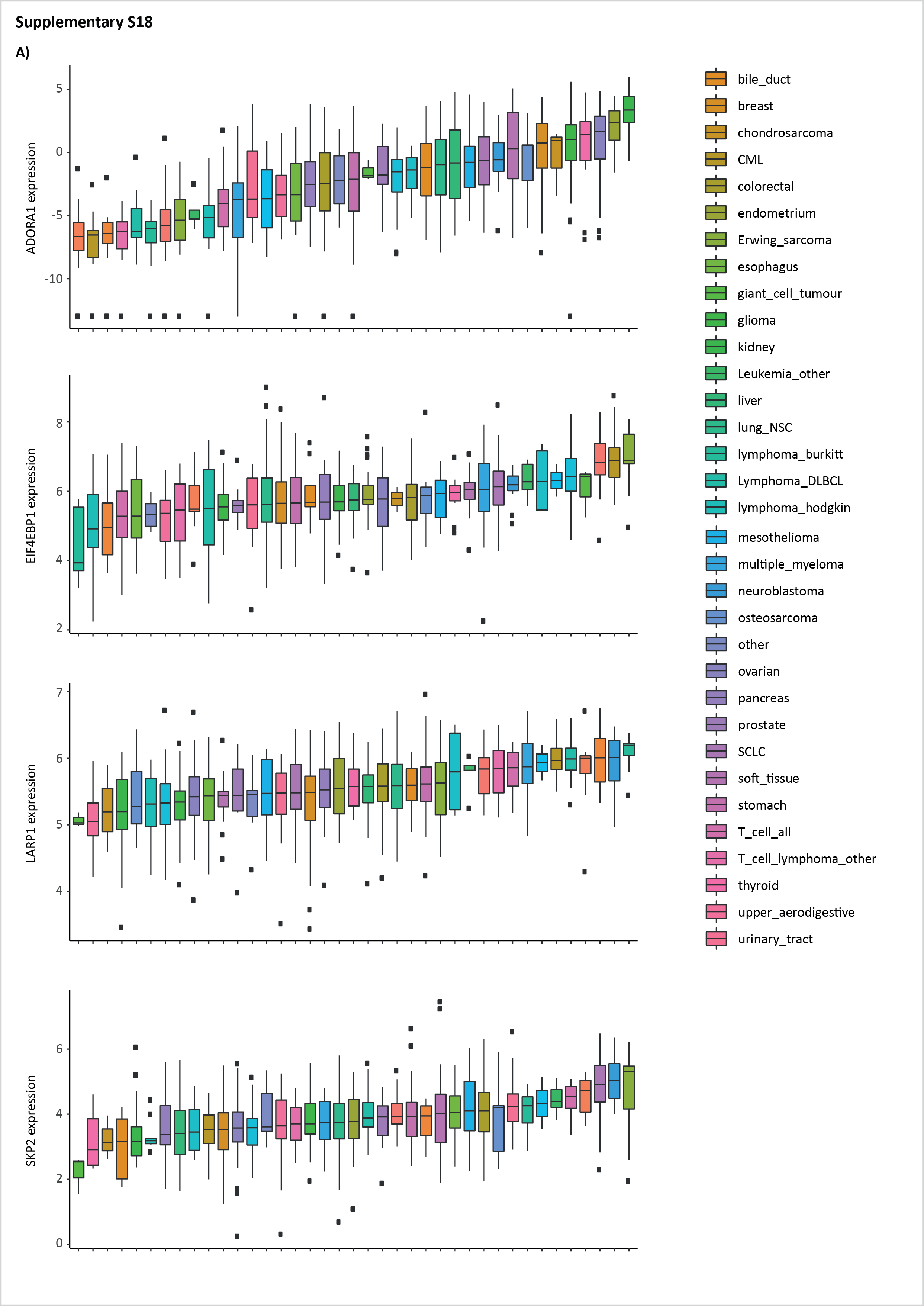


**Supplementary figure 18.**

**Neuroblastoma cell lines are enriched for a mTOR pathway signature**

**A,** Box-plot represents the mRNA expression for some of the genes present in the MTOR pathway signature. Each box-plot represents the mRNA expression for a cancer type as listed Cancer Cell Line Encyclopaedia (CCLE). Each dot represents the mRNA expression for a single cell line, the middle line is representing the median while the box limits indicate the first and the third quartiles and whiskers specify samples comprised 1.5 times the interquartile range. From top to down: ADORA1, EIF4BP1, LARP1, SKP2 mRNA expression.


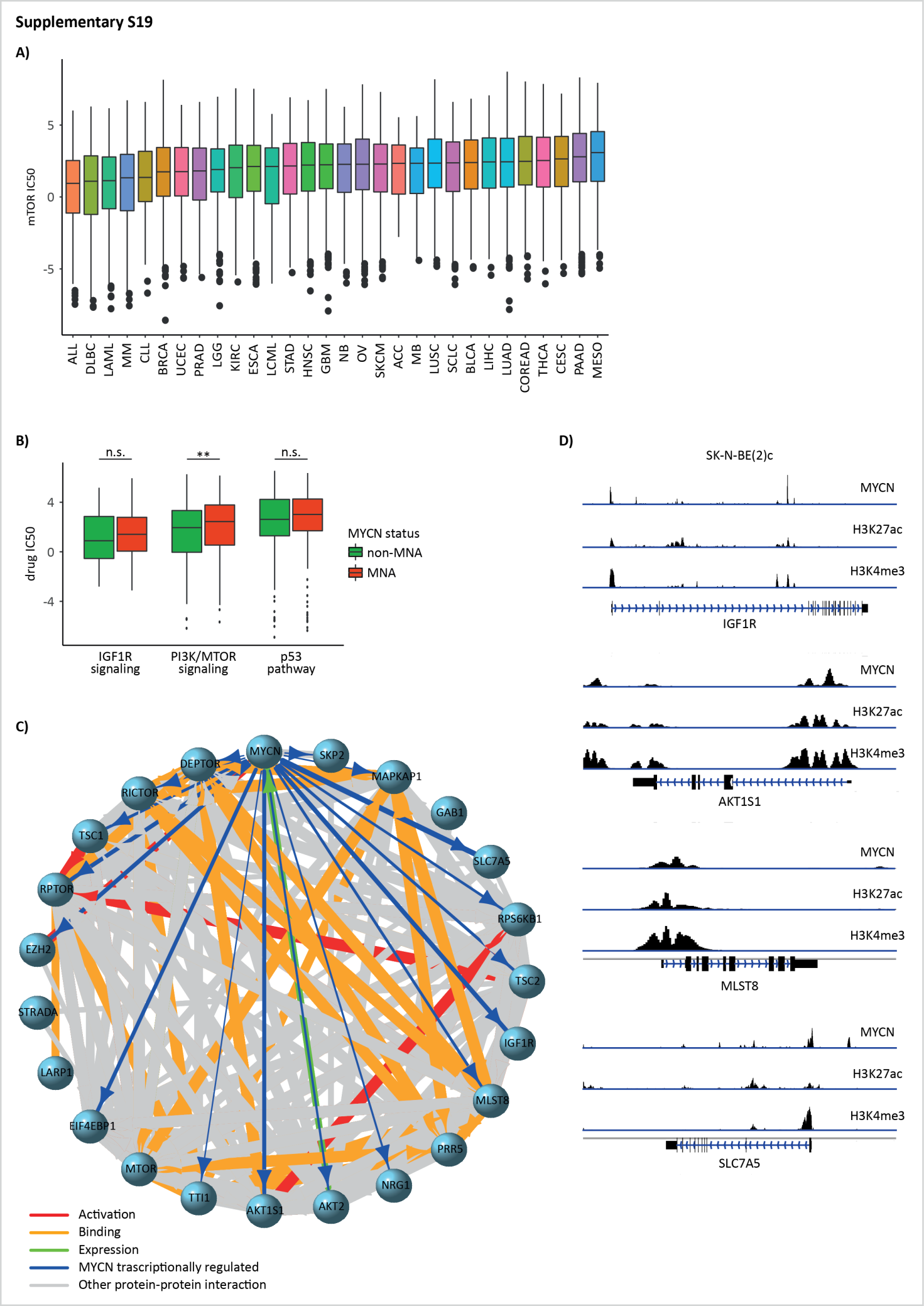


**Supplementary figure 19.**

**Neuroblastoma MNA cell lines are resistant to mTOR inhibition**

**A,** Box-plots represent half maximal growth inhibition concentration (GI_50_) in Sanger database cell line. Each boxplot represents a cancer type as listed in the database (ACC: Adrenocortical carcinoma, ALL: Acute lymphoblastic leukemia, BLCA: Bladder Urothelial Carcinoma, BRCA: Breast invasive carcinoma, CESC: Cervical squamous cell carcinoma and endocervical adenocarcinoma, CLL: lymphoid neoplasm other, COREAD: Colon adenocarcinoma, DLBC: Lymphoid Neoplasm Diffuse Large B-cell Lymphoma,ESCA: Esophageal carcinoma, GBM: Glioblastoma multiforme, HNSC: Head and Neck squamous cell carcinoma, KIRC: Kidney renal clear cell carcinoma, LAML: Acute Myeloid Leukemia, LCML: Chronic Myelogenous Leukemia, LIHC: Liver hepatocellular carcinoma, LUAD: Lung adenocarcinoma, LUSC: Lung squamous cell carcinoma, MESO: Mesothelioma, MB: Medulloblastoma, NB: Neuroblastoma, OV: Ovarian serous cystadenocarcinoma, PAAD: Pancreatic adenocarcinoma, PRAD: Prostate adenocarcinoma, SCLC: Small Cell Lung Cancer, SKCM: Skin Cutaneous Melanoma, STAD: Stomach adenocarcinoma, THCA:Thyroid carcinoma, UCEC: Uterine Corpus Endometrial Carcinoma). Each dot represents the GI_50_ for a single cell line, the middle line is representing the median while the box limits indicate the first and the third quartiles and whiskers specify samples comprised 1.5 times the interquartile range. **B,** Box-plots represent GI_50_for IGFR1 signaling, PI3K/mTOR signaling and p53 pathway inhibition in neuroblastoma cell line present in Sanger database cell line. MNA and not-MNA cell line are listed as different box-plot. Each dot represents the GI_50_ for a single cell line, the middle line is representing the median while the box limits indicate the first and the third quartiles and whiskers specify samples comprised 1.5 times the interquartile range. Statistics are indicated over the boxplots, Wilcoxon matched pair test. **C,** Protein-protein interaction between the genes in the mTOR pathway signature. The different colours indicate different type of protein-protein interaction as listed in the STRING database, blue lines indicate transcriptional regulation by N-Myc. Grey colour lines represent un-specified protein-protein interaction. **D,** ChIP-seq peaks for H3K4me3, H3K27ac and N-MYC occupancy in SK-N-BE(2)c (GSE80151). From top to down: IGF1R, AKT1S1, MLST8, SLC7A5. **p≤0.01,****p≤0.0001.


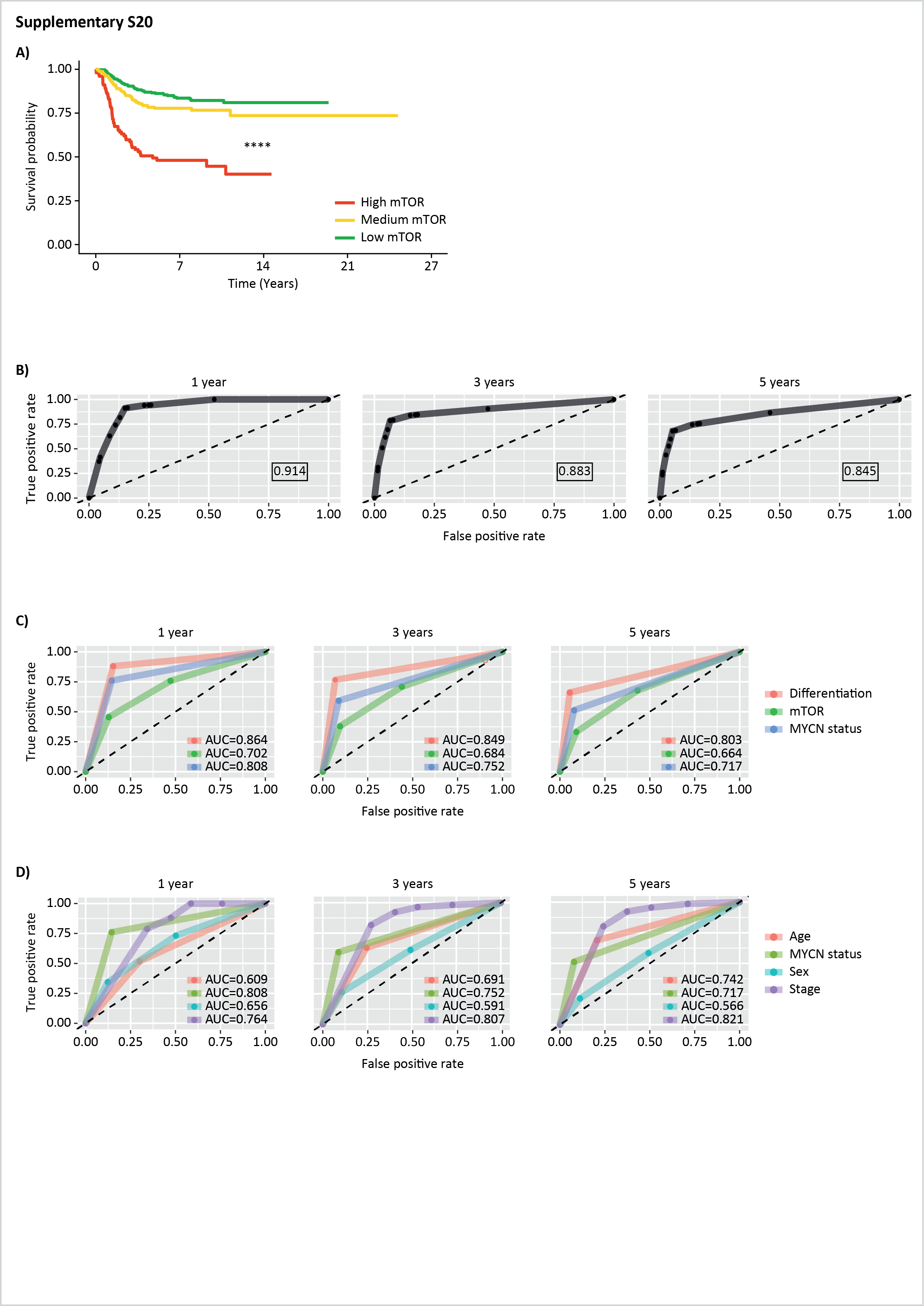


**Supplementary figure 20.**

**mTOR signature is predictive for survival in NB patients**

**A,** Kaplan-Meier curve for the probability of overall survival over time for mTOR genes pathway expression in NB1 cohort (E-MTAB-1781; High mTOR expression, n = 100; Medium mTOR expression, n = 361; Low mTOR expression, n = 240). Associated p value is shown in the middle of the plot (log-rank test). **B,** Receiver Operating Characteristic (ROC) and Area Under the Curve (AUC) for combined score (mTOR risk signature, differentiation risk signature and MYCN status). True positive rate (TPR) on the vertical axis and false positive rate (FPR) on the horizontal axis. AUC is listed in the middle. From left to right ROC and AUC for: 1 year (360 days), 3 years (1080 days) and 5 years (1800 days). **C,** Receiver Operating Characteristic (ROC) and Area Under the Curve (AUC) for mTOR risk signature, differentiation risk signature and MYCN status separately. True positive rate (TPR) on the vertical axis and false positive rate (FPR) on the horizontal axis. AUC is listed in the middle. From left to right ROC and AUC for: 1 year (360 days), 3 years (1080 days) and 5 years (1800 days). **D,** Receiver Operating Characteristic (ROC) and Area Under the Curve (AUC) for Age, MYCN status, Sex and Stage separately. True positive rate (TPR) on the vertical axis and false positive rate (FPR) on the horizontal axis. AUC is listed in the middle. From left to right ROC and AUC for: 1 year (360 days), 3 years (1080 days) and 5 years (1800 days).


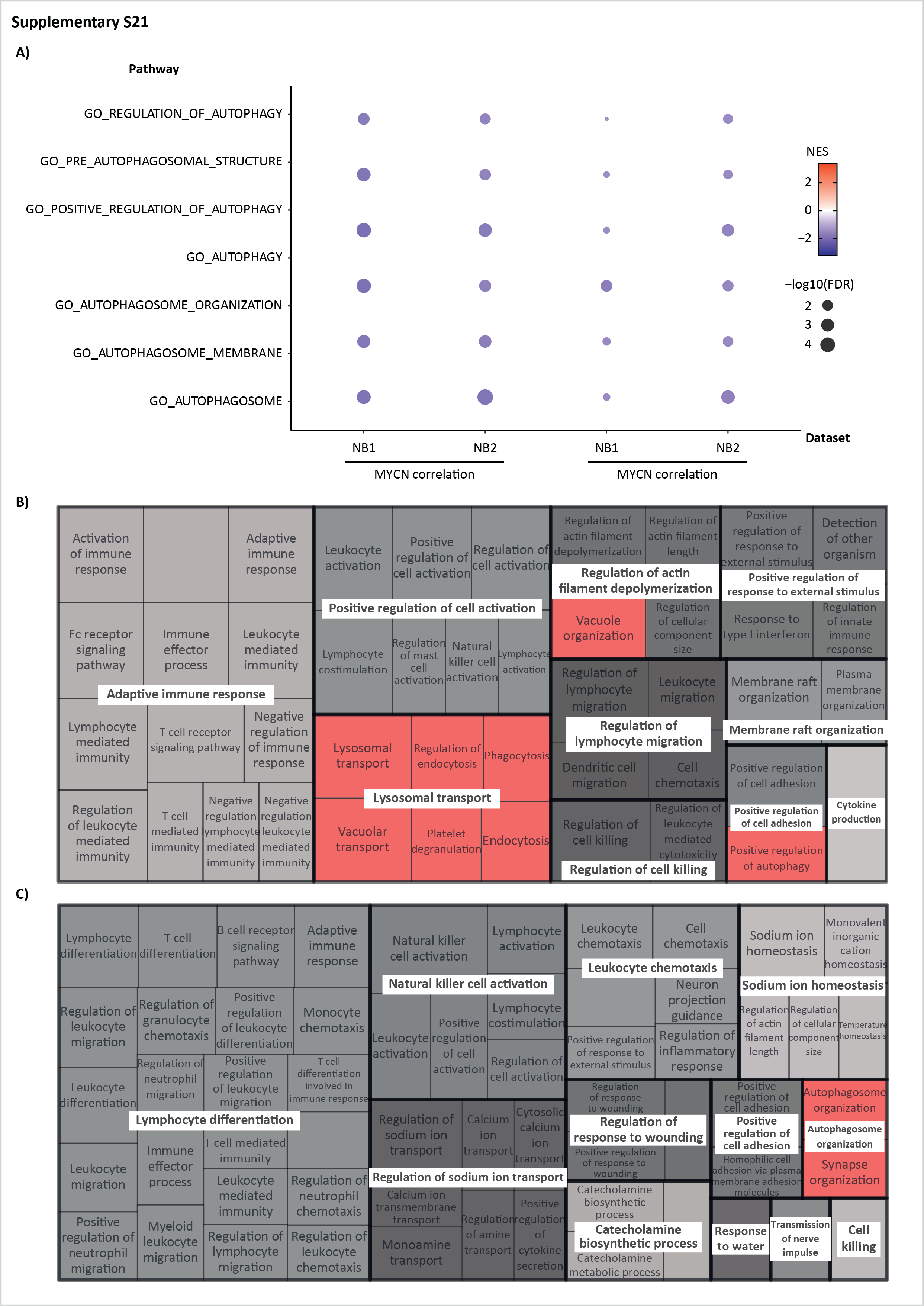


**Supplementary figure 21.**

**MYCN is associate with autophagy down-regulation**

**A, P**athway enrichment (Gene Ontology (GO) terms) for 6 selected pathways involved in autophagy in two different neuroblastoma datasets: NB1 cohort (E-MTAB-1781) and NB2 cohort (TARGET). Symbol size indicates the negative logarithm (base 10) of the false discovery rate (FDR) while color scale indicates the NES. From left to right, GO enrichment in NB1 MYCN correlated genes, NB2 MYCN correlated genes, NB1 MNA versus not-MNA differential expressed genes (DEG), NB2 MNA versus not-MNA differential expressed genes (DEG). **B-C,** Treemap plot representing a hierarchical structure the first 100 plots, GO terms (Biological Process) are grouped based on their parent terms. The space for each term is proportional to the negative logarithm (base 10) of the false discovery rate (FDR) of the pathway. **B,** First 100 GO negatively enriched pathway in MYCN correlated genes in NB1 cohorts (E-MTAB-1781). **C,** First 100 GO negatively enriched pathway in MNA versus not-MNA differential expressed genes (DEG) in NB1 cohorts.


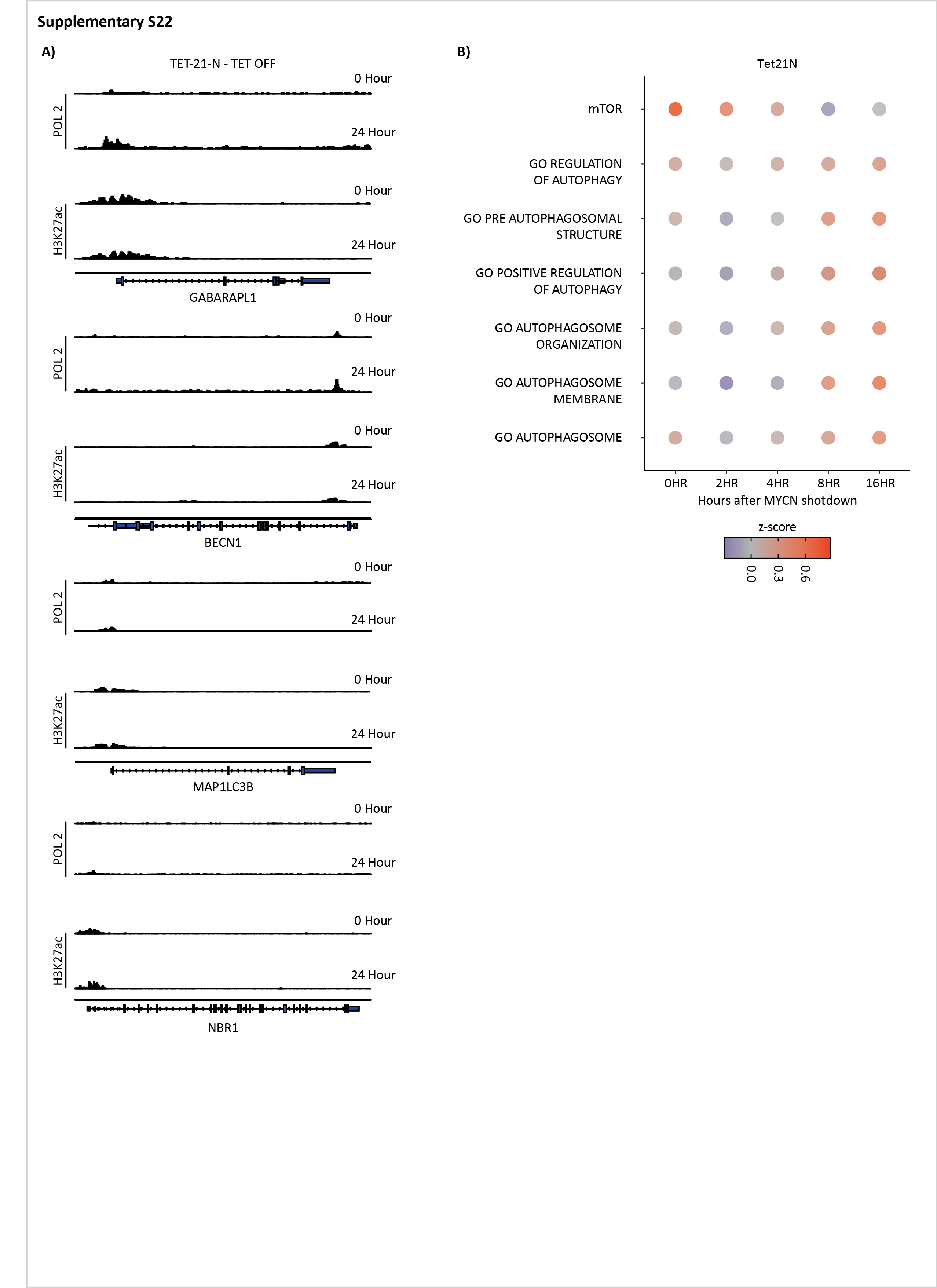


**Supplementary figure 22.**

**MYCN silencing is re-activating autophagy**

**A,** ChIP-seq peaks for POL2 and H3K27ac occupancy in Tet21N (GSE80151) after MYCN shutdown (at 0 hour and 24 hours after treatment). From top to down: GABARAPL1, BECN1, MAP1LC3B, NBR1. **B,** Signature enrichment for mTOR 6 selected pathways involved in autophagy (Gene Ontology (GO) terms) in Tet21N after MYCN shutdown at different timepoints: gene expression profiles (GSE80153). Color scale indicates the mean of z-score for each gene in each signature.


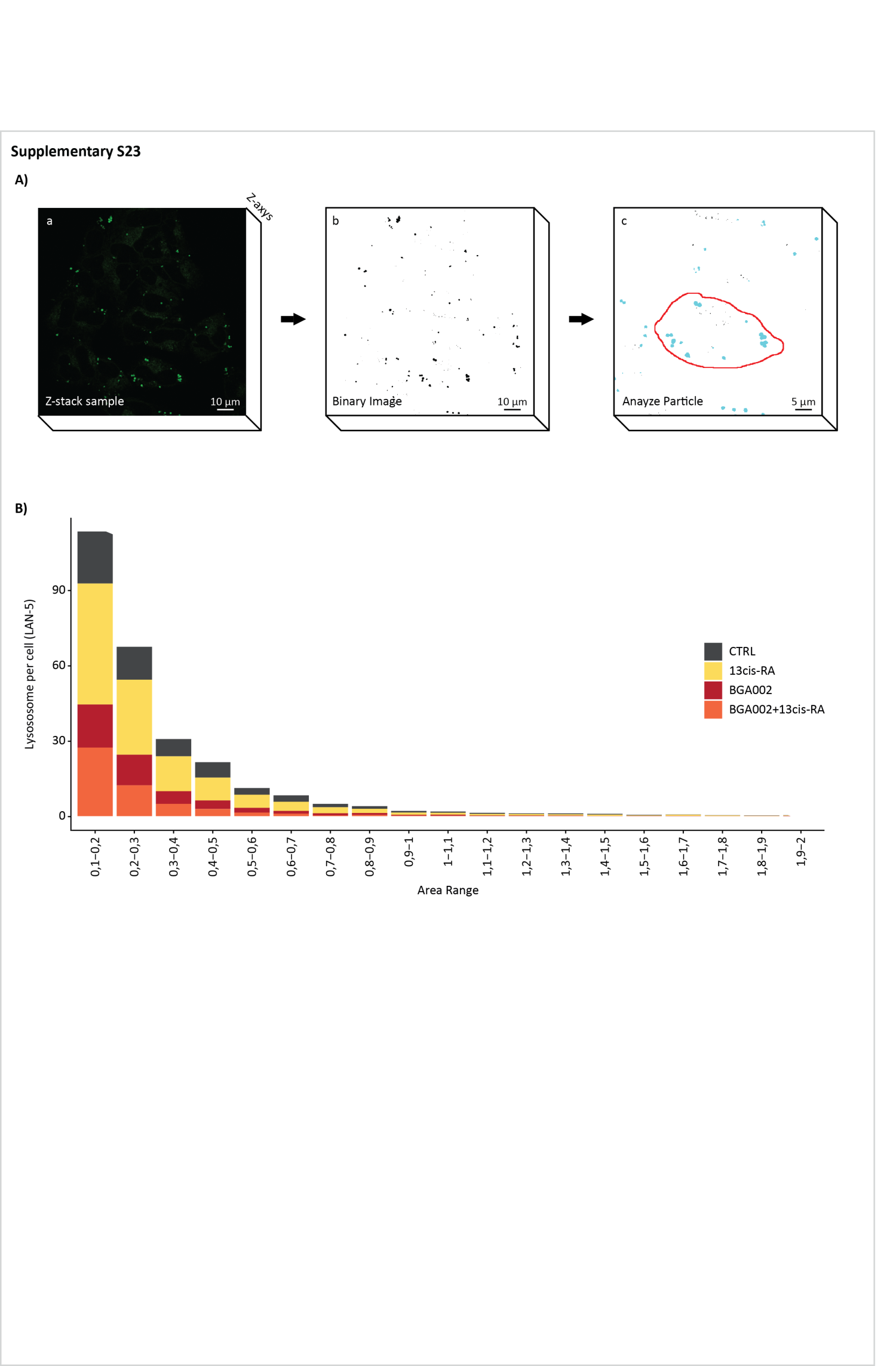


**Supplementary figure 23.**

**BGA002-RA treatment leads to autophagy reactivation.**

**A,** Example of analysis workflow for lysosome identification and analysis. **B,** Stacked bar-plot, vertical axis representing the number of lysosomes per cell after 24 hours of treatment in LAN-5 MNA cell line, horizonal axis the area range of the lysosomes. Color represents treatment (control, 13-cis RA, BGA002, BGA002+RA)

**
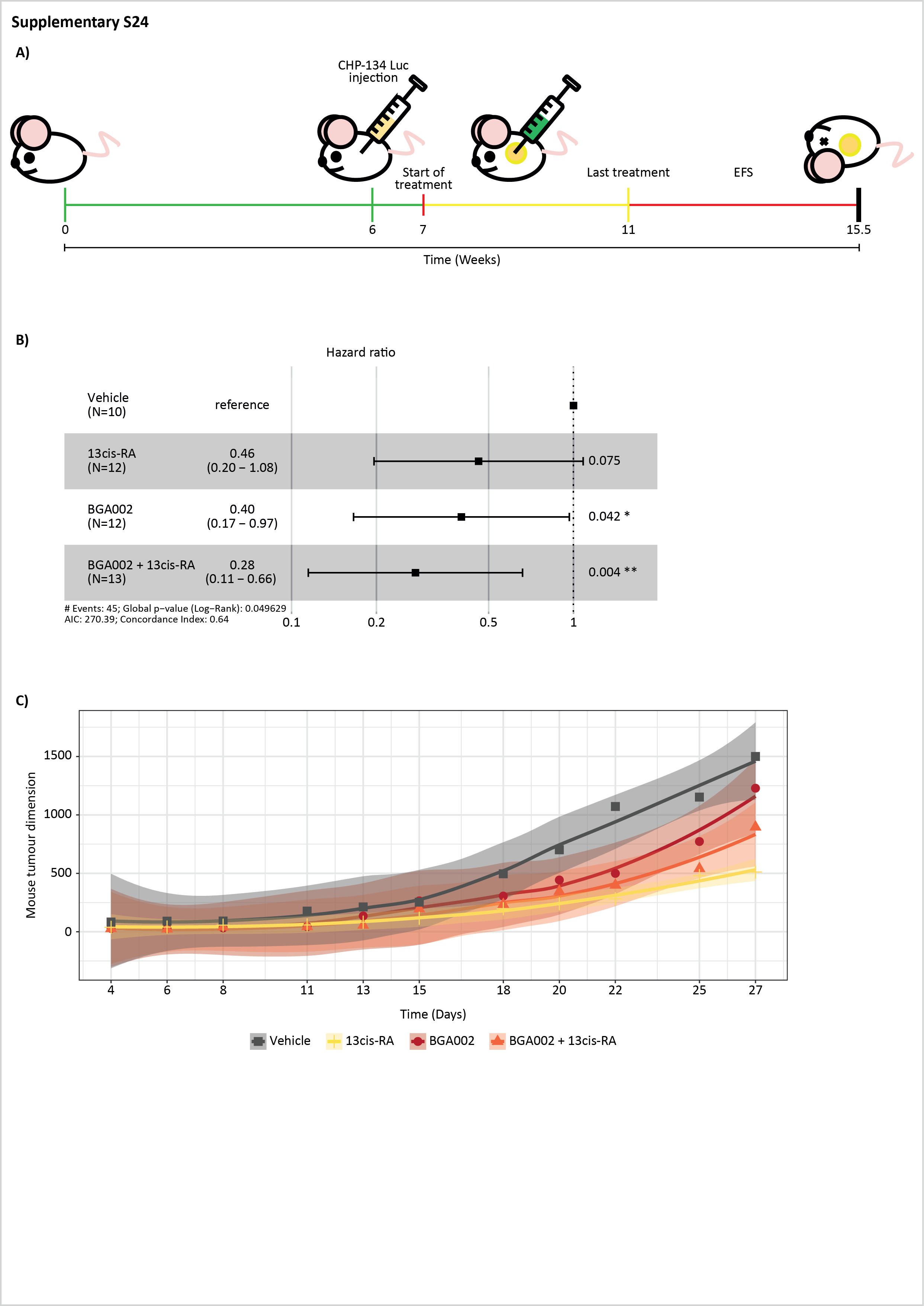
**

**Supplementary figure 24.**

**BGA002 in combination with retinoic acid is slowing tumor growth**

**A,** Schematic representation of the *in vivo* protocol. 6-week-old mice (NOD/SCID CB17, from both sexes) were inoculated with 10x10^6^ CHP134*-luc* cells. The tumour growth was monitored through bioluminescence acquisition. When the tumour reached a predefined starting point, the mice were treated through dorsal injection: vehicle, BGA002 (10 mg/kg/day), retinoic acid (10 mg/kg/day), BGA002 and retinoic acid (10 mg/kg/day). Treatment was conducted daily for 28 days. The animals were monitored daily until they reached the end point (10 mm tumour diameter) or after 60 day post treatment start. After reaching the endpoint mice were sacrificed and histology analysis was performed. **B,** Forest plot presenting univariate Cox proportional hazards regression analysis for the treatment in respect to the vehicle. Confidence interval and associated p-value are showed in the figure. **C,** Tumour growth curve measured with the caliper. The different lines represent vehicle, BGA002 (10 mg/kg/day), retinoic acid (10 mg/kg/day), BGA002 and retinoic acid (10 mg/kg/day). The standard deviation is presented as shade.


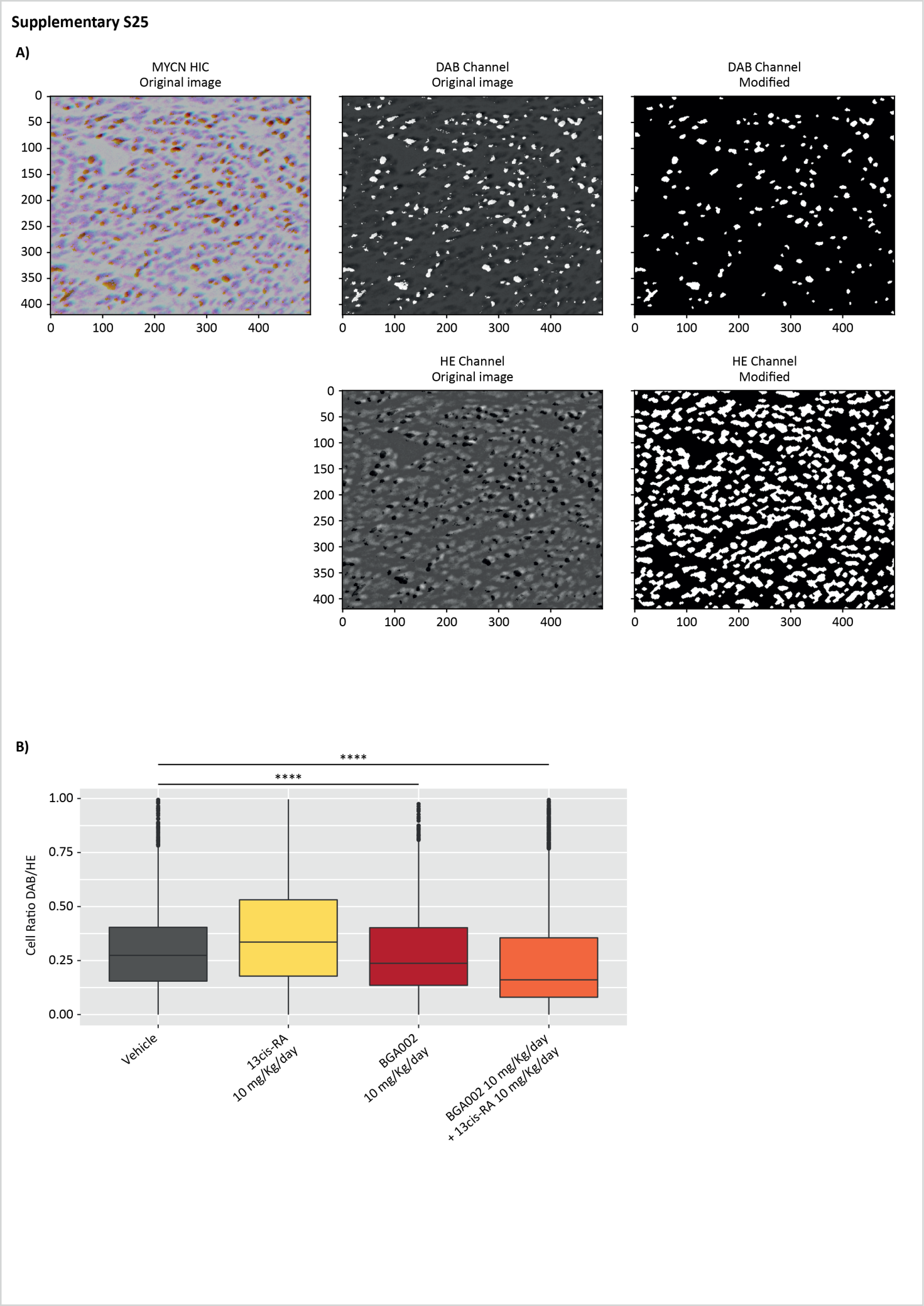


**Supplementary figure 25.**

**BGA002 in combination with retinoic acid is reducing N-Myc in the tumour**

**A,** Schematic representation of the image pre-processing before the segmentation and counting step. The images are separate in the different channels: diaminobenzidine (DAB) and hematoxylin (HE). Then through different processing steps the cell are selected, segmented and counted. DAB staining is IHC coloration for N-Myc. **B,** Box-plots representing the ratio of DAB stained/HE stained cells for vehicle, BGA002 (10 mg/kg/day), retinoic acid (10 mg/kg/day), BGA002 and retinoic acid (10 mg/kg/day each). Each dot represents the ratio calculated in one image. The middle line is representing the median while the box limits indicate the first and the third quartiles and whiskers specify samples comprised 1.5 times the interquartile range. Statistic: Wilcoxon matched pair test. *p≤0.05, **p≤0.01, ***p≤0.001, ****p≤0.0001.

**Supplementary tables**

**Supplementary table 1: list of cell lines used in this study**. For each cell line, MYCN-status, source, data obtained, mycoplasma testing, citation are listed. Information about the patient from whom are derived the cell line are provided in the table: site of tumor (site), tumor stage (stage), age of the patient, phase of the therapy, MYCN amplification status and p53 mutation status.

**Supplementary table 2: List of primers used in this study.** Sense and anti-sense sequences are provided. First column represents the gene name.

**Supplementary table 3: clinical information for neuroblastoma dataset (accession: E-MTAB-1781).** Data are obtained from the dataset associated meta-data.

**Supplementary table4: clinical information for neuroblastoma dataset (accession: TARGET).** Data are obtained from the dataset associated meta-data.

**Supplementary table 5: differentiation gene signature.** List of gene in the differentiation gene signature used in the analysis. Genes in the list are obtained as described in the methods above.

**Supplementary table 6: Convolutional Neural Network Model.** Layers, output and parameters are listed.

**Supplementary table 7: Convolutional Neural Network statistics.** True Positive, True Negative, False Positive, False Negative, Specificity, Sensitivity, Misclassification metrics are listed in the table.

**Supplementary table 8: List of the transcription factor for the GRN.**

**Supplementary table 9: List of the 50 regulons in the NB1 cohort.** (NB1: E-MTAB-1781). In the table the regulon name, the regulon size (number of gene inside the regulon), absolute value are listed.

**Supplementary table 10: List of the 50 regulons in the NB2 cohort.** (NB2: TARGET neuroblastoma). In the table the regulon name, the regulon size (number of gene inside the regulon), absolute value are listed.

**Supplementary table 11: list of the gene present in each regulon.** The first column lists the regulon name, in the second column the gene present in the regulon and in the third column the cluster of the regulon.

**Supplementary table 12: List of the first pathways enriched in each regulon cluster.**

**Supplementary table 13: clinical information for neuroblastoma dataset (accession: E-MTAB-1781).** Data are obtained from the dataset associated meta-data. MYCN status columns represent amplification status (MNA for MYCN amplification, non-MNA for not amplified), OS_d represents overall survivals, OS_bin (Overall survival status, 1 death, 0 censored). Reg_group, represent the regulon group after the clustering on the regulon activity.

**Supplementary table 14: clinical information for neuroblastoma dataset (accession: TARGET).** Data are obtained from the dataset associated meta-data. MYCN status column represents amplification status (MNA for MYCN amplification, non-MNA for not amplified), OS_d represents overall survivals, OS_bin (Overall surviva status, 1 death, 0 censored). Reg_group, represent the regulon group after the clustering on the regulon activity.

**Supplementary table 15: list of gene in the mTOR pathway signature**.

**Supplementary table 16: List of genes used for the univariate Cox regression.**

**Supplementary table 17: List of significant genes: univariate Cox regression.** Gene names are indicated in the first column. Beta column indicates the coefficient, HR (hazard ratio), HR (95% CI for HR) indicates hazard ratio and confidence interval for 95%, p.value indicates the obtained p value, p significant if p value is less than 0.05 (true indicates the p value is significant, false if it is not significant), psignbonf if the p value is still significant after Bonferroni correction for multi testing (true if still significant, false if not).

**Supplementary table 18: List of genes: penalized Cox regression.** The list indicates the gene which the coefficient is not zero after the penalized Cox regression. The first column contains the gene name, the second the associated coefficient.

**Supplementary table 19: clinical information for neuroblastoma dataset (accession: E-MTAB-1781).** MYCN status columns represent amplification status (MNA for MYCN amplification, non-MNA for not amplified), sex column, Age (in days), OS_d represents overall survivals, OS_bin (Overall survival status, 1 death, 0 censored), Stage (2A and 2B collapsed as 2), diffp (differentiation positive score), diffn (differentiation negative score), diffs (differentiation score), diffnorm (differentiation score normalized, z-score), diff (differentiation status group stratification according to the differentiation score), mtors (mTOR pathway activity score normalized, z-score), diff (mTOR status group stratification according to the mTOR activity score)

**Supplementary table 20: Lysosome raw data and statistical analysis.**

**Supplementary table 21: animal weights.** The table presents information about the treated mice in this study. For each mouse we provide the number of reference of the mouse in the study, the treatment, the weight at the start, the weight at the end of the study, the percentage of body weight variation at the end of the study.

**Supplementary Bibliography**

1. Chou T-C. Theoretical basis, experimental design, and computerized simulation of synergism and antagonism in drug combination studies. Pharmacol Rev. 2006;58:621–81.

2. Zhang N, Fu J-N, Chou T-C. Synergistic combination of microtubule targeting anticancer fludelone with cytoprotective panaxytriol derived from panax ginseng against MX-1 cells in vitro: experimental design and data analysis using the combination index method. Am J Cancer Res. 2016;6:97–104.

3. Chou T-C. The combination index (CI < 1) as the definition of synergism and of synergy claims. Synergy. 2018;7:49–50.

4. Valente AJ, Maddalena LA, Robb EL, Moradi F, Stuart JA. A simple ImageJ macro tool for analyzing mitochondrial network morphology in mammalian cell culture. Acta Histochem. 2017;119:315–26.

5. Jakic B, Buszko M, Cappellano G, Wick G. Elevated sodium leads to the increased expression of HSP60 and induces apoptosis in HUVECs. PLOS ONE. Public Library of Science; 2017;12:e0179383.

6. McCloy RA, Rogers S, Caldon CE, Lorca T, Castro A, Burgess A. Partial inhibition of Cdk1 in G2 phase overrides the SAC and decouples mitotic events. Cell Cycle. 2014;13:1400–12.

7. Oberthuer A, Juraeva D, Hero B, Volland R, Sterz C, Schmidt R, et al. Revised Risk Estimation and Treatment Stratification of Low- and Intermediate-Risk Neuroblastoma Patients by Integrating Clinical and Molecular Prognostic Markers. Clin Cancer Res. 2015;21:1904–15.

8. Ritchie ME, Phipson B, Wu D, Hu Y, Law CW, Shi W, et al. limma powers differential expression analyses for RNA-sequencing and microarray studies. Nucleic Acids Research. 2015;43:e47–e47.

9. Pugh TJ, Morozova O, Attiyeh EF, Asgharzadeh S, Wei JS, Auclair D, et al. The genetic landscape of high-risk neuroblastoma. Nat Genet. 2013;45:279–84.

10. Subramanian A, Tamayo P, Mootha VK, Mukherjee S, Ebert BL, Gillette MA, et al. Gene set enrichment analysis: A knowledge-based approach for interpreting genome-wide expression profiles. PNAS. National Academy of Sciences; 2005;102:15545–50.

11. Mootha VK, Lindgren CM, Eriksson K-F, Subramanian A, Sihag S, Lehar J, et al. PGC-1α-responsive genes involved in oxidative phosphorylation are coordinately downregulated in human diabetes. Nature Genetics. Nature Publishing Group; 2003;34:267–73.

12. Supek F, Bošnjak M, Škunca N, Šmuc T. REVIGO Summarizes and Visualizes Long Lists of Gene Ontology Terms. PLOS ONE. Public Library of Science; 2011;6:e21800.

13. Pesquita C, Faria D, Falcão AO, Lord P, Couto FM. Semantic Similarity in Biomedical Ontologies. PLOS Computational Biology. Public Library of Science; 2009;5:e1000443.

14. GO.db [Internet]. Bioconductor. [cited 2021 Jun 15]. Available from: http://bioconductor.org/packages/GO.db/

15. Bederson B. Ordered and Quantum Treemaps: Making Effective Use of 2D Space to Display Hierarchies. ACM Transactions on Graphics,. 2002;833–54.

16. Bruls D. Squarified Treemaps. Proceedings of the joint Eurographics and IEEE TCVG Symposium on Visualization. 2000;33–42.

17. Becht E, McInnes L, Healy J, Dutertre C-A, Kwok IWH, Ng LG, et al. Dimensionality reduction for visualizing single-cell data using UMAP. Nature Biotechnology. Nature Publishing Group; 2019;37:38–44.

18. McInnes L, Healy J, Melville J. UMAP: Uniform Manifold Approximation and Projection for Dimension Reduction. arXiv:180203426 [cs, stat] [Internet]. 2020 [cited 2020 Oct 28]; Available from: http://arxiv.org/abs/1802.03426

19. Campello RJGB, Moulavi D, Sander J. Density-Based Clustering Based on Hierarchical Density Estimates. In: Pei J, Tseng VS, Cao L, Motoda H, Xu G, editors. Advances in Knowledge Discovery and Data Mining. Berlin, Heidelberg: Springer; 2013. page 160–72.

20. McInnes L, Healy J, Astels S. hdbscan: Hierarchical density based clustering. Journal of Open Source Software. 2017;2:205.

21. He K, Zhang X, Ren S, Sun J. Deep Residual Learning for Image Recognition. arXiv:151203385 [cs] [Internet]. 2015 [cited 2021 Jan 26]; Available from: http://arxiv.org/abs/1512.03385

22. Zhuang F, Qi Z, Duan K, Xi D, Zhu Y, Zhu H, et al. A Comprehensive Survey on Transfer Learning. arXiv:191102685 [cs, stat] [Internet]. 2020 [cited 2021 Jan 26]; Available from: http://arxiv.org/abs/1911.02685

23. Zou F, Shen L, Jie Z, Zhang W, Liu W. A Sufficient Condition for Convergences of Adam and RMSProp. 2019 [cited 2022 Apr 10]. page 11127–35. Available from: https://openaccess.thecvf.com/content_CVPR_2019/html/Zou_A_Sufficient_Condition_for_Convergences_of_Adam_and_RMSProp_CVPR_2019_paper.html

24. Mukkamala MC, Hein M. Variants of RMSProp and Adagrad with Logarithmic Regret Bounds. Proceedings of the 34th International Conference on Machine Learning [Internet]. PMLR; 2017 [cited 2022 Apr 10]. page 2545–53. Available from: https://proceedings.mlr.press/v70/mukkamala17a.html

25. Kurbiel T, Khaleghian S. Training of Deep Neural Networks based on Distance Measures using RMSProp. arXiv:170801911 [cs, stat] [Internet]. 2017 [cited 2022 Apr 10]; Available from: http://arxiv.org/abs/1708.01911

26. De S, Mukherjee A, Ullah E. Convergence guarantees for RMSProp and ADAM in non-convex optimization and an empirical comparison to Nesterov acceleration. arXiv:180706766 [cs, math, stat] [Internet]. 2018 [cited 2022 Apr 10]; Available from: http://arxiv.org/abs/1807.06766

27. Shi N, Li D, Hong M, Sun R. RMSprop converges with proper hyper-parameter. 2020 [cited 2022 Apr 10]. Available from: https://openreview.net/forum?id=3UDSdyIcBDA

28. Li X, Orabona F. On the Convergence of Stochastic Gradient Descent with Adaptive Stepsizes. arXiv:180508114 [cs, math, stat] [Internet]. 2019 [cited 2022 Apr 10]; Available from: http://arxiv.org/abs/1805.08114

29. Gulcehre C, Sotelo J, Moczulski M, Bengio Y. A Robust Adaptive Stochastic Gradient Method for Deep Learning. arXiv:170300788 [cs] [Internet]. 2017 [cited 2022 Apr 10]; Available from: http://arxiv.org/abs/1703.00788

30. Saxena S, Shukla S, Gyanchandani M. Pre-trained convolutional neural networks as feature extractors for diagnosis of breast cancer using histopathology. International Journal of Imaging Systems and Technology. 2020;30:577–91.

31. Lopes UK, Valiati JF. Pre-trained convolutional neural networks as feature extractors for tuberculosis detection. Computers in Biology and Medicine. 2017;89:135–43.

32. Guérin J, Boots B. Improving Image Clustering With Multiple Pretrained CNN Feature Extractors. arXiv:180707760 [cs] [Internet]. 2018 [cited 2022 Apr 10]; Available from: http://arxiv.org/abs/1807.07760

33. Guérin J, Thiery S, Nyiri E, Gibaru O, Boots B. Combining pretrained CNN feature extractors to enhance clustering of complex natural images. Neurocomputing. 2021;423:551–71.

34. Nishida Y, Adati N, Ozawa R, Maeda A, Sakaki Y, Takeda T. Identification and classification of genes regulated by phosphatidylinositol 3-kinase- and TRKB-mediated signalling pathways during neuronal differentiation in two subtypes of the human neuroblastoma cell line SH-SY5Y. BMC Res Notes. 2008;1:95.

35. Huynh-Thu VA, Irrthum A, Wehenkel L, Geurts P. Inferring Regulatory Networks from Expression Data Using Tree-Based Methods. PLOS ONE. Public Library of Science; 2010;5:e12776.

36. Ashtiani M, Mirzaie M, Jafari M. CINNA: an R/CRAN package to decipher Central Informative Nodes in Network Analysis. Bioinformatics. 2019;35:1436–7.

37. Kobak D, Berens P. The art of using t-SNE for single-cell transcriptomics. Nature Communications. Nature Publishing Group; 2019;10:5416.

38. Fletcher MNC, Castro MAA, Wang X, de Santiago I, O’Reilly M, Chin S-F, et al. Master regulators of FGFR2 signalling and breast cancer risk. Nature Communications. Nature Publishing Group; 2013;4:2464.

39. Castro MAA, de Santiago I, Campbell TM, Vaughn C, Hickey TE, Ross E, et al. Regulators of genetic risk of breast cancer identified by integrative network analysis. Nature Genetics. Nature Publishing Group; 2016;48:12–21.

40. Groeneveld CS, Chagas VS, Jones SJM, Robertson AG, Ponder BAJ, Meyer KB, et al. RTNsurvival: an R/Bioconductor package for regulatory network survival analysis. Bioinformatics. 2019;35:4488–9.

41. Therneau TM, Grambsch PM. Modeling Survival Data: Extending the Cox Model [Internet]. New York: Springer-Verlag; 2000 [cited 2021 May 25]. Available from: https://www.springer.com/gp/book/9780387987842

42. Russell RC, Fang C, Guan K-L. An emerging role for TOR signaling in mammalian tissue and stem cell physiology. Development. 2011;138:3343–56.

43. Mossmann D, Park S, Hall MN. mTOR signalling and cellular metabolism are mutual determinants in cancer. Nature Reviews Cancer. Nature Publishing Group; 2018;18:744–57.

44. Robbins HL, Hague A. The PI3K/Akt Pathway in Tumors of Endocrine Tissues. Front Endocrinol (Lausanne) [Internet]. 2016 [cited 2021 May 31];6. Available from: https://www.ncbi.nlm.nih.gov/pmc/articles/PMC4707207/

45. Zou Z, Tao T, Li H, Zhu X. mTOR signaling pathway and mTOR inhibitors in cancer: progress and challenges. Cell Biosci [Internet]. 2020 [cited 2021 May 31];10. Available from: https://www.ncbi.nlm.nih.gov/pmc/articles/PMC7063815/

46. LiCausi F, Hartman NW. Role of mTOR Complexes in Neurogenesis. Int J Mol Sci [Internet]. 2018 [cited 2021 May 31];19. Available from: https://www.ncbi.nlm.nih.gov/pmc/articles/PMC5983636/

47. Sittewelle M, Monsoro-Burq AH. AKT signaling displays multifaceted functions in neural crest development. Developmental Biology. 2018;444:S144–55.

48. Mei H, Wang Y, Lin Z, Tong Q. The mTOR signaling pathway in pediatric neuroblastoma. Pediatr Hematol Oncol. 2013;30:605–15.

49. Yue M, Jiang J, Gao P, Liu H, Qing G. Oncogenic MYC Activates a Feedforward Regulatory Loop Promoting Essential Amino Acid Metabolism and Tumorigenesis. Cell Reports. Elsevier; 2017;21:3819–32.

50. Salisbury TB, Arthur S. The Regulation and Function of the L-Type Amino Acid Transporter 1 (LAT1) in Cancer. Int J Mol Sci [Internet]. 2018 [cited 2021 May 31];19. Available from: https://www.ncbi.nlm.nih.gov/pmc/articles/PMC6121554/

51. Barretina J, Caponigro G, Stransky N, Venkatesan K, Margolin AA, Kim S, et al. The Cancer Cell Line Encyclopedia enables predictive modelling of anticancer drug sensitivity. Nature. Nature Publishing Group; 2012;483:603–7.

52. Ghandi M, Huang FW, Jané-Valbuena J, Kryukov GV, Lo CC, McDonald ER, et al. Next-generation characterization of the Cancer Cell Line Encyclopedia. Nature. Nature Publishing Group; 2019;569:503–8.

53. AnimalTFDB 3.0: a comprehensive resource for annotation and prediction of animal transcription factors | Nucleic Acids Research | Oxford Academic [Internet]. [cited 2020 Jun 16]. Available from: https://academic.oup.com/nar/article/47/D1/D33/5094755?guestAccessKey=d0b5ab2d-e4ea-4b97-a181-51b578f1fa83

54. Szklarczyk D, Gable AL, Lyon D, Junge A, Wyder S, Huerta-Cepas J, et al. STRING v11: protein-protein association networks with increased coverage, supporting functional discovery in genome-wide experimental datasets. Nucleic Acids Res. 2019;47:D607–13.

55. Zeid R, Lawlor MA, Poon E, Reyes JM, Fulciniti M, Lopez MA, et al. Enhancer invasion shapes MYCN dependent transcriptional amplification in neuroblastoma. Nat Genet. 2018;50:515–23.

56. Robinson JT, Thorvaldsdóttir H, Winckler W, Guttman M, Lander ES, Getz G, et al. Integrative Genomics Viewer. Nat Biotechnol. 2011;29:24–6.

57. Thorvaldsdóttir H, Robinson JT, Mesirov JP. Integrative Genomics Viewer (IGV): high-performance genomics data visualization and exploration. Briefings in Bioinformatics. 2013;14:178–92.

58. Iorio F, Knijnenburg TA, Vis DJ, Bignell GR, Menden MP, Schubert M, et al. A Landscape of Pharmacogenomic Interactions in Cancer. Cell. Elsevier; 2016;166:740–54.

59. Raieli S, Di Renzo D, Lampis S, Amadesi C, Montemurro L, Pession A, et al. MYCN Drives a Tumor Immunosuppressive Environment Which Impacts Survival in Neuroblastoma. Front Oncol. 2021;11:625207.

60. Goeman JJ. L1 penalized estimation in the Cox proportional hazards model. Biom J. 2010;52:70–84.

61. Heagerty PJ, Zheng Y. Survival Model Predictive Accuracy and ROC Curves. Biometrics. 2005;61:92–105.

62. Heagerty PJ, Lumley T, Pepe MS. Time-Dependent ROC Curves for Censored Survival Data and a Diagnostic Marker. Biometrics. 2000;56:337–44.

63. Schindelin J, Arganda-Carreras I, Frise E, Kaynig V, Longair M, Pietzsch T, et al. Fiji: an open-source platform for biological-image analysis. Nature Methods. Nature Publishing Group; 2012;9:676–82.

64. Schneider CA, Rasband WS, Eliceiri KW. NIH Image to ImageJ: 25 years of image analysis. Nature Methods. Nature Publishing Group; 2012;9:671–5.

65. Rueden CT, Schindelin J, Hiner MC, DeZonia BE, Walter AE, Arena ET, et al. ImageJ2: ImageJ for the next generation of scientific image data. BMC Bioinformatics. 2017;18:529.

66. Ruifrok AC, Johnston DA. Quantification of histochemical staining by color deconvolution. Anal Quant Cytol Histol. 2001;23:291–9.

67. Walt S van der, Schönberger JL, Nunez-Iglesias J, Boulogne F, Warner JD, Yager N, et al. scikit-image: image processing in Python. PeerJ. PeerJ Inc.; 2014;2:e453.
